# Supplementary figures and images for: The curriculum effect in visual learning: The role of readout dimensionality (part 1 of 2)
Source: PLoS Comput Biol. 2026 Jul 24;22(7):e1014553. doi: 10.1371/journal.pcbi.1014553 (PMC13432104; doi:10.1371/journal.pcbi.1014553)

A

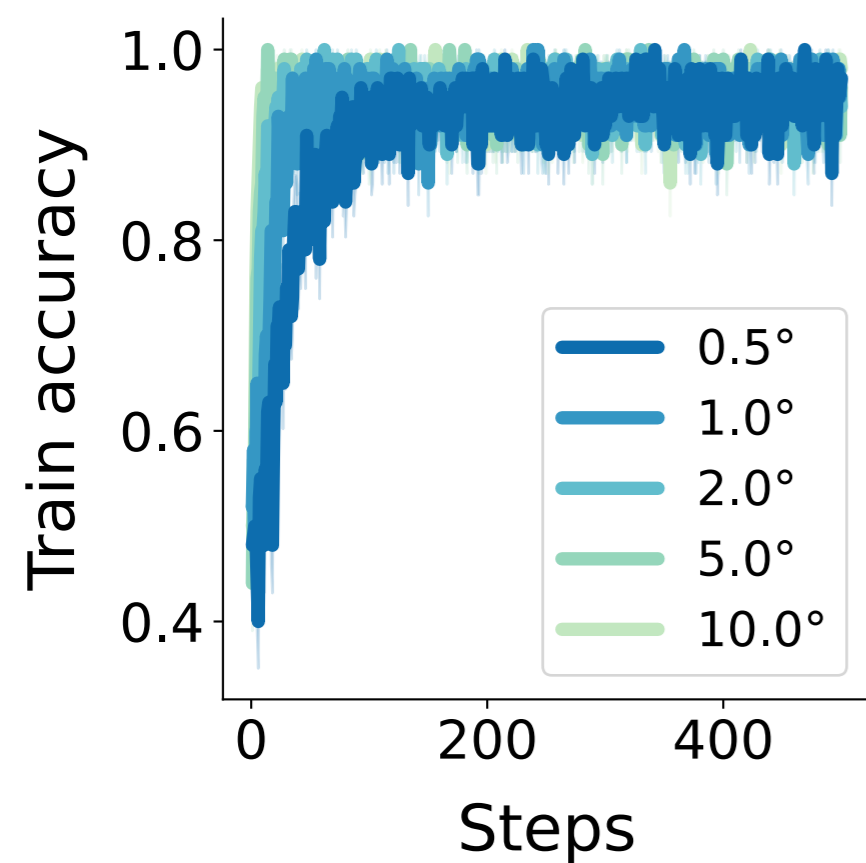

B

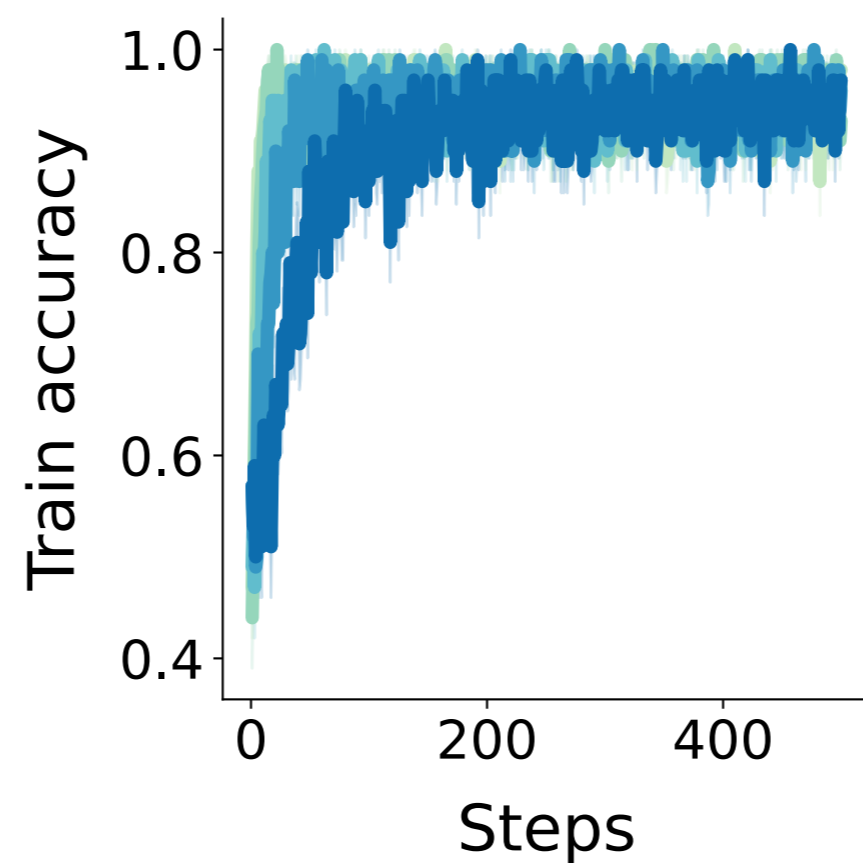

C

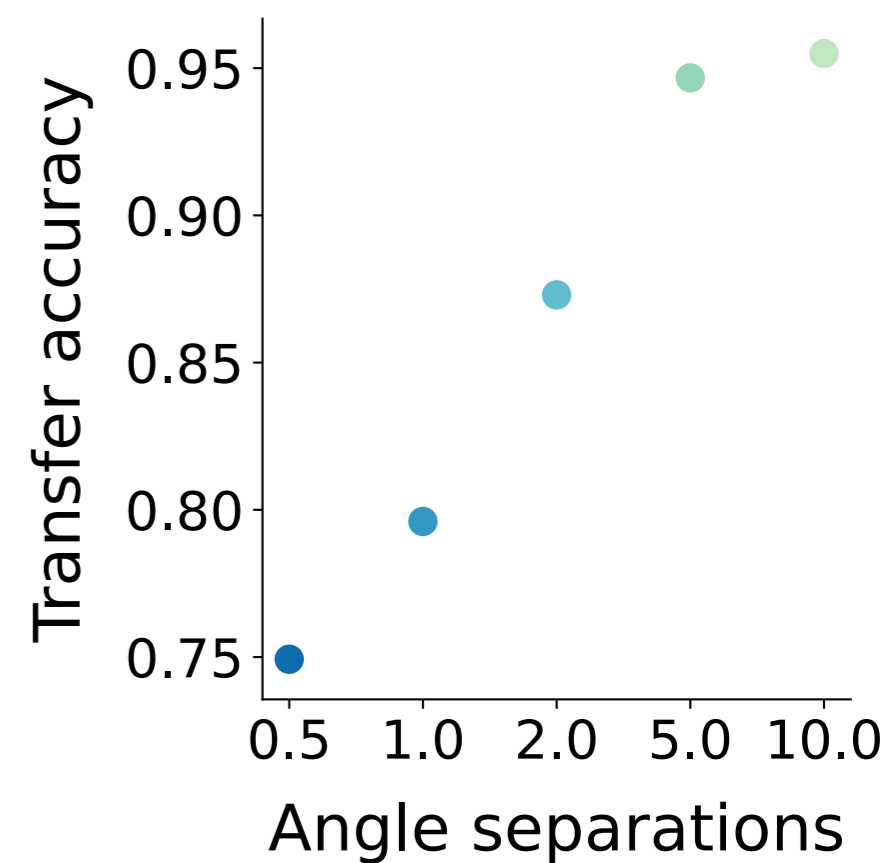

D

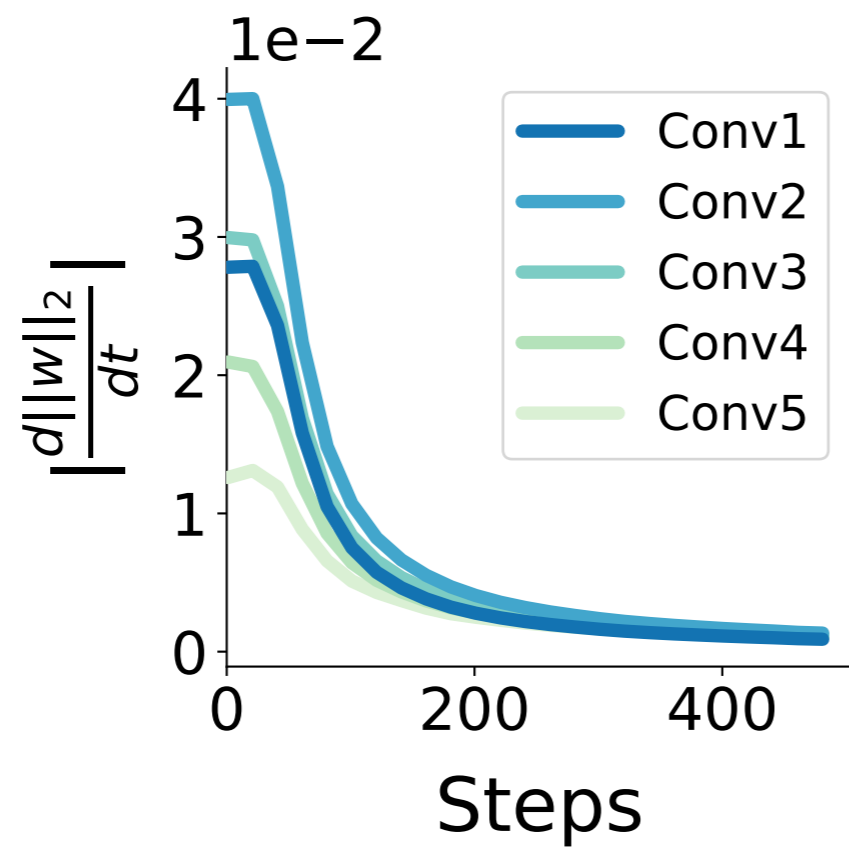

E

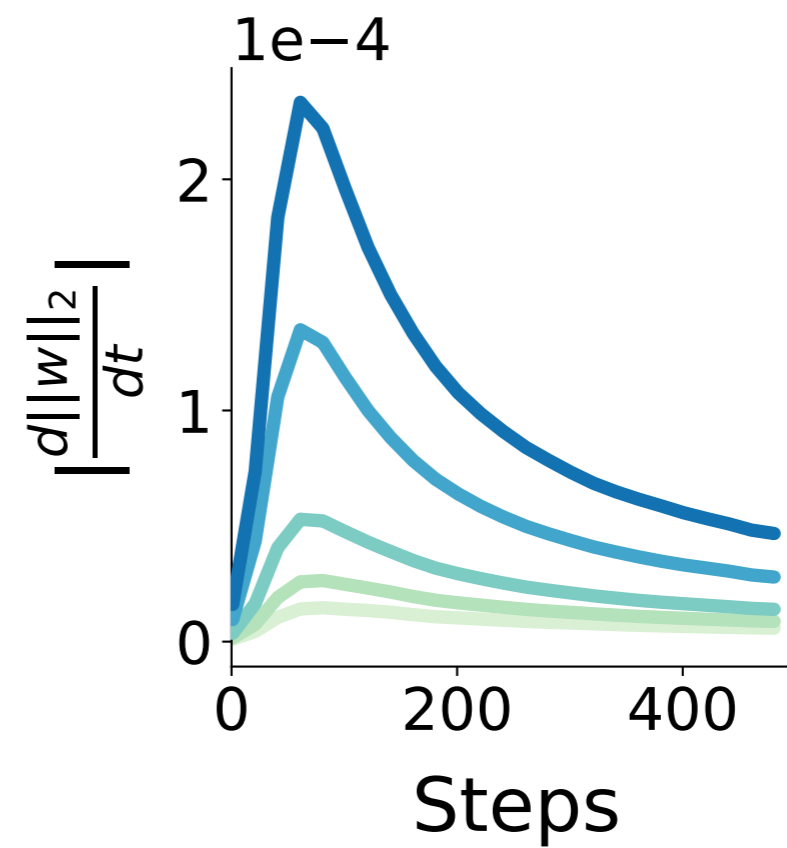

Supplement: S1 Fig — (A) Train accuracy with standard error visible. (B) Train accuracy with standard error visible for a model with convolutional and pooling layer weights frozen and readout weights unfrozen. (C) Transfer accuracy for the model from B. (D) Gradient of the weight norm of skip connection weights throughout training on a 0.5° angle separation. (E) Gradient of the weight norm of convolutional weights throughout training on a 0.5° angle separation. (PDF) [file pcbi.1014553.s001.pdf]

A

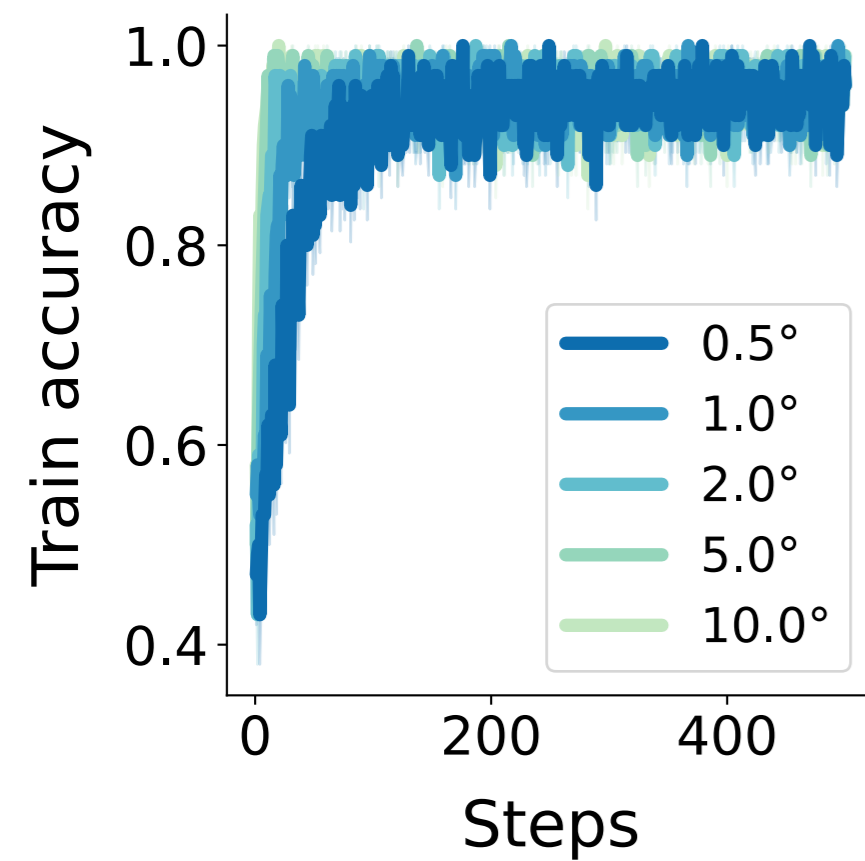

B

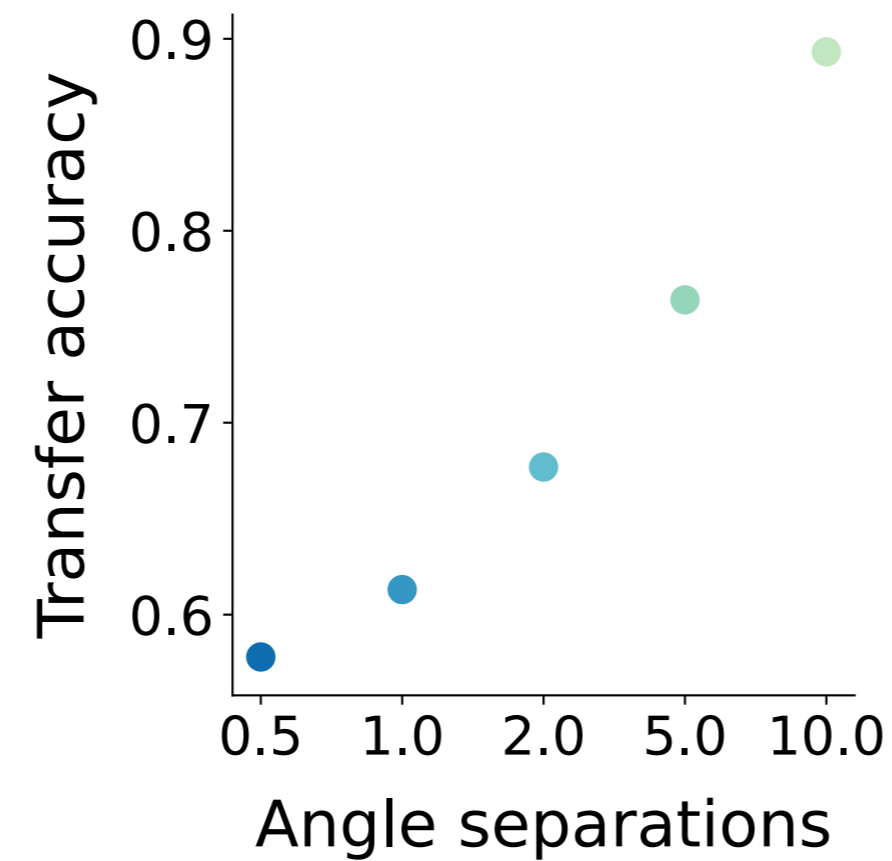

C

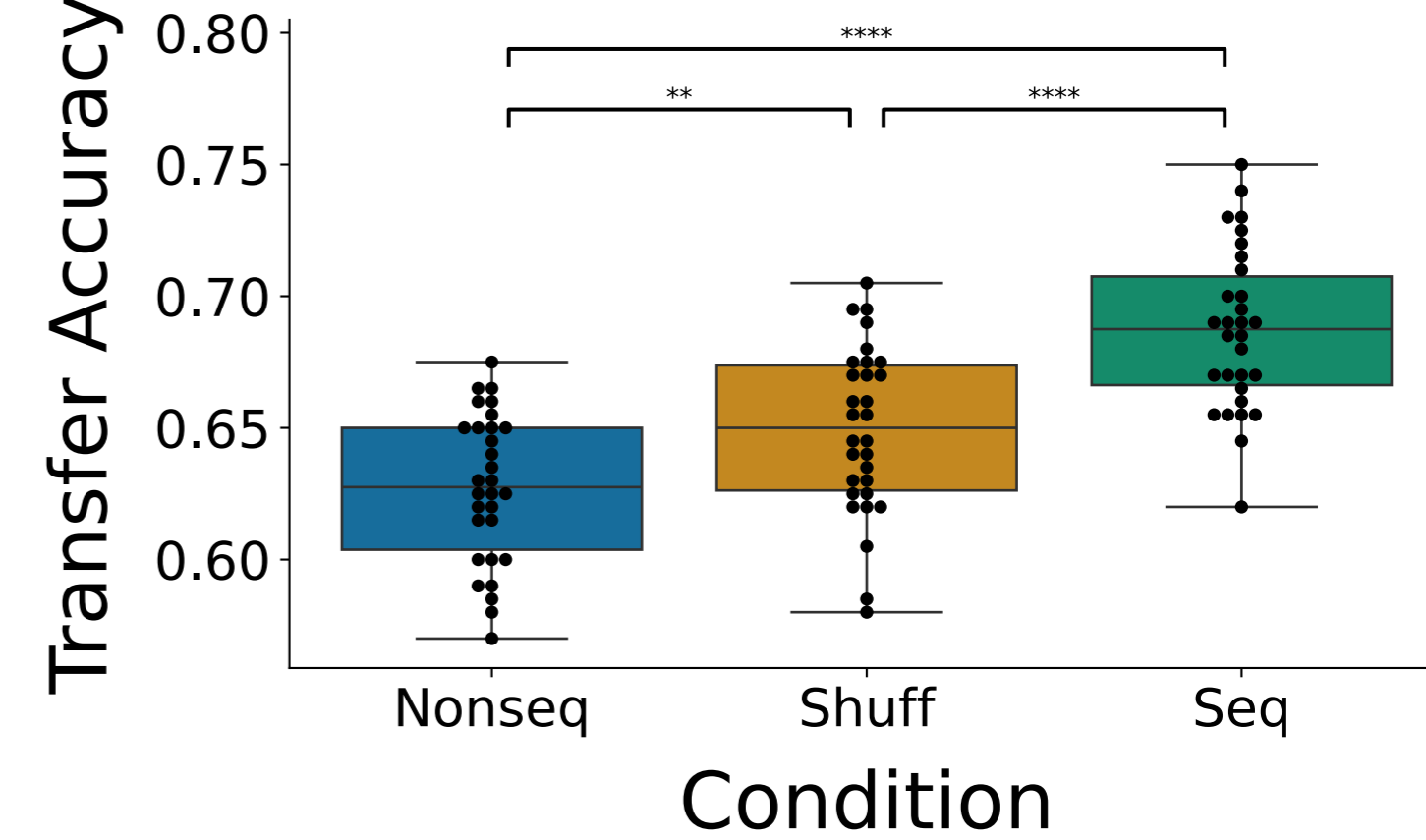

D

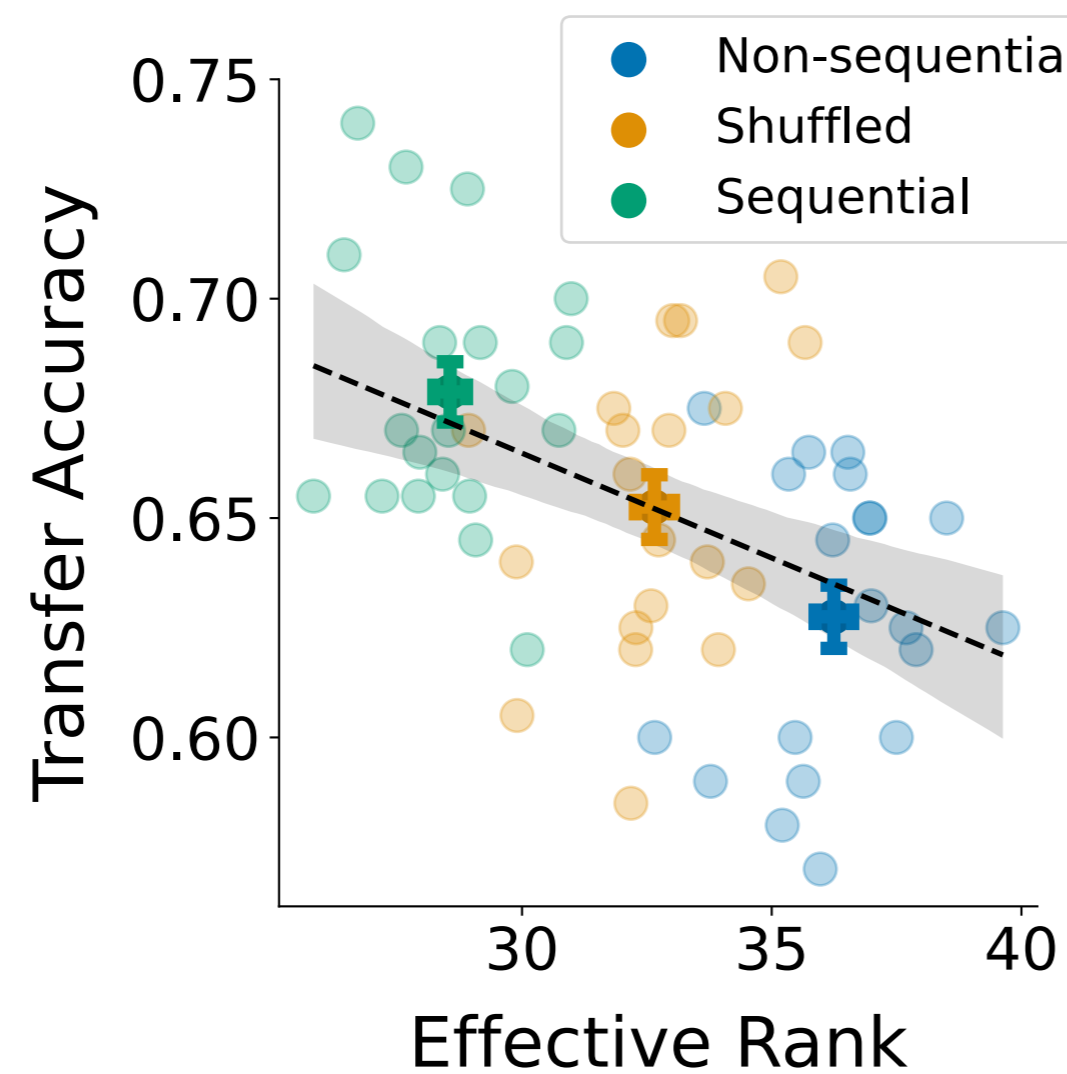

E

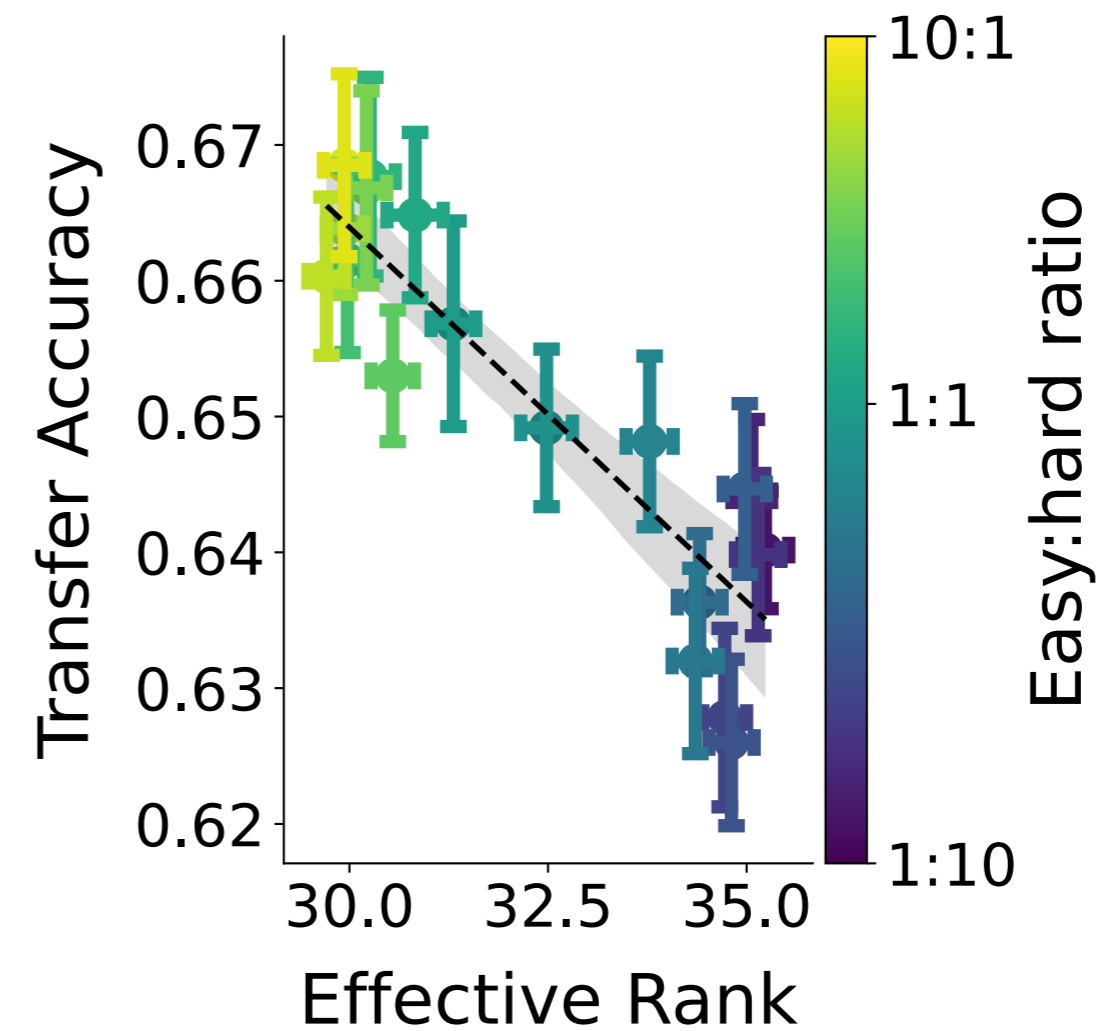

Supplement: S2 Fig — (A) Train accuracy. (B) Transfer accuracy. (C) Transfer accuracy for models across curricula. (D) Transfer accuracy versus effective rank for models across curricula (r = -0.45, p = 0.00034). (E) Negative correlation of the effective rank for shuffled curricula with different easy:hard sample ratios with transfer accuracy (r = -0.89, p<<0.0001). (PDF) [file pcbi.1014553.s002.pdf]

A

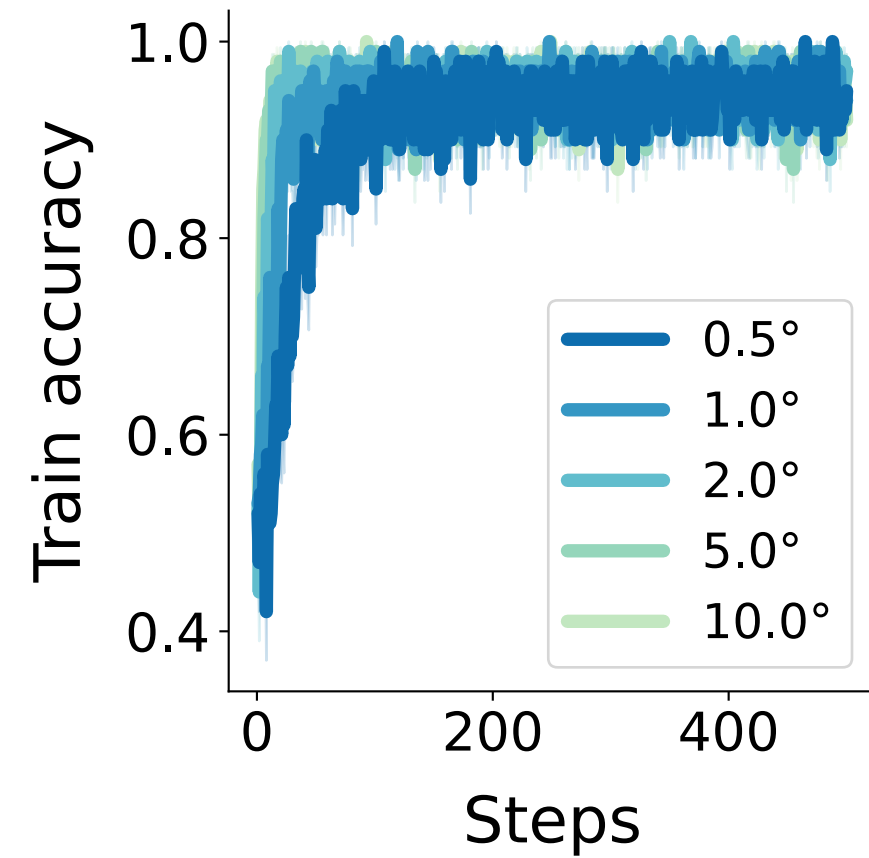

B

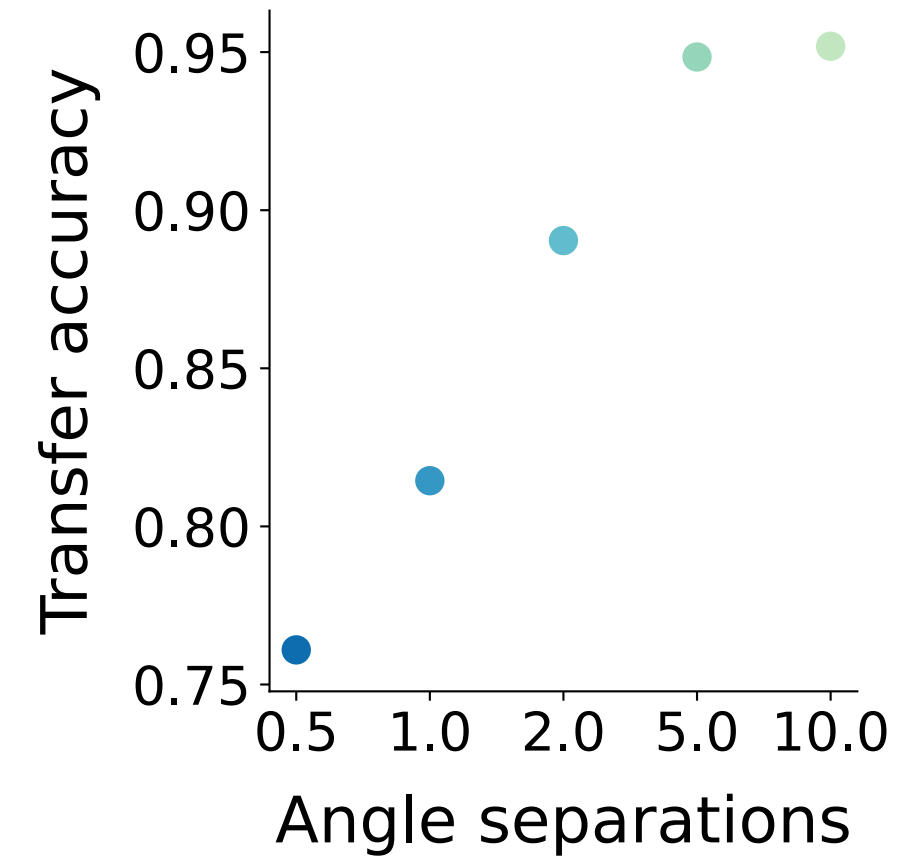

C

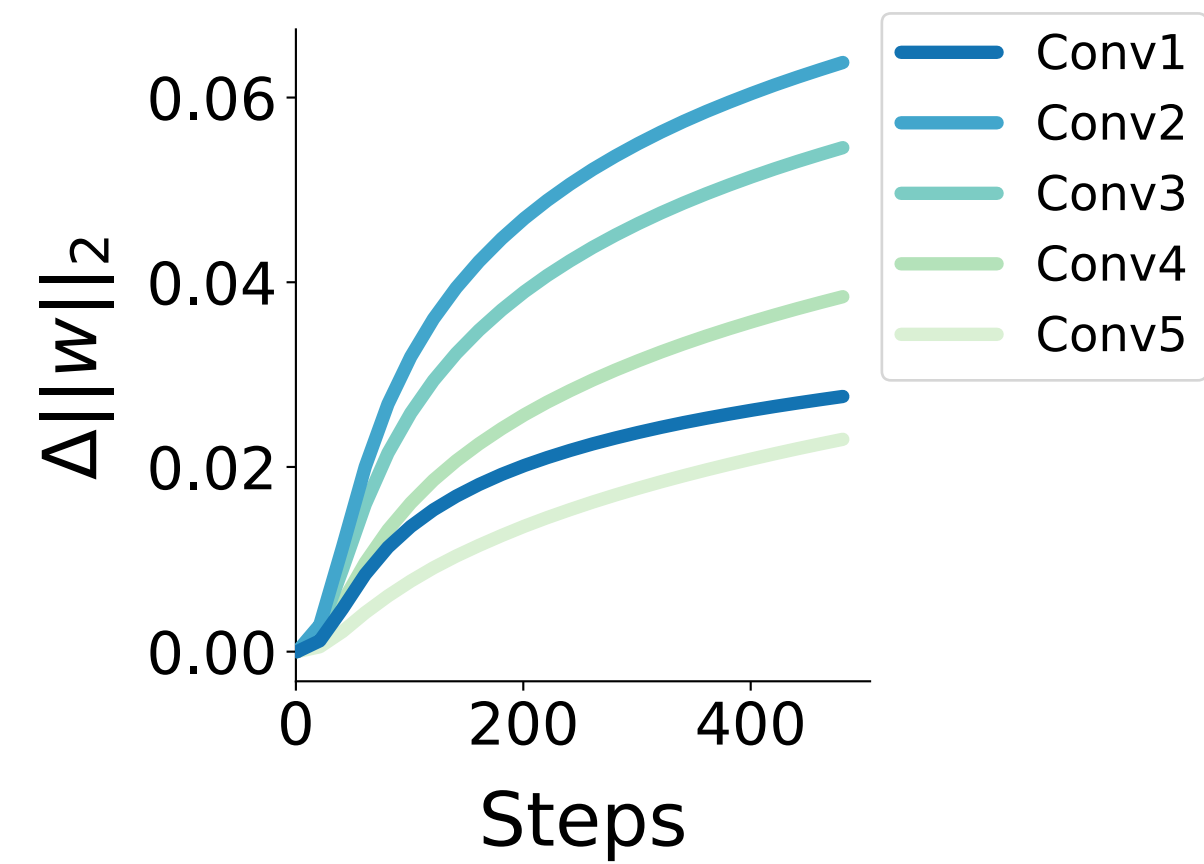

D

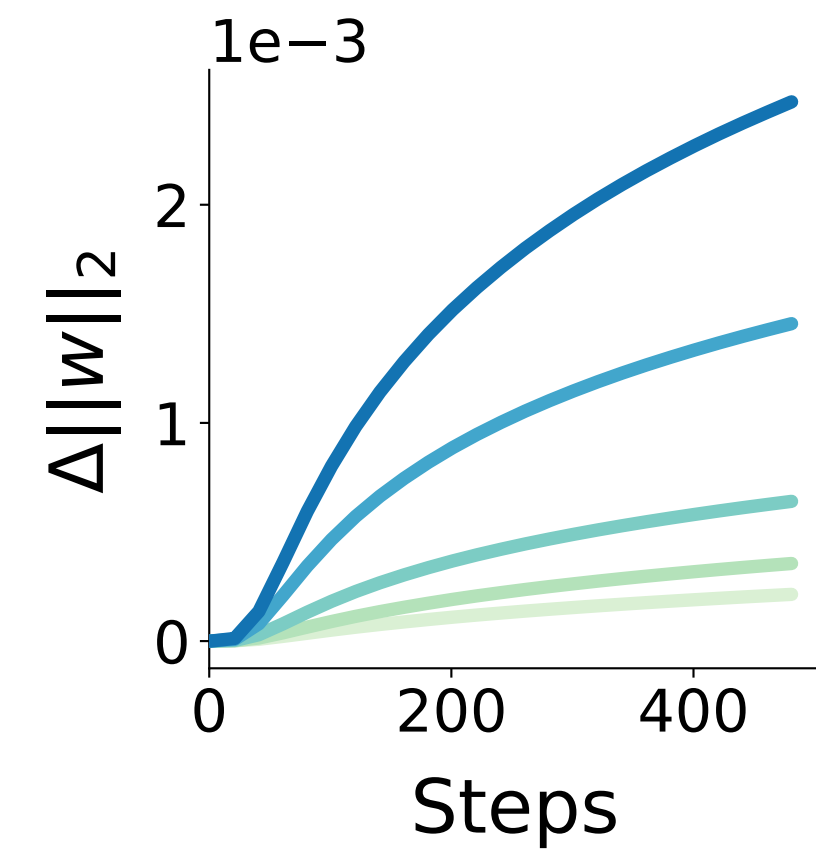

E

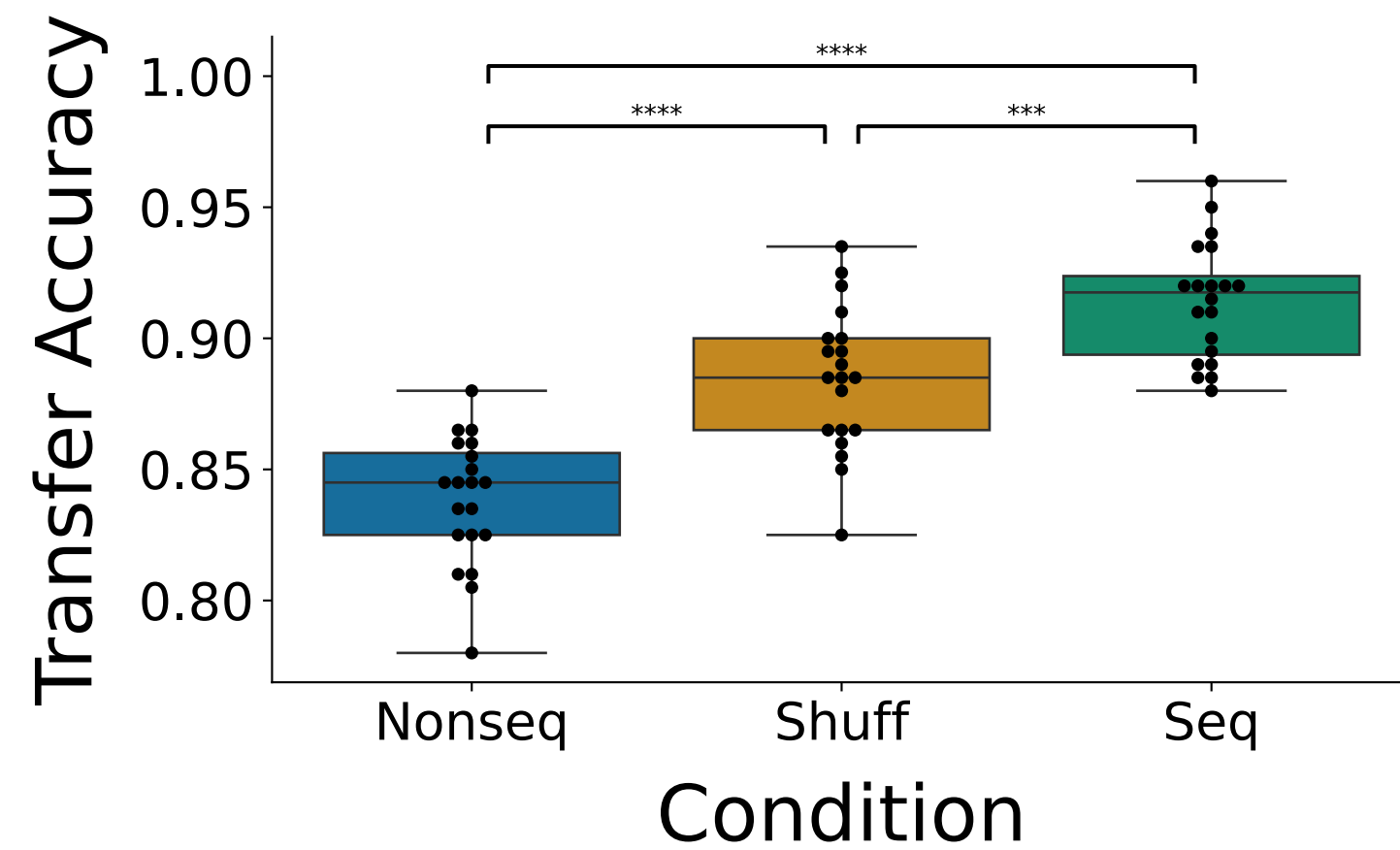

F

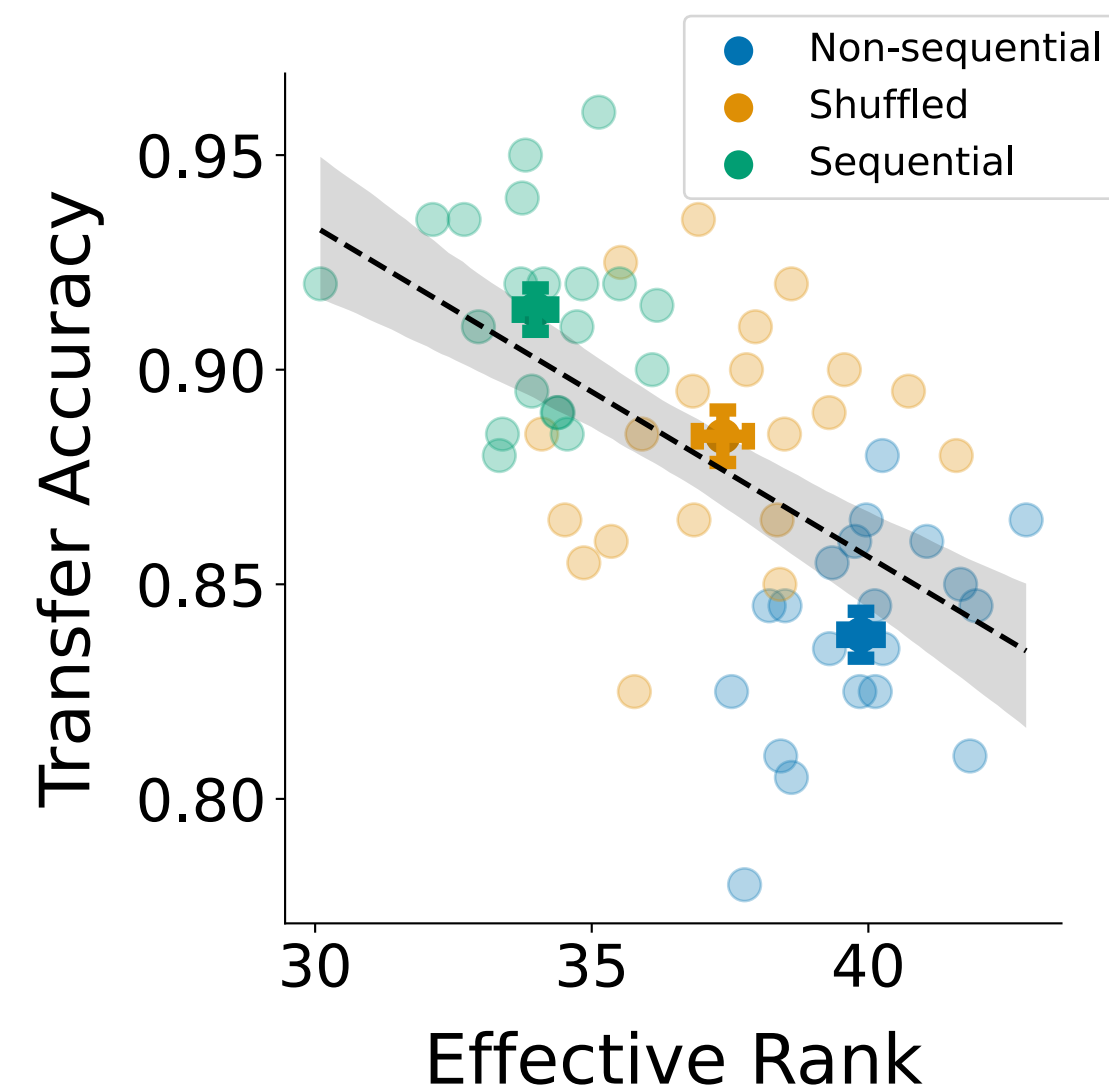

G

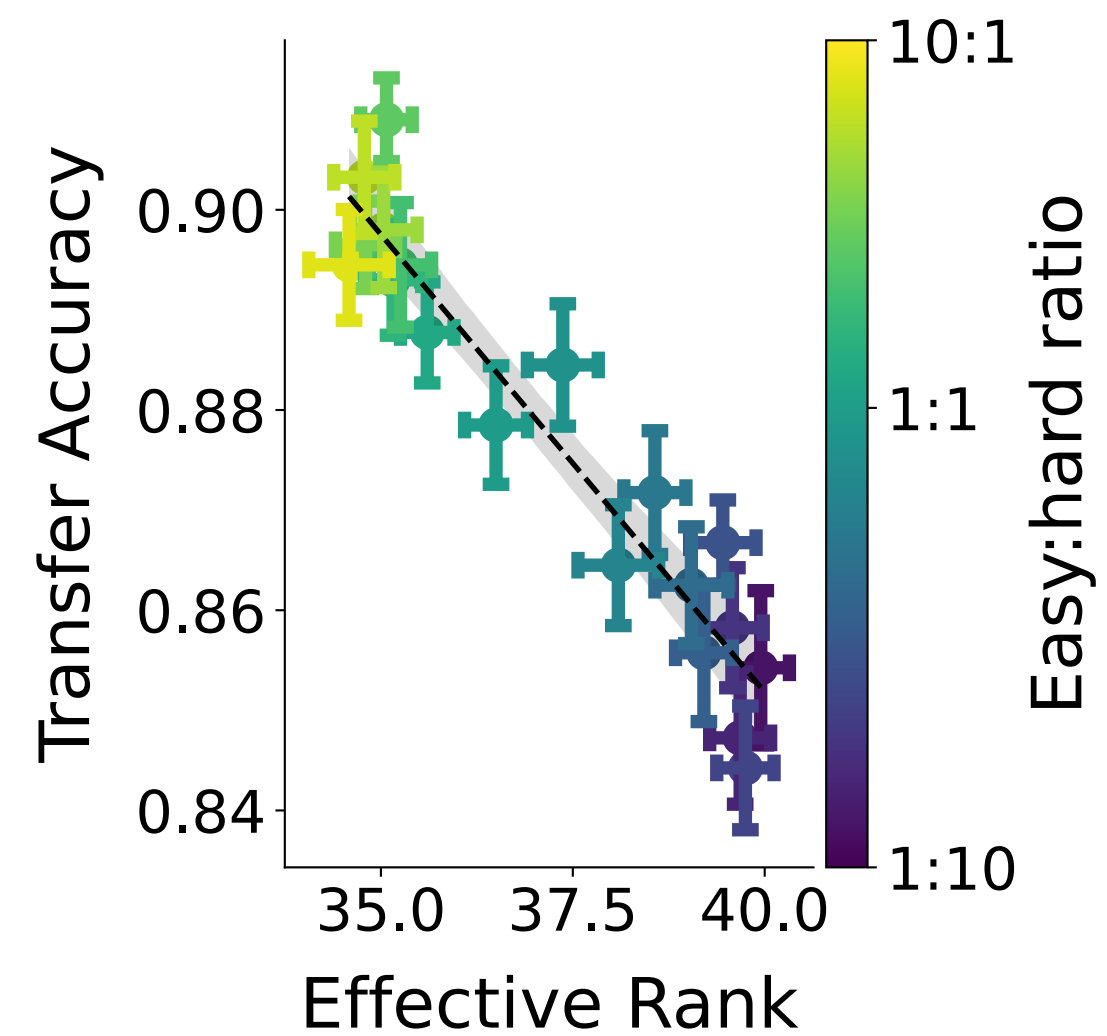

Supplement: S3 Fig — (A) Train accuracy. (B) Transfer accuracy. (C) Change in the L2-norm of skip connection weights throughout training on a 0.5° angle separation. (D) Change in the L2-norm of convolutional weights throughout training on a 0.5° angle separation. (E) Transfer accuracy for models across curricula. (F) Transfer accuracy versus effective rank for models across curricula (r = -0.57, p<<0.0001). (G) Negative correlation of the effective rank for shuffled curricula with different easy:hard sample ratios with transfer accuracy (r = -0.95, p<<0.0001). (PDF) [file pcbi.1014553.s003.pdf]

A

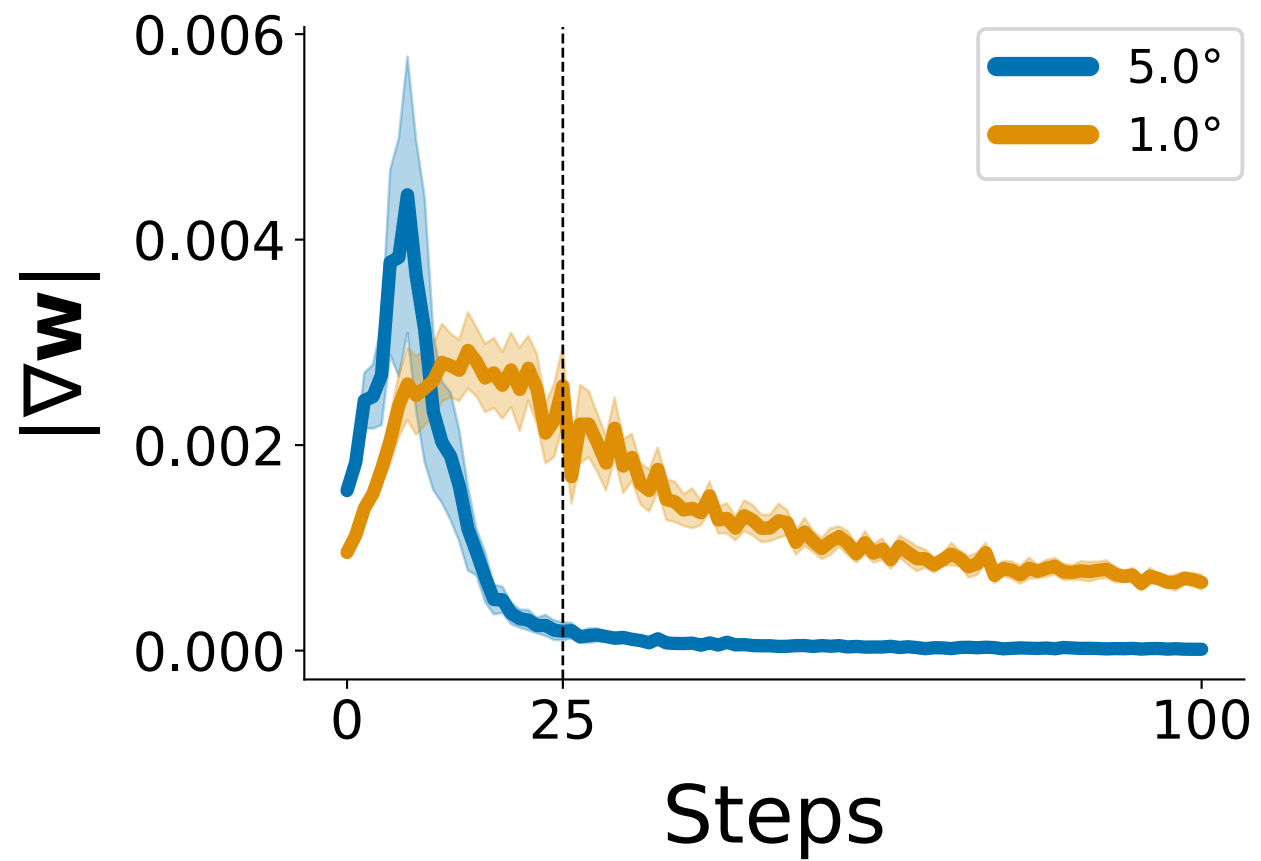

B

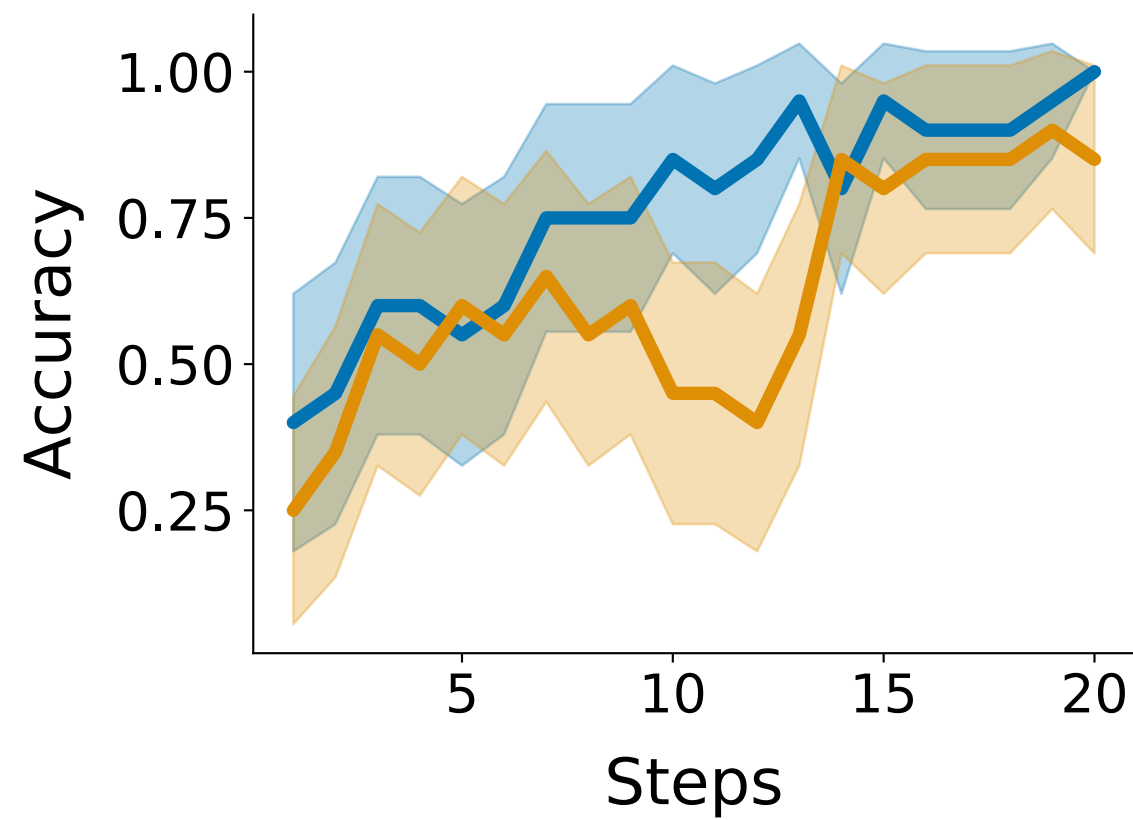

Supplement: S4 Fig — (A) Parameter gradients for models trained on the shuffled task condition in the first 100 steps of training. This plot includes gradients for the non-active condition at each timestep. (B) Accuracy throughout training for models on the shuffled condition shown separately for easy (5°) and hard (1°) samples. (PDF) [file pcbi.1014553.s004.pdf]

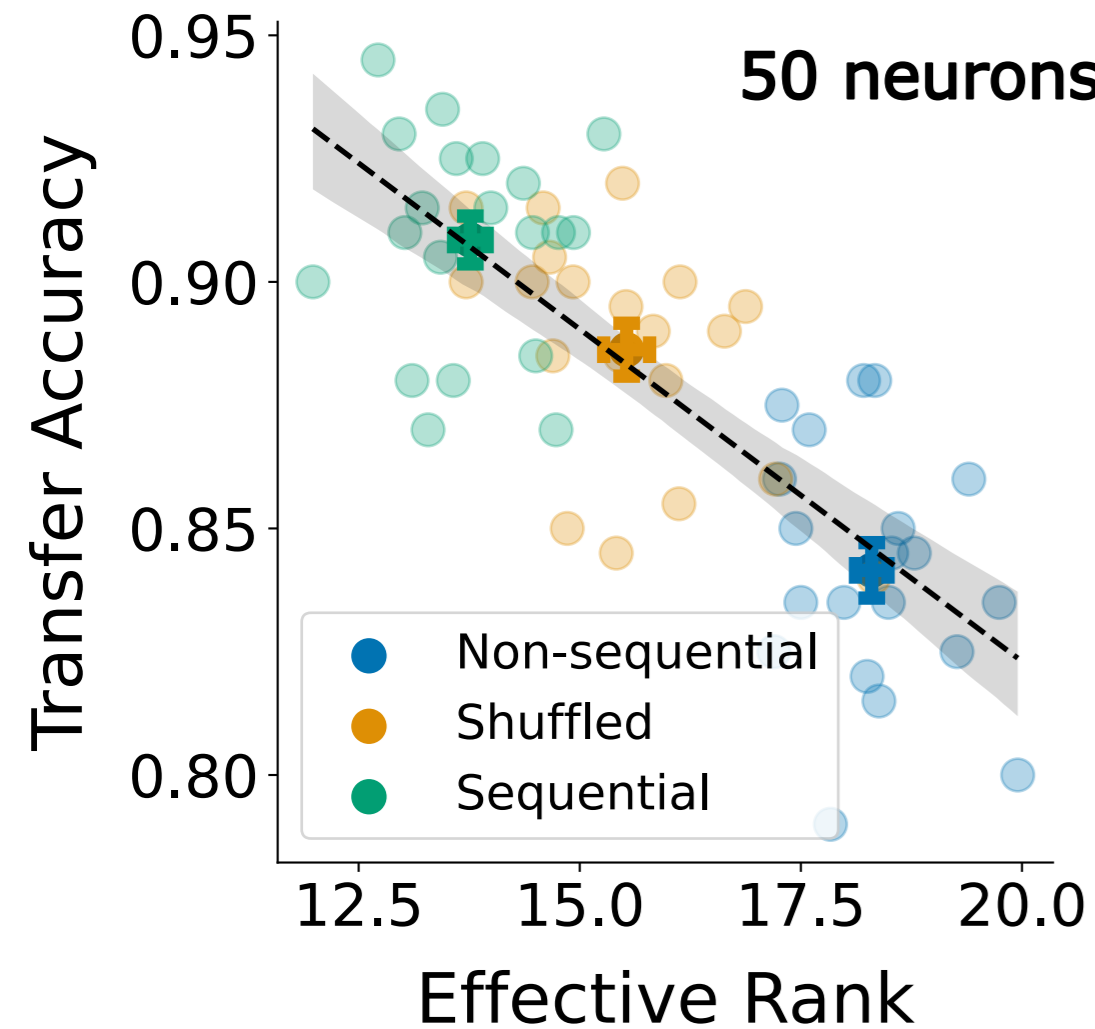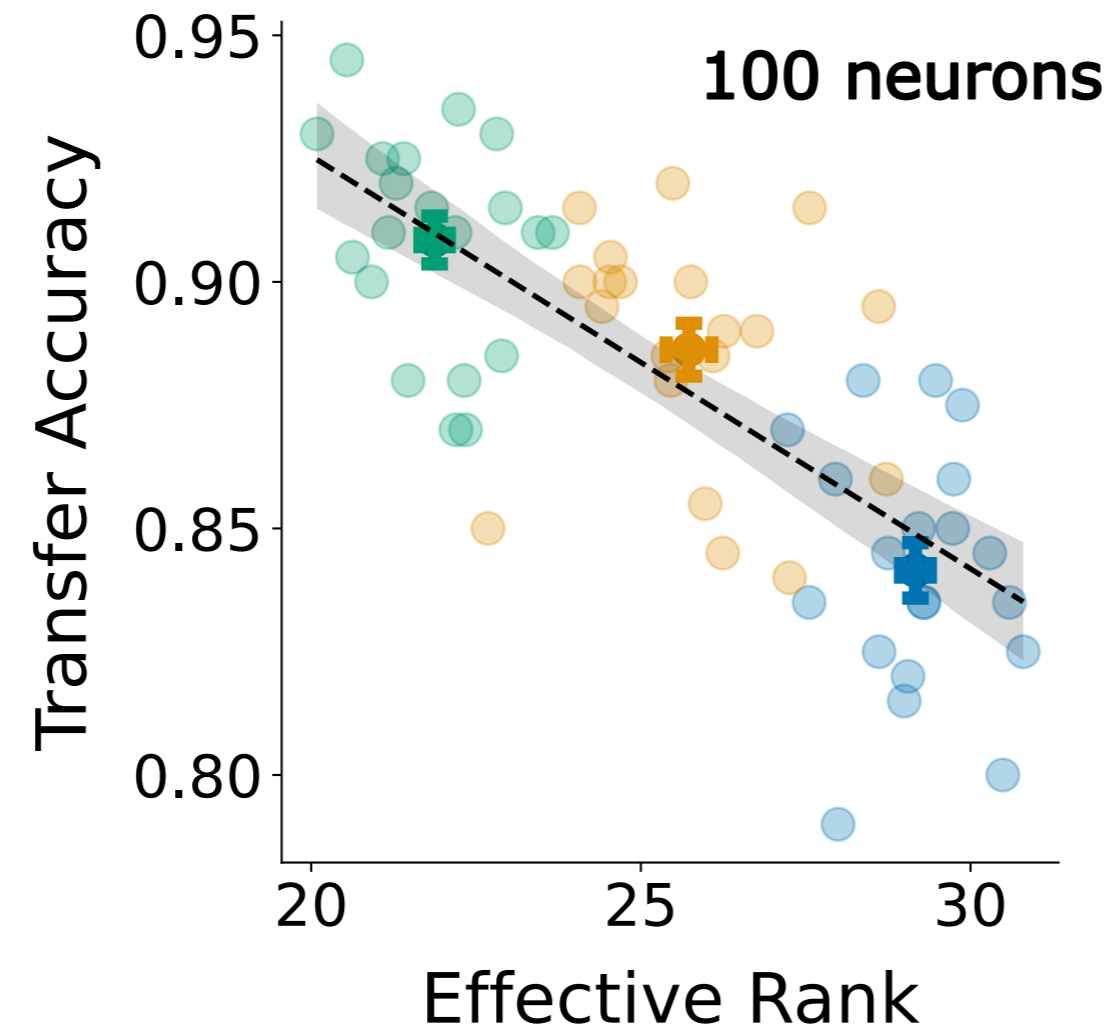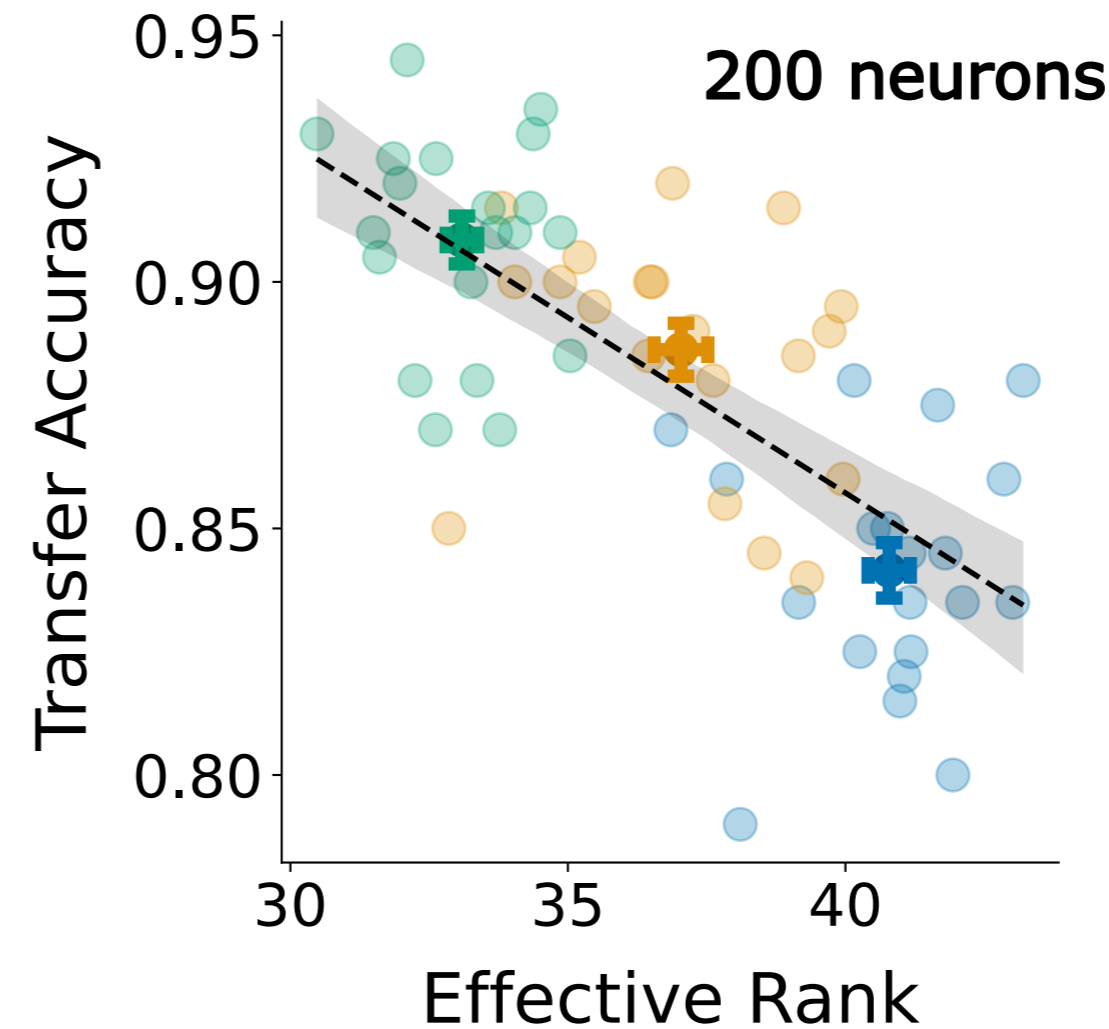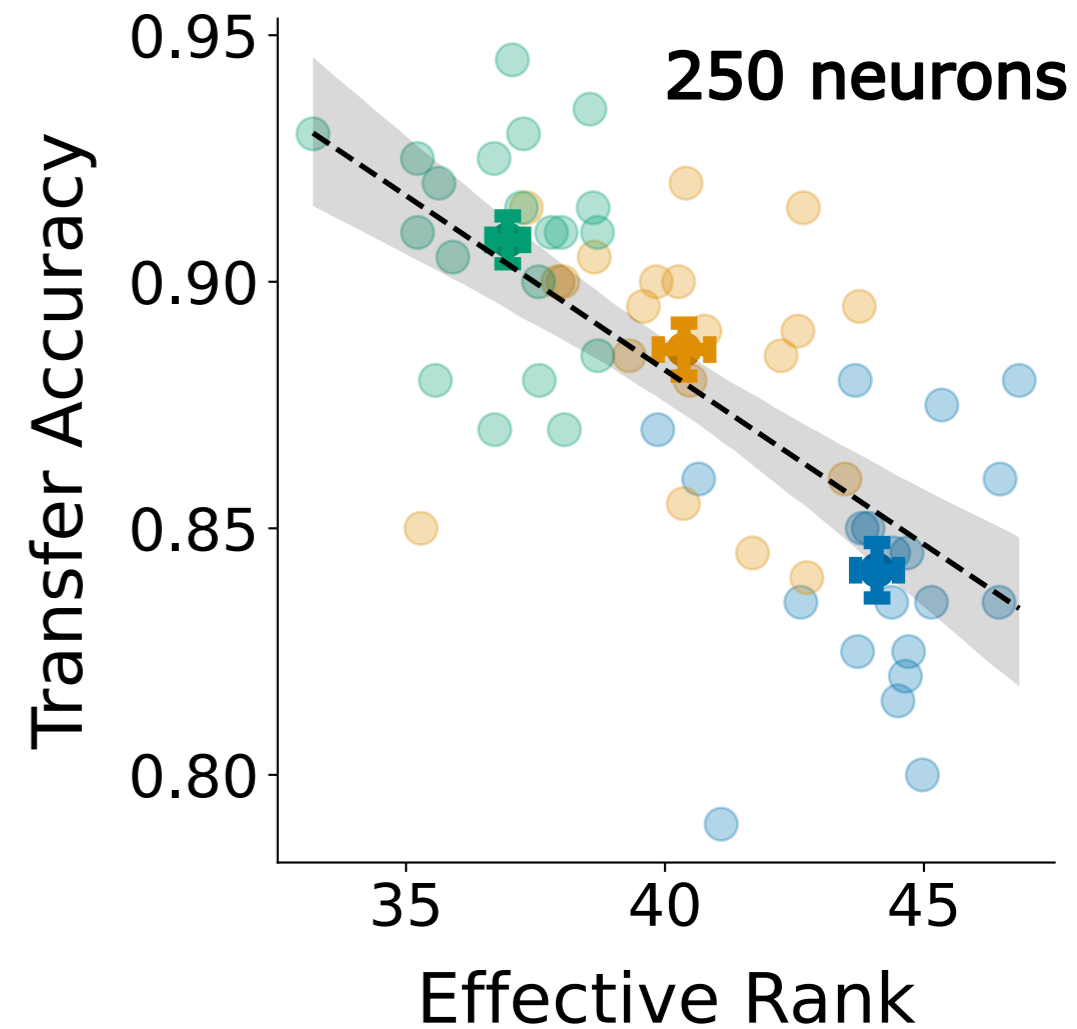

Supplement: S5 Fig — Transfer accuracy versus effective rank for different subspace sizes (from left to right: 50 (r = -0.78, p<<0.0001), 100 (r = -0.74, p<<0.0001), 200 (r = -0.70, p<<0.0001), and 250 (r = -0.67, p<<0.0001) neurons). (PDF) [file pcbi.1014553.s005.pdf]

A

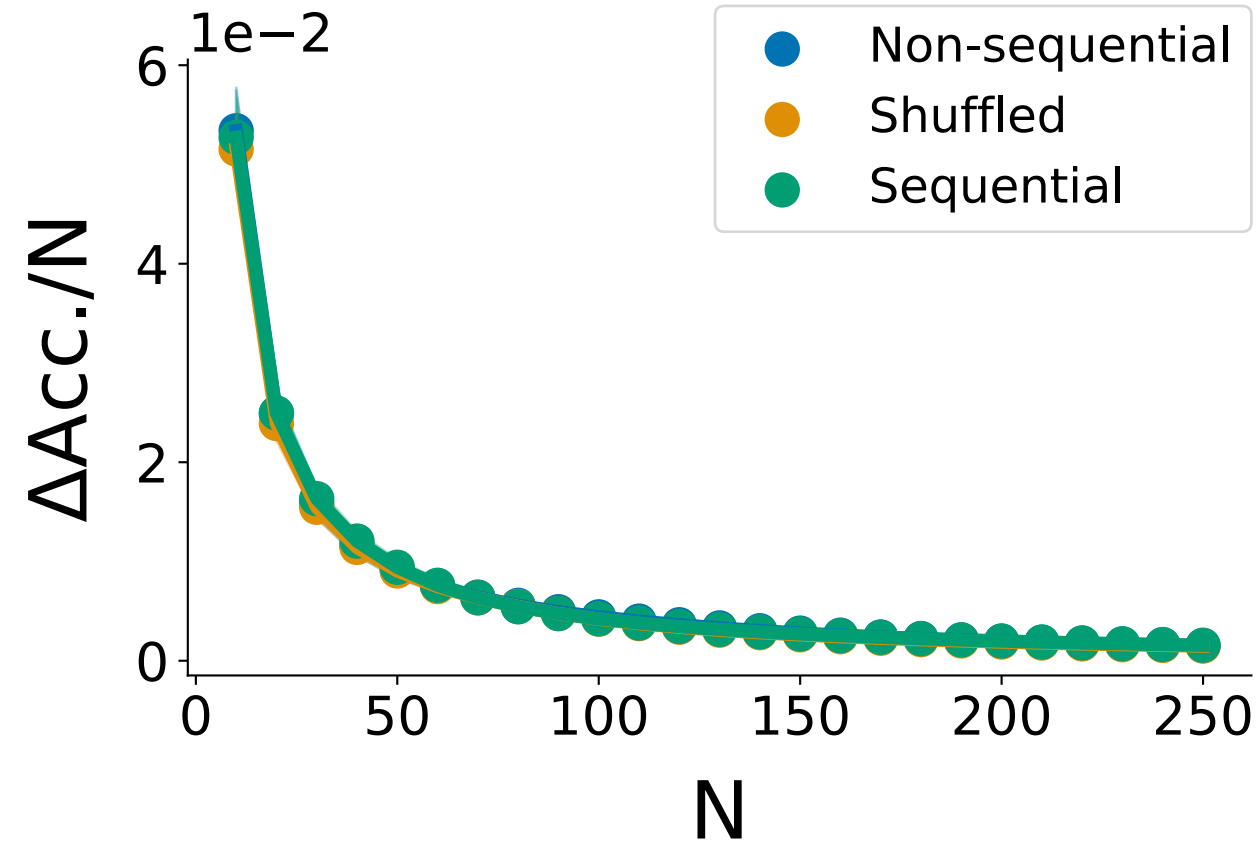

B

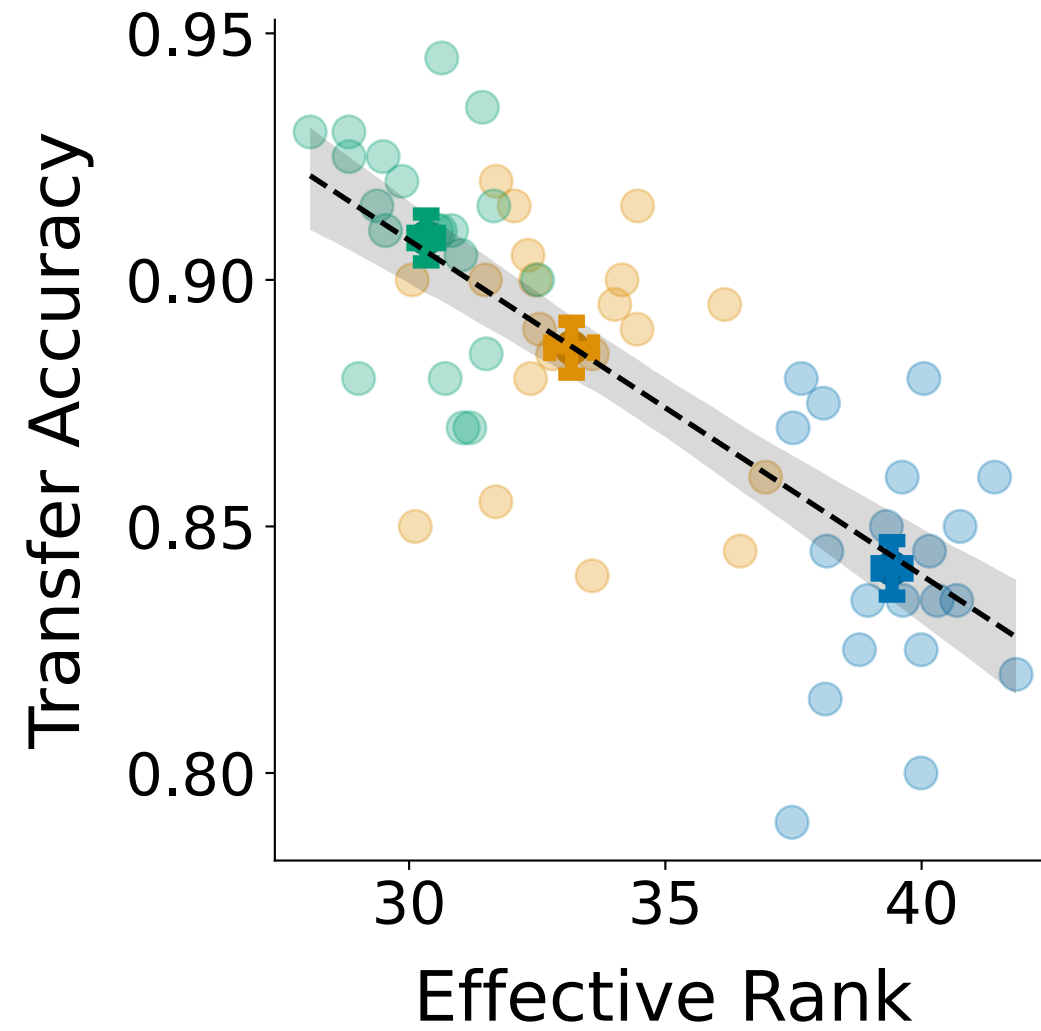

C

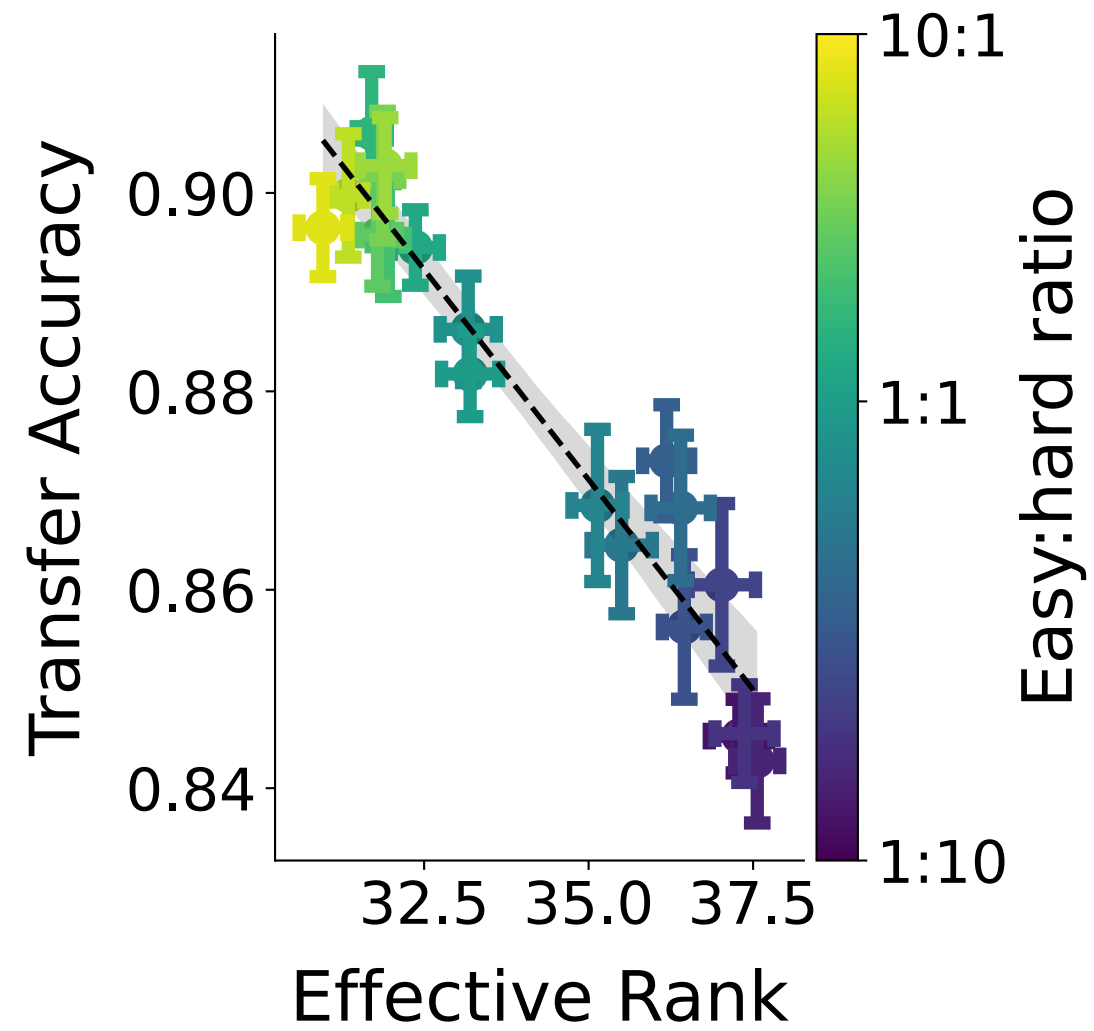

Supplement: S6 Fig — (A) Accuracy drop per neuron lesioned, plotted against the number of neurons lesioned. The neurons were chosen by their functional contribution to the transfer accuracy. The accuracy drop per neuron plateaued at about N = 150, meaning that the readout subspace was mostly affected by about 150 neurons. (B) Transfer accuracy versus effective rank for models across curricula (r = -0.76, p<<0.0001). (C) Negative correlation of the effective rank for shuffled curricula with different easy:hard sample ratios with transfer accuracy (r = -0.96, p<<0.0001). (PDF) [file pcbi.1014553.s006.pdf]

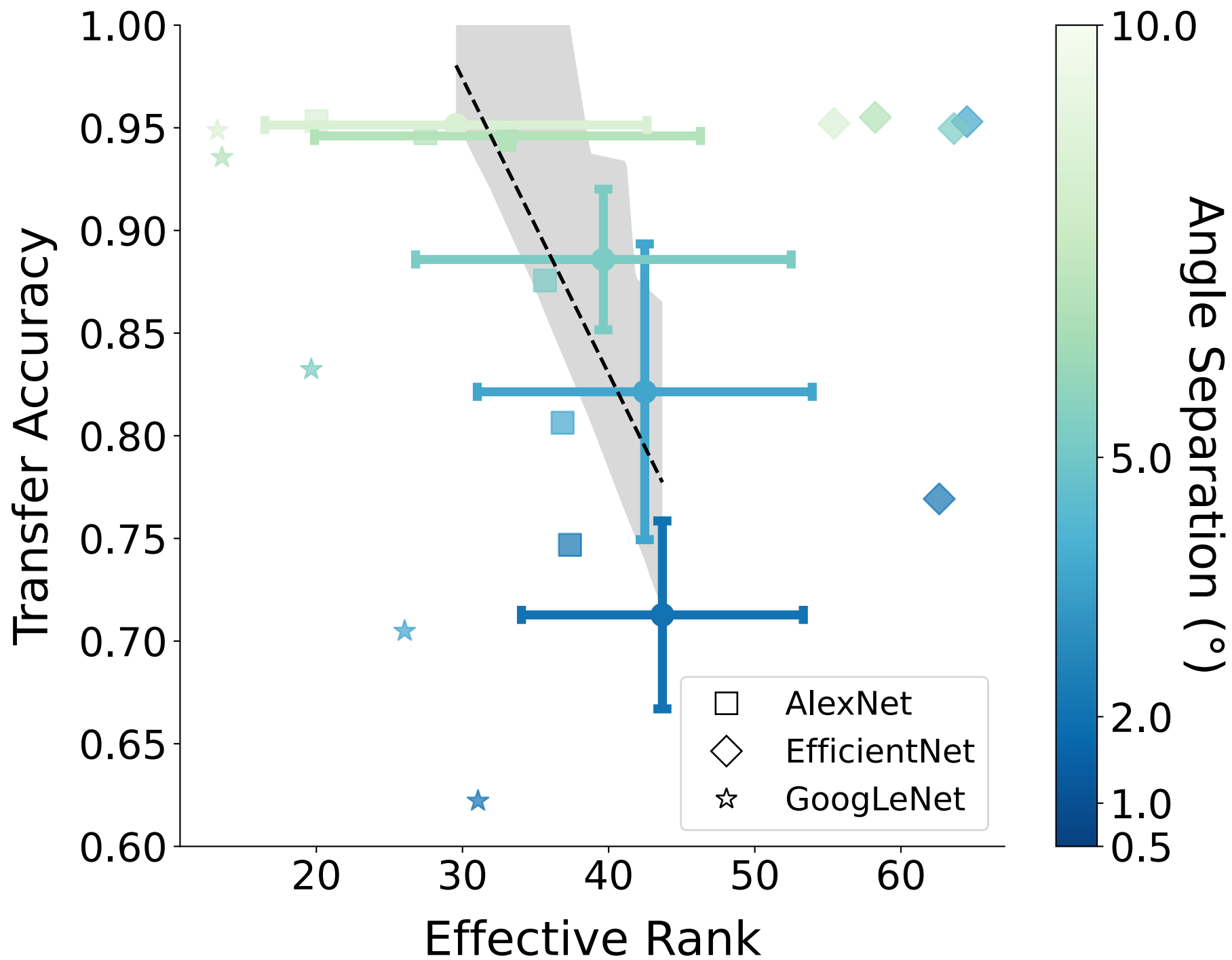

Supplement: S7 Fig — Transfer accuracy versus effective rank for selected model backbones (AlexNet, EfficientNet, and GoogLeNet) trained and tested on single angle separations. Correlation is calculated based on the means across all models for single angle separation (r = -0.89, p = 0.046). (PDF) [file pcbi.1014553.s007.pdf]

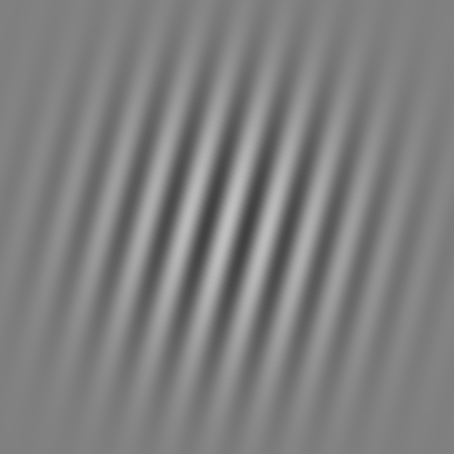

Supplement: S2 Data — (ZIP) [file pcbi.1014553.s010.zip › Curriculum-VPL-main/model_code/StimulusImages/SG_refs/REFERENCE_ref_15_sep_0.0_contr_1_ph_0.0_sf_0.05_NONE.png]

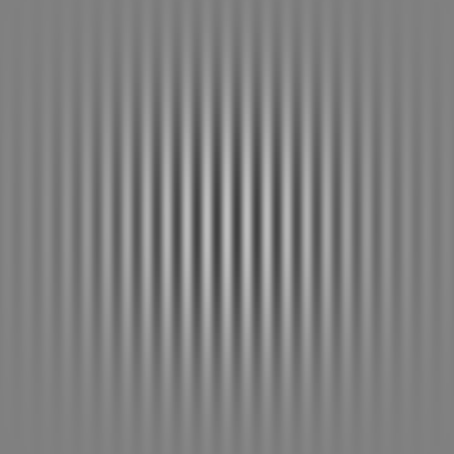

Supplement: S2 Data — (ZIP) [file pcbi.1014553.s010.zip › Curriculum-VPL-main/model_code/StimulusImages/SG_refs/REFERENCE_ref_0_sep_0.0_contr_1_ph_0.0_sf_0.1_NONE.png]

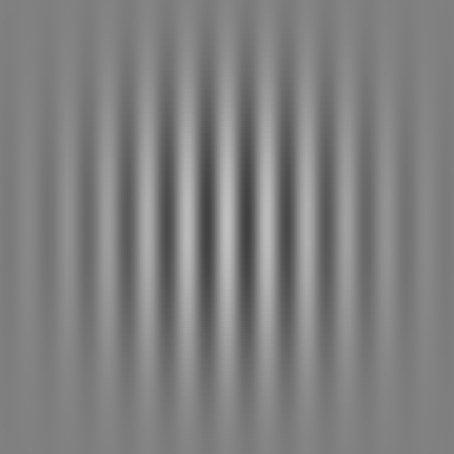

Supplement: S2 Data — (ZIP) [file pcbi.1014553.s010.zip › Curriculum-VPL-main/model_code/StimulusImages/SG_refs/REFERENCE_ref_0_sep_0.0_contr_1_ph_0.0_sf_0.05_NONE.png]

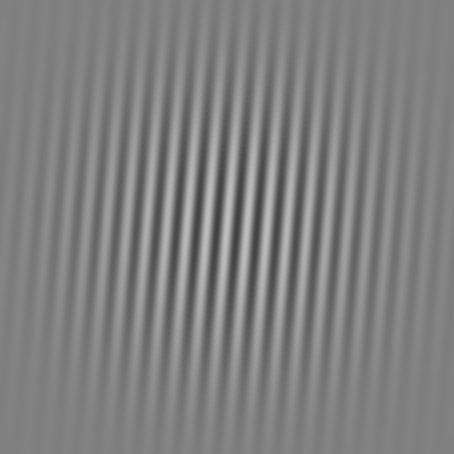

Supplement: S2 Data — (ZIP) [file pcbi.1014553.s010.zip › Curriculum-VPL-main/model_code/StimulusImages/SG_test_double_sf/sep_5.0/TARGET_ref_0_sep_5.0_contr_1_ph_0.0_sf_0.1_CW.png]

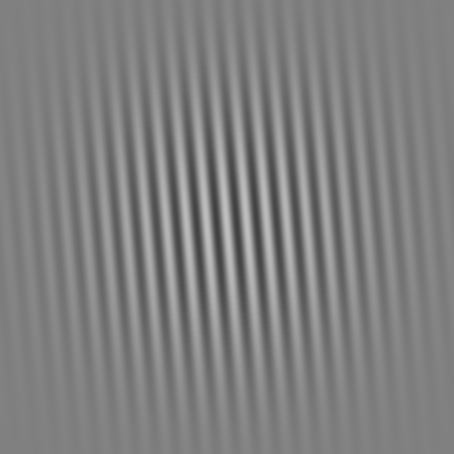

Supplement: S2 Data — (ZIP) [file pcbi.1014553.s010.zip › Curriculum-VPL-main/model_code/StimulusImages/SG_test_double_sf/sep_5.0/TARGET_ref_0_sep_5.0_contr_1_ph_0.0_sf_0.1_CCW.png]

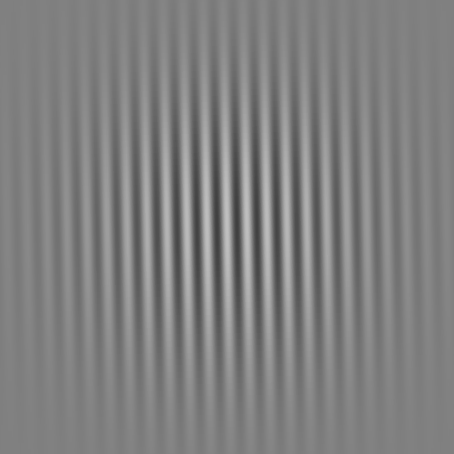

Supplement: S2 Data — (ZIP) [file pcbi.1014553.s010.zip › Curriculum-VPL-main/model_code/StimulusImages/SG_test_double_sf/sep_1.0/TARGET_ref_0_sep_1.0_contr_1_ph_0.0_sf_0.1_CCW.png]

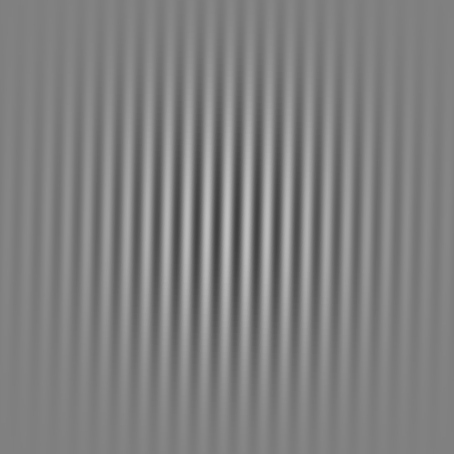

Supplement: S2 Data — (ZIP) [file pcbi.1014553.s010.zip › Curriculum-VPL-main/model_code/StimulusImages/SG_test_double_sf/sep_1.0/TARGET_ref_0_sep_1.0_contr_1_ph_0.0_sf_0.1_CW.png]

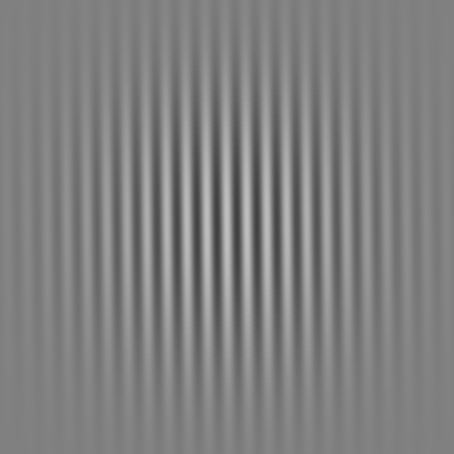

Supplement: S2 Data — (ZIP) [file pcbi.1014553.s010.zip › Curriculum-VPL-main/model_code/StimulusImages/SG_test_double_sf/sep_0.5/TARGET_ref_0_sep_0.5_contr_1_ph_0.0_sf_0.1_CCW.png]

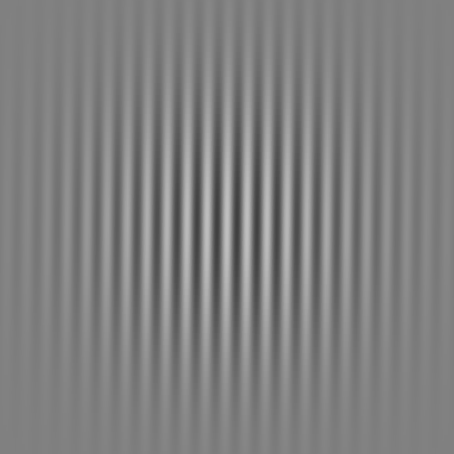

Supplement: S2 Data — (ZIP) [file pcbi.1014553.s010.zip › Curriculum-VPL-main/model_code/StimulusImages/SG_test_double_sf/sep_0.5/TARGET_ref_0_sep_0.5_contr_1_ph_0.0_sf_0.1_CW.png]

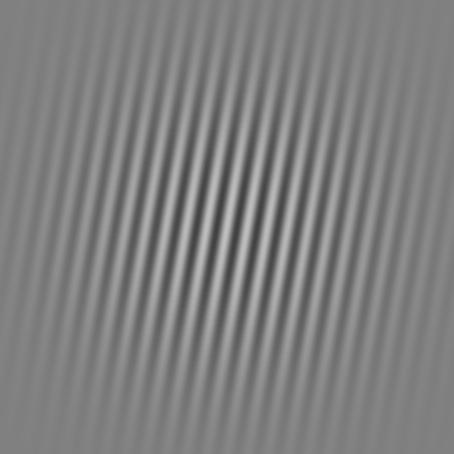

Supplement: S2 Data — (ZIP) [file pcbi.1014553.s010.zip › Curriculum-VPL-main/model_code/StimulusImages/SG_test_double_sf/sep_10.0/TARGET_ref_0_sep_10.0_contr_1_ph_0.0_sf_0.1_CW.png]

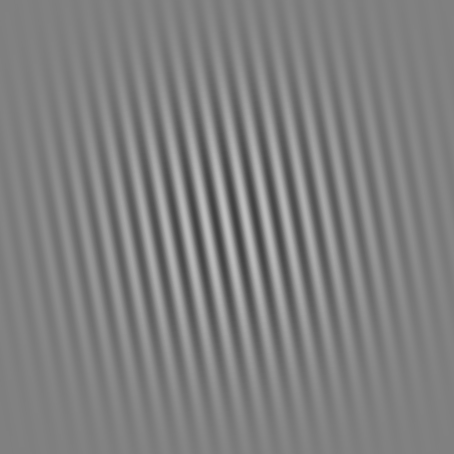

Supplement: S2 Data — (ZIP) [file pcbi.1014553.s010.zip › Curriculum-VPL-main/model_code/StimulusImages/SG_test_double_sf/sep_10.0/TARGET_ref_0_sep_10.0_contr_1_ph_0.0_sf_0.1_CCW.png]

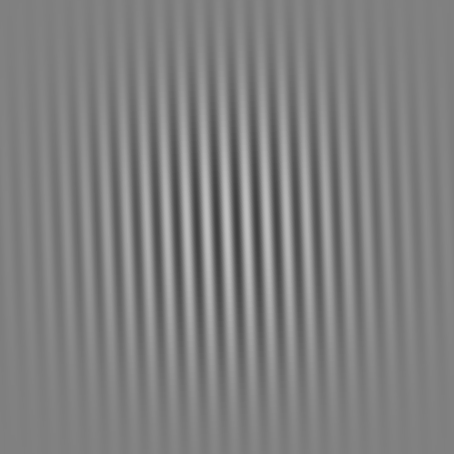

Supplement: S2 Data — (ZIP) [file pcbi.1014553.s010.zip › Curriculum-VPL-main/model_code/StimulusImages/SG_test_double_sf/sep_2.0/TARGET_ref_0_sep_2.0_contr_1_ph_0.0_sf_0.1_CCW.png]

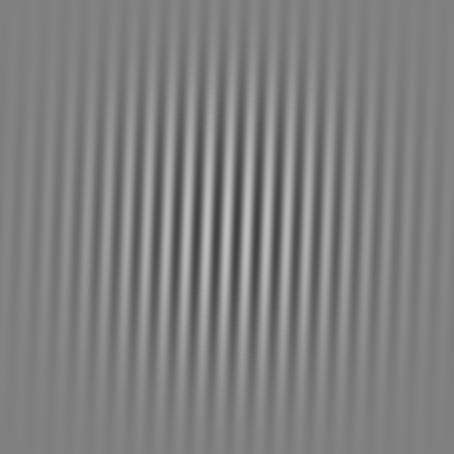

Supplement: S2 Data — (ZIP) [file pcbi.1014553.s010.zip › Curriculum-VPL-main/model_code/StimulusImages/SG_test_double_sf/sep_2.0/TARGET_ref_0_sep_2.0_contr_1_ph_0.0_sf_0.1_CW.png]

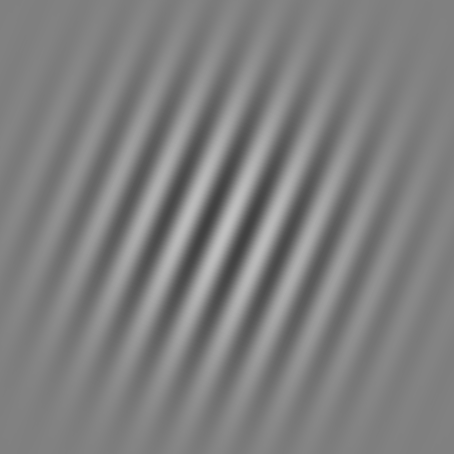

Supplement: S2 Data — (ZIP) [file pcbi.1014553.s010.zip › Curriculum-VPL-main/model_code/StimulusImages/SG_train_double_sf/sep_157.0/TARGET_ref_0_sep_157.0_contr_1_ph_0.0_sf_0.05_CCW.png]

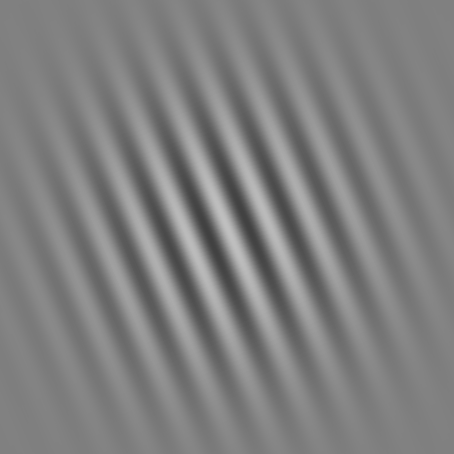

Supplement: S2 Data — (ZIP) [file pcbi.1014553.s010.zip › Curriculum-VPL-main/model_code/StimulusImages/SG_train_double_sf/sep_157.0/TARGET_ref_0_sep_157.0_contr_1_ph_0.0_sf_0.05_CW.png]

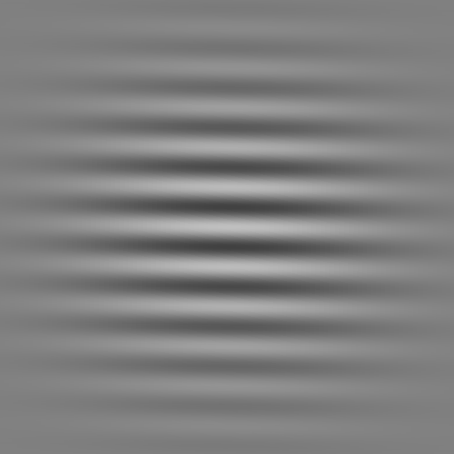

Supplement: S2 Data — (ZIP) [file pcbi.1014553.s010.zip › Curriculum-VPL-main/model_code/StimulusImages/SG_train_double_sf/sep_91.0/TARGET_ref_0_sep_91.0_contr_1_ph_0.0_sf_0.05_CW.png]

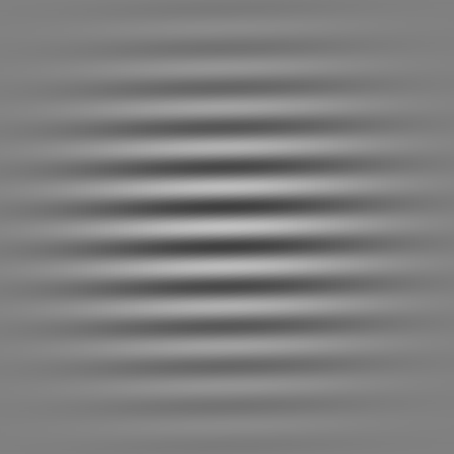

Supplement: S2 Data — (ZIP) [file pcbi.1014553.s010.zip › Curriculum-VPL-main/model_code/StimulusImages/SG_train_double_sf/sep_91.0/TARGET_ref_0_sep_91.0_contr_1_ph_0.0_sf_0.05_CCW.png]

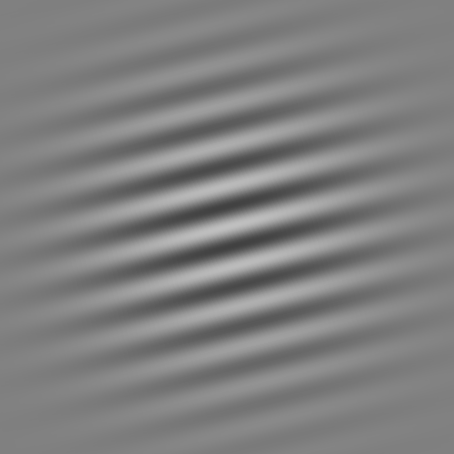

Supplement: S2 Data — (ZIP) [file pcbi.1014553.s010.zip › Curriculum-VPL-main/model_code/StimulusImages/SG_train_double_sf/sep_76.0/TARGET_ref_0_sep_76.0_contr_1_ph_0.0_sf_0.05_CW.png]

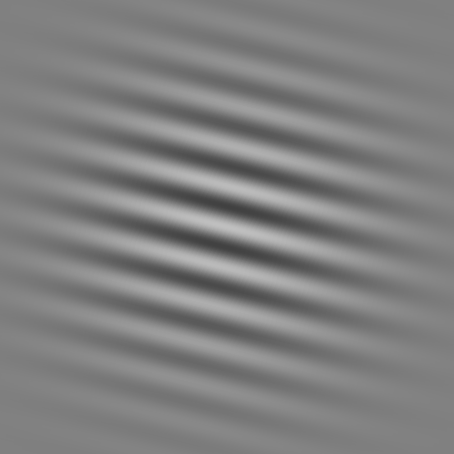

Supplement: S2 Data — (ZIP) [file pcbi.1014553.s010.zip › Curriculum-VPL-main/model_code/StimulusImages/SG_train_double_sf/sep_76.0/TARGET_ref_0_sep_76.0_contr_1_ph_0.0_sf_0.05_CCW.png]

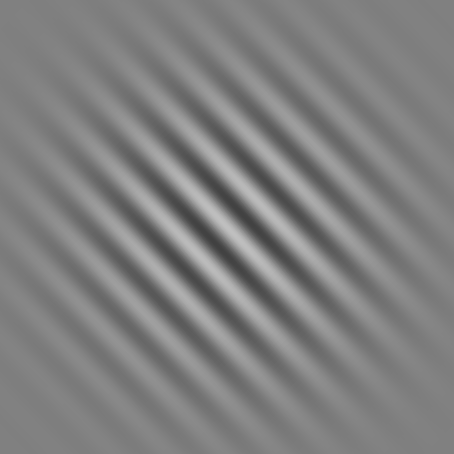

Supplement: S2 Data — (ZIP) [file pcbi.1014553.s010.zip › Curriculum-VPL-main/model_code/StimulusImages/SG_train_double_sf/sep_136.0/TARGET_ref_0_sep_136.0_contr_1_ph_0.0_sf_0.05_CW.png]

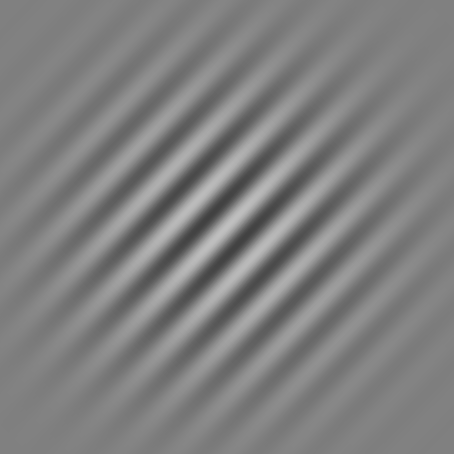

Supplement: S2 Data — (ZIP) [file pcbi.1014553.s010.zip › Curriculum-VPL-main/model_code/StimulusImages/SG_train_double_sf/sep_136.0/TARGET_ref_0_sep_136.0_contr_1_ph_0.0_sf_0.05_CCW.png]

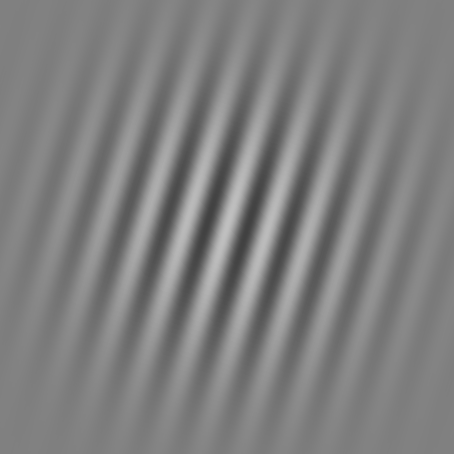

Supplement: S2 Data — (ZIP) [file pcbi.1014553.s010.zip › Curriculum-VPL-main/model_code/StimulusImages/SG_train_double_sf/sep_17.0/TARGET_ref_0_sep_17.0_contr_1_ph_0.0_sf_0.05_CW.png]

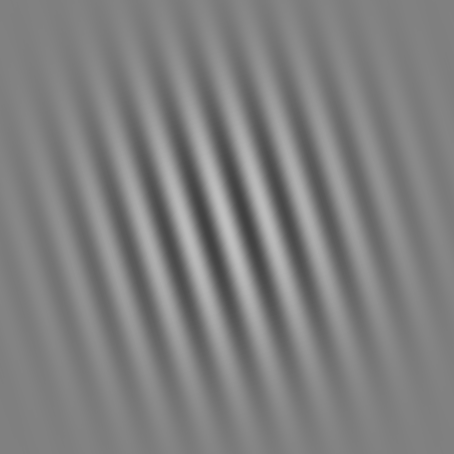

Supplement: S2 Data — (ZIP) [file pcbi.1014553.s010.zip › Curriculum-VPL-main/model_code/StimulusImages/SG_train_double_sf/sep_17.0/TARGET_ref_0_sep_17.0_contr_1_ph_0.0_sf_0.05_CCW.png]

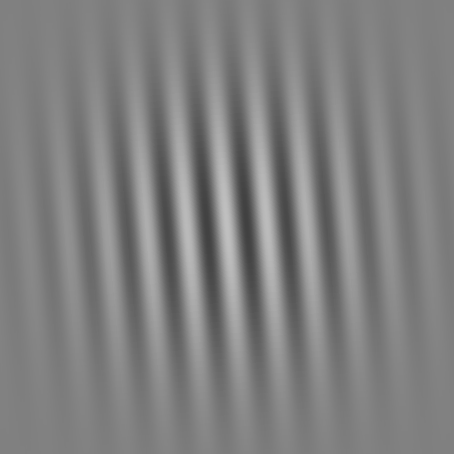

Supplement: S2 Data — (ZIP) [file pcbi.1014553.s010.zip › Curriculum-VPL-main/model_code/StimulusImages/SG_train_double_sf/sep_5.0/TARGET_ref_0_sep_5.0_contr_1_ph_0.0_sf_0.05_CCW.png]

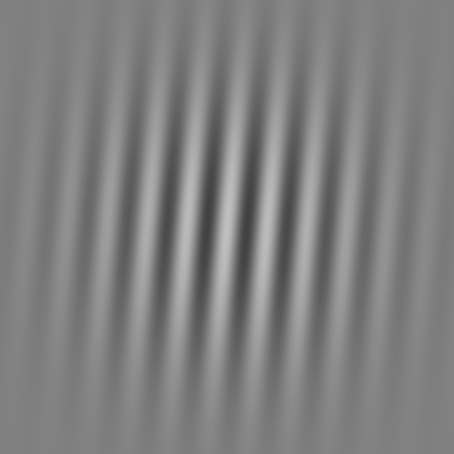

Supplement: S2 Data — (ZIP) [file pcbi.1014553.s010.zip › Curriculum-VPL-main/model_code/StimulusImages/SG_train_double_sf/sep_5.0/TARGET_ref_0_sep_5.0_contr_1_ph_0.0_sf_0.05_CW.png]

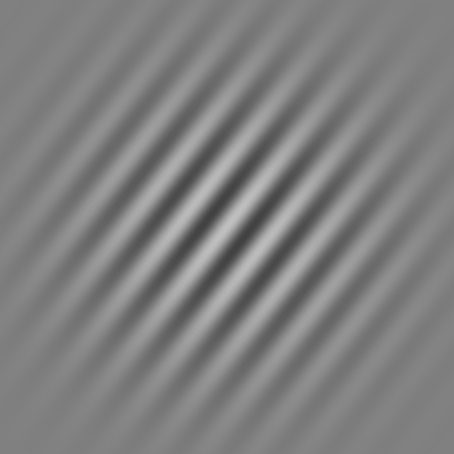

Supplement: S2 Data — (ZIP) [file pcbi.1014553.s010.zip › Curriculum-VPL-main/model_code/StimulusImages/SG_train_double_sf/sep_38.0/TARGET_ref_0_sep_38.0_contr_1_ph_0.0_sf_0.05_CW.png]

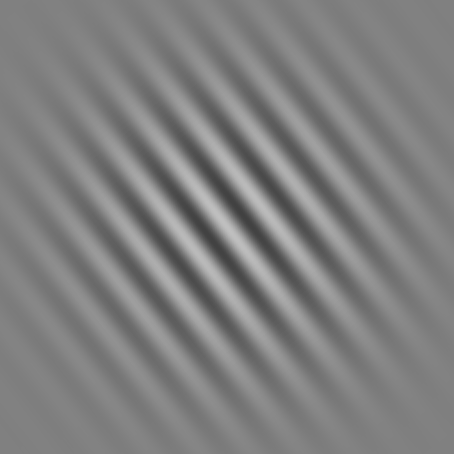

Supplement: S2 Data — (ZIP) [file pcbi.1014553.s010.zip › Curriculum-VPL-main/model_code/StimulusImages/SG_train_double_sf/sep_38.0/TARGET_ref_0_sep_38.0_contr_1_ph_0.0_sf_0.05_CCW.png]

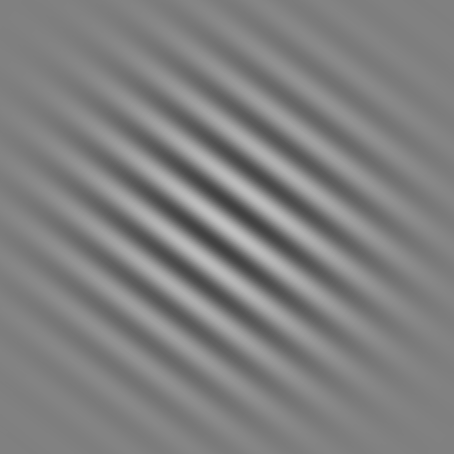

Supplement: S2 Data — (ZIP) [file pcbi.1014553.s010.zip › Curriculum-VPL-main/model_code/StimulusImages/SG_train_double_sf/sep_53.0/TARGET_ref_0_sep_53.0_contr_1_ph_0.0_sf_0.05_CCW.png]

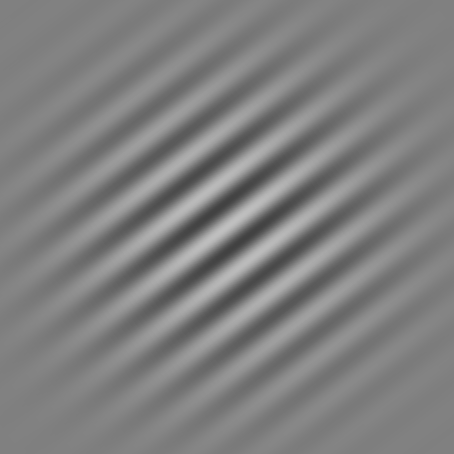

Supplement: S2 Data — (ZIP) [file pcbi.1014553.s010.zip › Curriculum-VPL-main/model_code/StimulusImages/SG_train_double_sf/sep_53.0/TARGET_ref_0_sep_53.0_contr_1_ph_0.0_sf_0.05_CW.png]

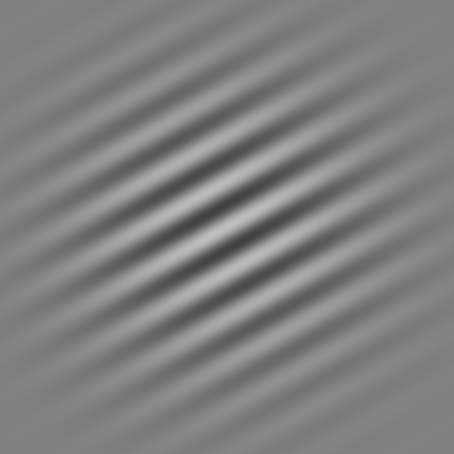

Supplement: S2 Data — (ZIP) [file pcbi.1014553.s010.zip › Curriculum-VPL-main/model_code/StimulusImages/SG_train_double_sf/sep_119.0/TARGET_ref_0_sep_119.0_contr_1_ph_0.0_sf_0.05_CCW.png]

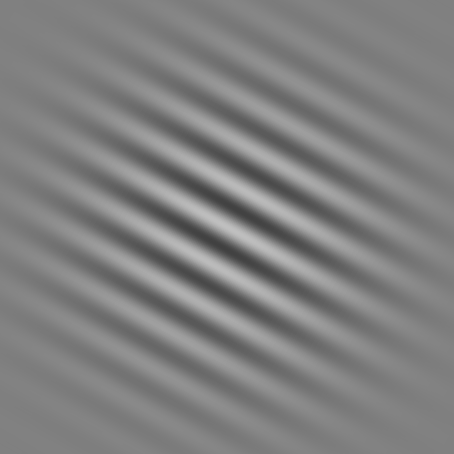

Supplement: S2 Data — (ZIP) [file pcbi.1014553.s010.zip › Curriculum-VPL-main/model_code/StimulusImages/SG_train_double_sf/sep_119.0/TARGET_ref_0_sep_119.0_contr_1_ph_0.0_sf_0.05_CW.png]

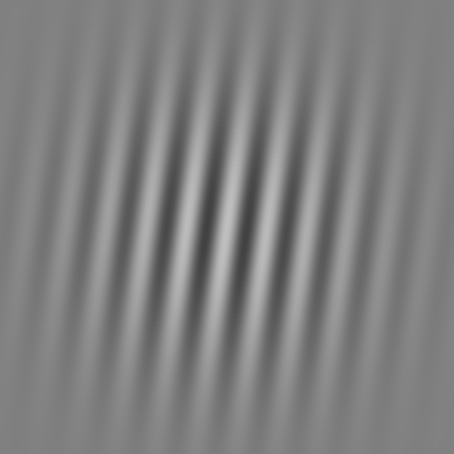

Supplement: S2 Data — (ZIP) [file pcbi.1014553.s010.zip › Curriculum-VPL-main/model_code/StimulusImages/SG_train_double_sf/sep_172.0/TARGET_ref_0_sep_172.0_contr_1_ph_0.0_sf_0.05_CCW.png]

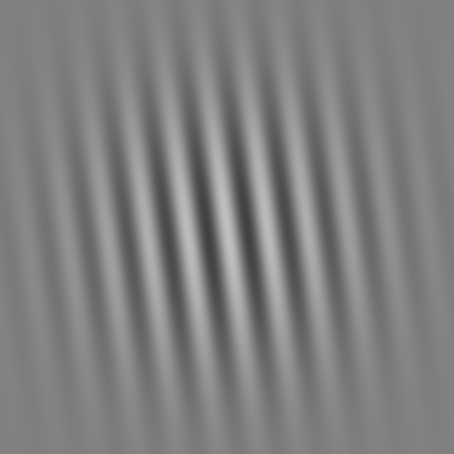

Supplement: S2 Data — (ZIP) [file pcbi.1014553.s010.zip › Curriculum-VPL-main/model_code/StimulusImages/SG_train_double_sf/sep_172.0/TARGET_ref_0_sep_172.0_contr_1_ph_0.0_sf_0.05_CW.png]

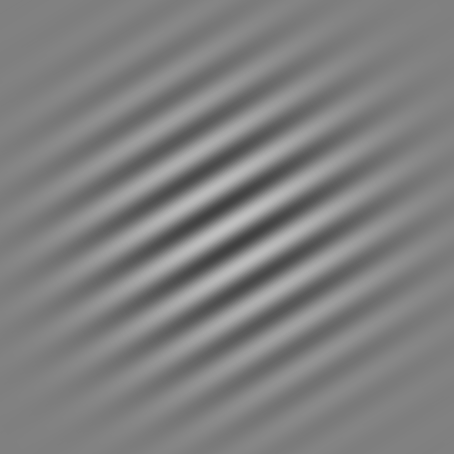

Supplement: S2 Data — (ZIP) [file pcbi.1014553.s010.zip › Curriculum-VPL-main/model_code/StimulusImages/SG_train_double_sf/sep_59.0/TARGET_ref_0_sep_59.0_contr_1_ph_0.0_sf_0.05_CW.png]

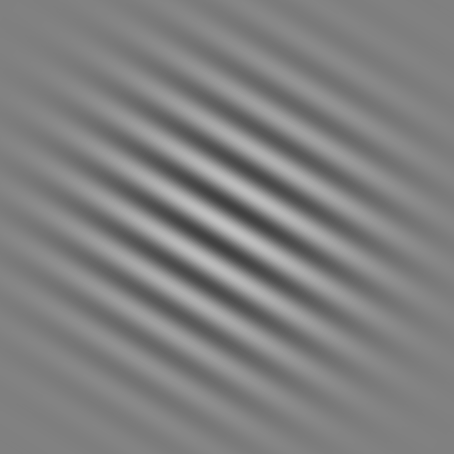

Supplement: S2 Data — (ZIP) [file pcbi.1014553.s010.zip › Curriculum-VPL-main/model_code/StimulusImages/SG_train_double_sf/sep_59.0/TARGET_ref_0_sep_59.0_contr_1_ph_0.0_sf_0.05_CCW.png]

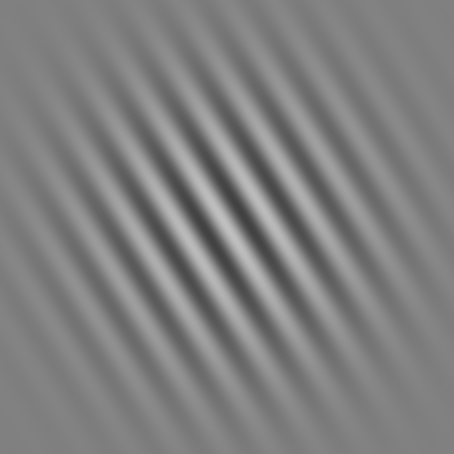

Supplement: S2 Data — (ZIP) [file pcbi.1014553.s010.zip › Curriculum-VPL-main/model_code/StimulusImages/SG_train_double_sf/sep_32.0/TARGET_ref_0_sep_32.0_contr_1_ph_0.0_sf_0.05_CCW.png]

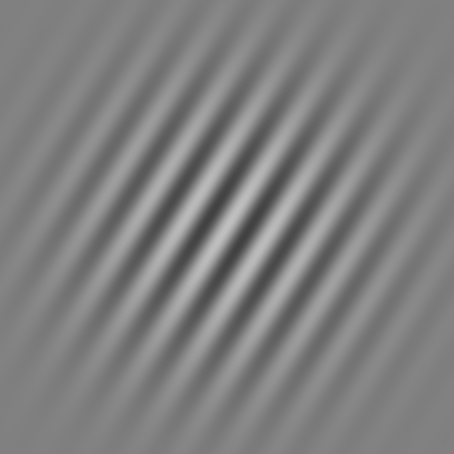

Supplement: S2 Data — (ZIP) [file pcbi.1014553.s010.zip › Curriculum-VPL-main/model_code/StimulusImages/SG_train_double_sf/sep_32.0/TARGET_ref_0_sep_32.0_contr_1_ph_0.0_sf_0.05_CW.png]

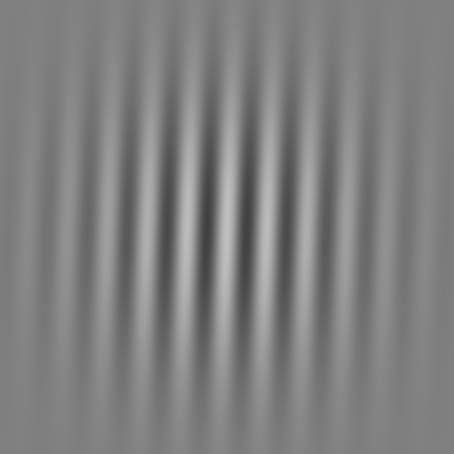

Supplement: S2 Data — (ZIP) [file pcbi.1014553.s010.zip › Curriculum-VPL-main/model_code/StimulusImages/SG_train_double_sf/sep_178.0/TARGET_ref_0_sep_178.0_contr_1_ph_0.0_sf_0.05_CCW.png]

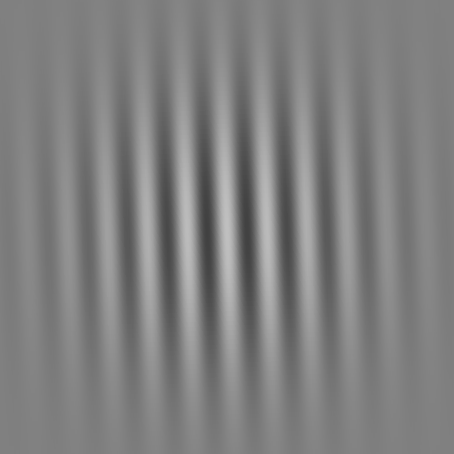

Supplement: S2 Data — (ZIP) [file pcbi.1014553.s010.zip › Curriculum-VPL-main/model_code/StimulusImages/SG_train_double_sf/sep_178.0/TARGET_ref_0_sep_178.0_contr_1_ph_0.0_sf_0.05_CW.png]

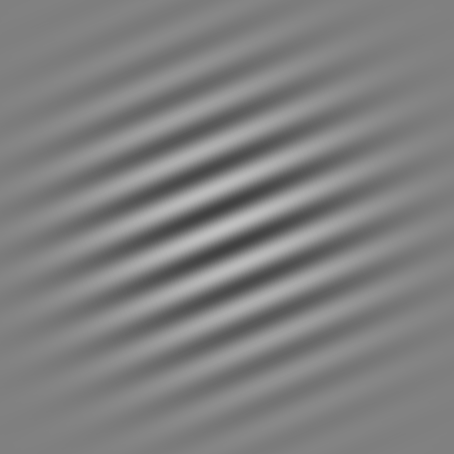

Supplement: S2 Data — (ZIP) [file pcbi.1014553.s010.zip › Curriculum-VPL-main/model_code/StimulusImages/SG_train_double_sf/sep_113.0/TARGET_ref_0_sep_113.0_contr_1_ph_0.0_sf_0.05_CCW.png]

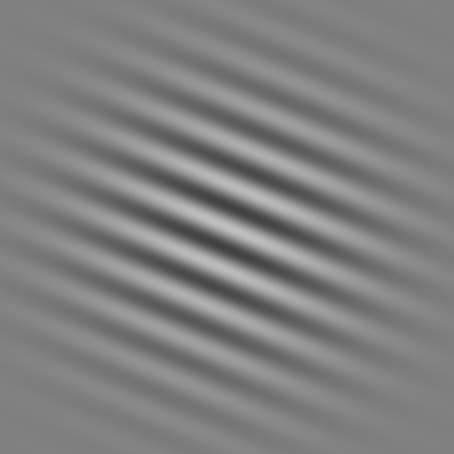

Supplement: S2 Data — (ZIP) [file pcbi.1014553.s010.zip › Curriculum-VPL-main/model_code/StimulusImages/SG_train_double_sf/sep_113.0/TARGET_ref_0_sep_113.0_contr_1_ph_0.0_sf_0.05_CW.png]

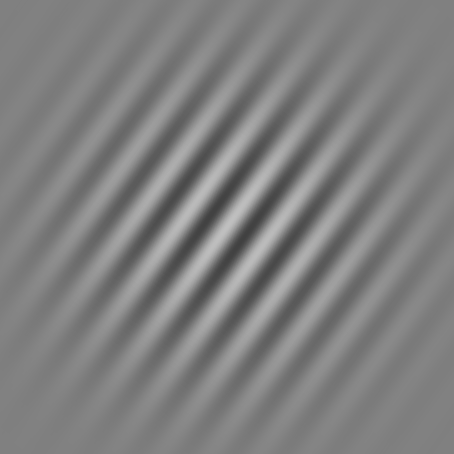

Supplement: S2 Data — (ZIP) [file pcbi.1014553.s010.zip › Curriculum-VPL-main/model_code/StimulusImages/SG_train_double_sf/sep_36.0/TARGET_ref_0_sep_36.0_contr_1_ph_0.0_sf_0.05_CW.png]

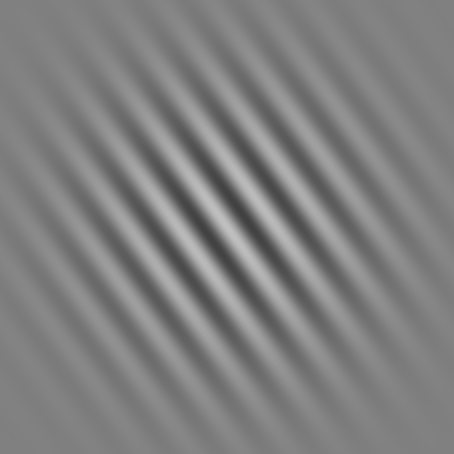

Supplement: S2 Data — (ZIP) [file pcbi.1014553.s010.zip › Curriculum-VPL-main/model_code/StimulusImages/SG_train_double_sf/sep_36.0/TARGET_ref_0_sep_36.0_contr_1_ph_0.0_sf_0.05_CCW.png]

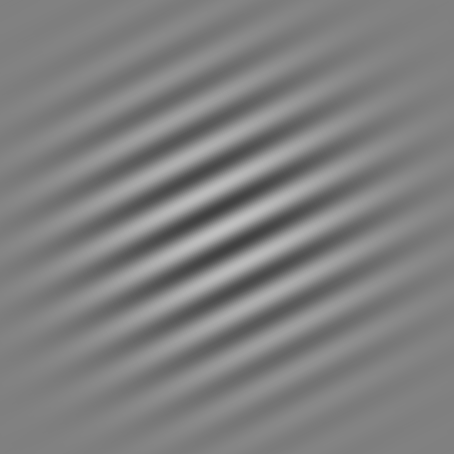

Supplement: S2 Data — (ZIP) [file pcbi.1014553.s010.zip › Curriculum-VPL-main/model_code/StimulusImages/SG_train_double_sf/sep_117.0/TARGET_ref_0_sep_117.0_contr_1_ph_0.0_sf_0.05_CCW.png]

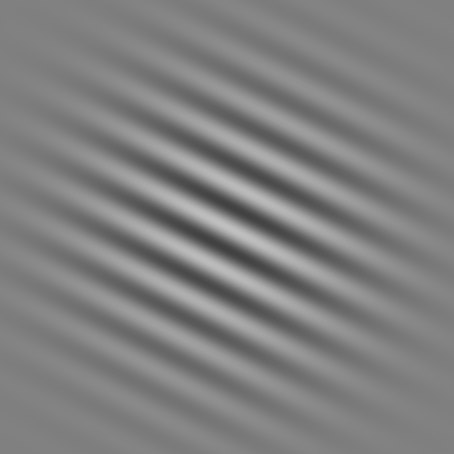

Supplement: S2 Data — (ZIP) [file pcbi.1014553.s010.zip › Curriculum-VPL-main/model_code/StimulusImages/SG_train_double_sf/sep_117.0/TARGET_ref_0_sep_117.0_contr_1_ph_0.0_sf_0.05_CW.png]

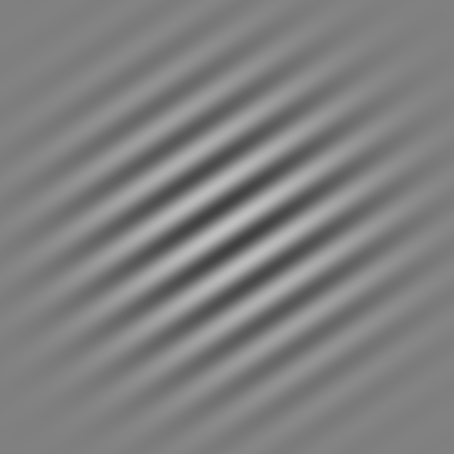

Supplement: S2 Data — (ZIP) [file pcbi.1014553.s010.zip › Curriculum-VPL-main/model_code/StimulusImages/SG_train_double_sf/sep_57.0/TARGET_ref_0_sep_57.0_contr_1_ph_0.0_sf_0.05_CW.png]

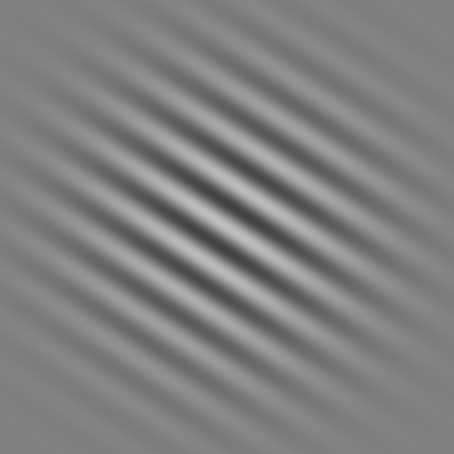

Supplement: S2 Data — (ZIP) [file pcbi.1014553.s010.zip › Curriculum-VPL-main/model_code/StimulusImages/SG_train_double_sf/sep_57.0/TARGET_ref_0_sep_57.0_contr_1_ph_0.0_sf_0.05_CCW.png]

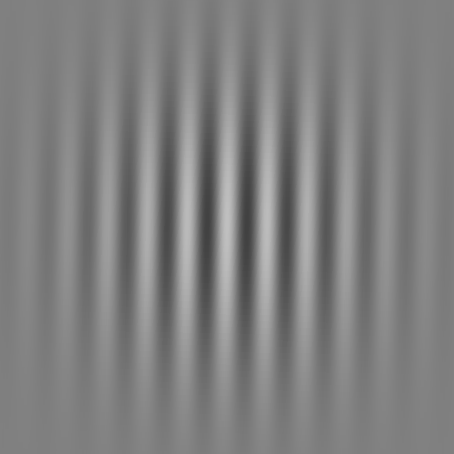

Supplement: S2 Data — (ZIP) [file pcbi.1014553.s010.zip › Curriculum-VPL-main/model_code/StimulusImages/SG_train_double_sf/sep_1.0/TARGET_ref_0_sep_1.0_contr_1_ph_0.0_sf_0.05_CW.png]

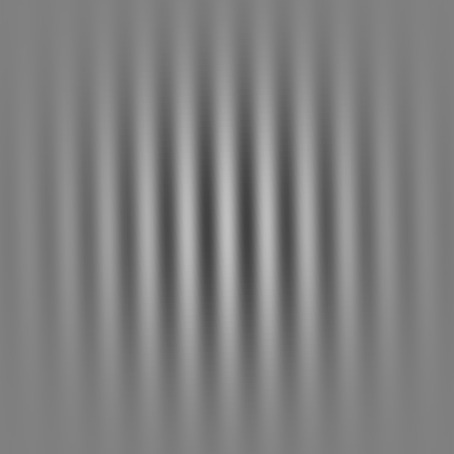

Supplement: S2 Data — (ZIP) [file pcbi.1014553.s010.zip › Curriculum-VPL-main/model_code/StimulusImages/SG_train_double_sf/sep_1.0/TARGET_ref_0_sep_1.0_contr_1_ph_0.0_sf_0.05_CCW.png]

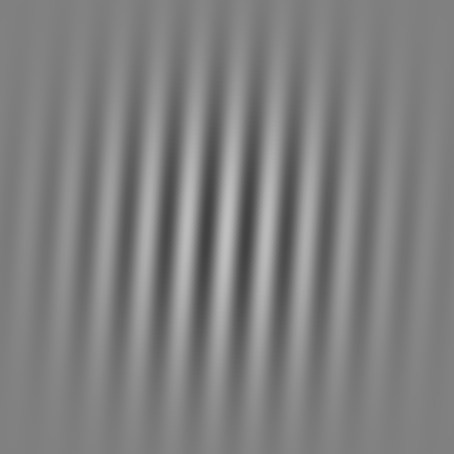

Supplement: S2 Data — (ZIP) [file pcbi.1014553.s010.zip › Curriculum-VPL-main/model_code/StimulusImages/SG_train_double_sf/sep_176.0/TARGET_ref_0_sep_176.0_contr_1_ph_0.0_sf_0.05_CCW.png]

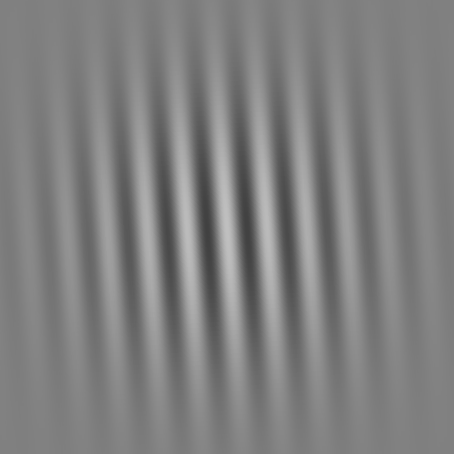

Supplement: S2 Data — (ZIP) [file pcbi.1014553.s010.zip › Curriculum-VPL-main/model_code/StimulusImages/SG_train_double_sf/sep_176.0/TARGET_ref_0_sep_176.0_contr_1_ph_0.0_sf_0.05_CW.png]

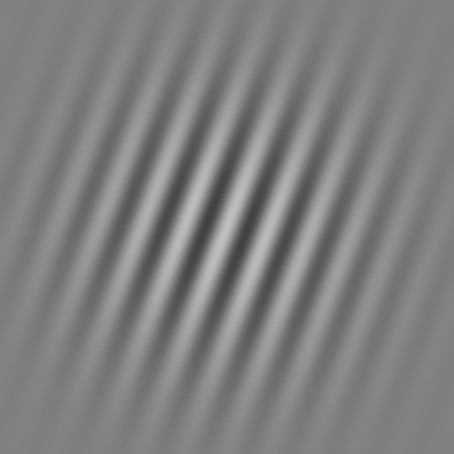

Supplement: S2 Data — (ZIP) [file pcbi.1014553.s010.zip › Curriculum-VPL-main/model_code/StimulusImages/SG_train_double_sf/sep_159.0/TARGET_ref_0_sep_159.0_contr_1_ph_0.0_sf_0.05_CCW.png]

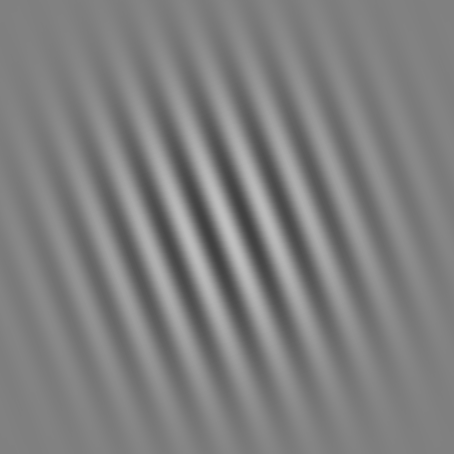

Supplement: S2 Data — (ZIP) [file pcbi.1014553.s010.zip › Curriculum-VPL-main/model_code/StimulusImages/SG_train_double_sf/sep_159.0/TARGET_ref_0_sep_159.0_contr_1_ph_0.0_sf_0.05_CW.png]

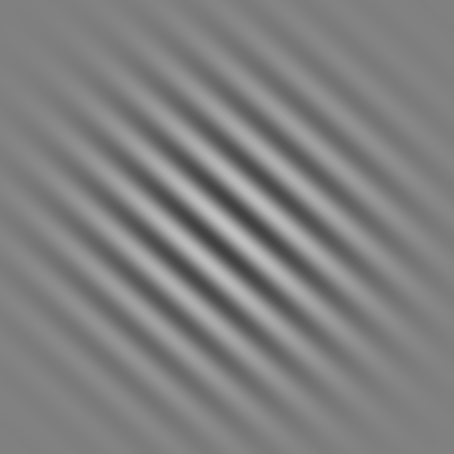

Supplement: S2 Data — (ZIP) [file pcbi.1014553.s010.zip › Curriculum-VPL-main/model_code/StimulusImages/SG_train_double_sf/sep_132.0/TARGET_ref_0_sep_132.0_contr_1_ph_0.0_sf_0.05_CW.png]

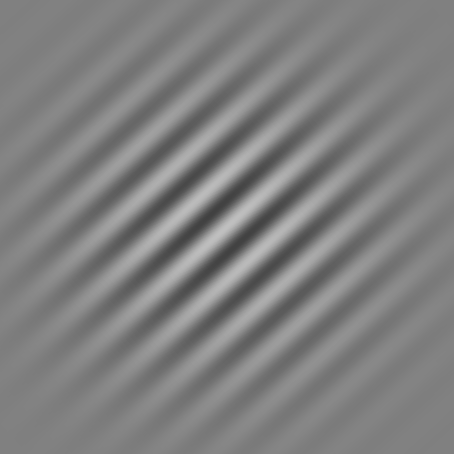

Supplement: S2 Data — (ZIP) [file pcbi.1014553.s010.zip › Curriculum-VPL-main/model_code/StimulusImages/SG_train_double_sf/sep_132.0/TARGET_ref_0_sep_132.0_contr_1_ph_0.0_sf_0.05_CCW.png]

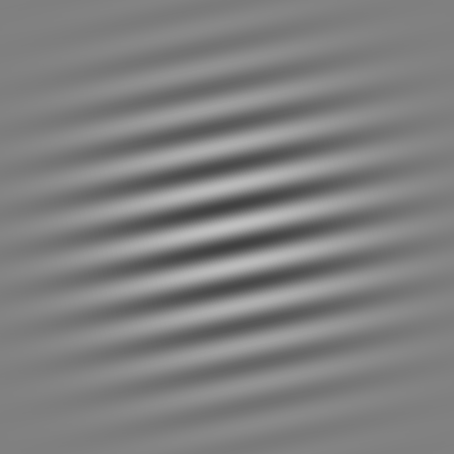

Supplement: S2 Data — (ZIP) [file pcbi.1014553.s010.zip › Curriculum-VPL-main/model_code/StimulusImages/SG_train_double_sf/sep_78.0/TARGET_ref_0_sep_78.0_contr_1_ph_0.0_sf_0.05_CW.png]

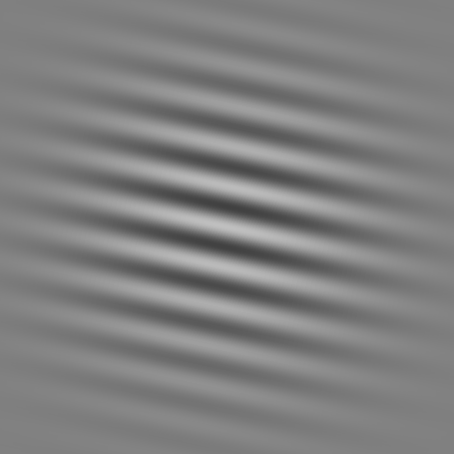

Supplement: S2 Data — (ZIP) [file pcbi.1014553.s010.zip › Curriculum-VPL-main/model_code/StimulusImages/SG_train_double_sf/sep_78.0/TARGET_ref_0_sep_78.0_contr_1_ph_0.0_sf_0.05_CCW.png]

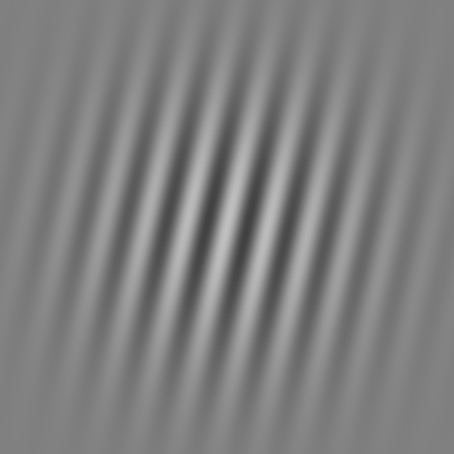

Supplement: S2 Data — (ZIP) [file pcbi.1014553.s010.zip › Curriculum-VPL-main/model_code/StimulusImages/SG_train_double_sf/sep_13.0/TARGET_ref_0_sep_13.0_contr_1_ph_0.0_sf_0.05_CW.png]

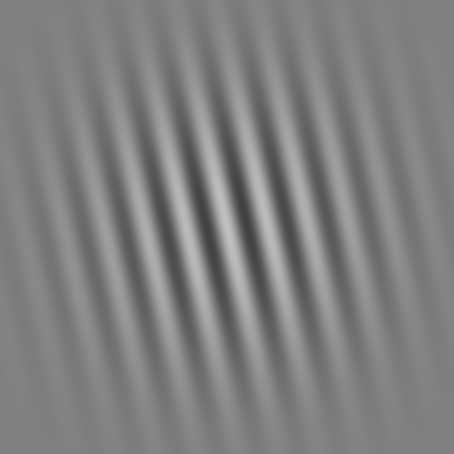

Supplement: S2 Data — (ZIP) [file pcbi.1014553.s010.zip › Curriculum-VPL-main/model_code/StimulusImages/SG_train_double_sf/sep_13.0/TARGET_ref_0_sep_13.0_contr_1_ph_0.0_sf_0.05_CCW.png]

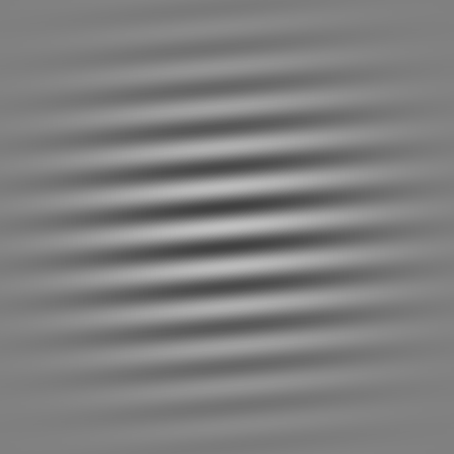

Supplement: S2 Data — (ZIP) [file pcbi.1014553.s010.zip › Curriculum-VPL-main/model_code/StimulusImages/SG_train_double_sf/sep_95.0/TARGET_ref_0_sep_95.0_contr_1_ph_0.0_sf_0.05_CCW.png]

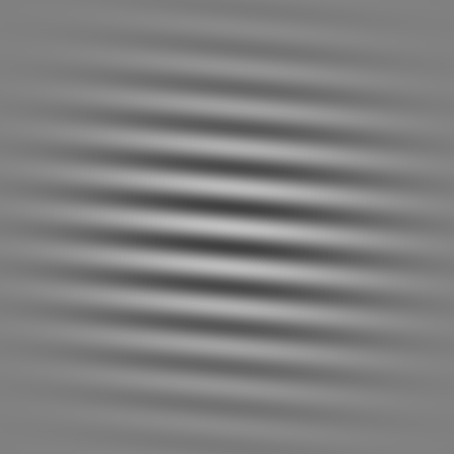

Supplement: S2 Data — (ZIP) [file pcbi.1014553.s010.zip › Curriculum-VPL-main/model_code/StimulusImages/SG_train_double_sf/sep_95.0/TARGET_ref_0_sep_95.0_contr_1_ph_0.0_sf_0.05_CW.png]

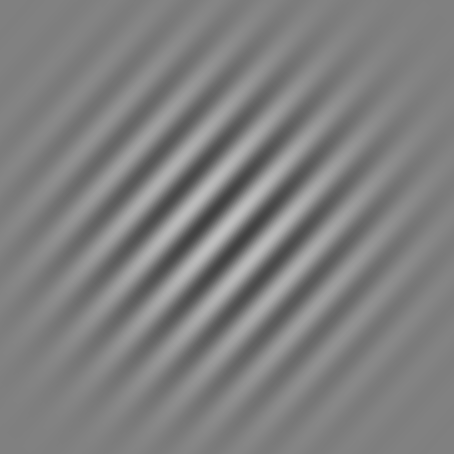

Supplement: S2 Data — (ZIP) [file pcbi.1014553.s010.zip › Curriculum-VPL-main/model_code/StimulusImages/SG_train_double_sf/sep_138.0/TARGET_ref_0_sep_138.0_contr_1_ph_0.0_sf_0.05_CCW.png]

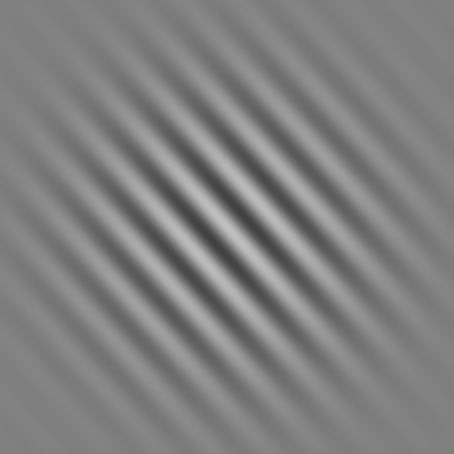

Supplement: S2 Data — (ZIP) [file pcbi.1014553.s010.zip › Curriculum-VPL-main/model_code/StimulusImages/SG_train_double_sf/sep_138.0/TARGET_ref_0_sep_138.0_contr_1_ph_0.0_sf_0.05_CW.png]

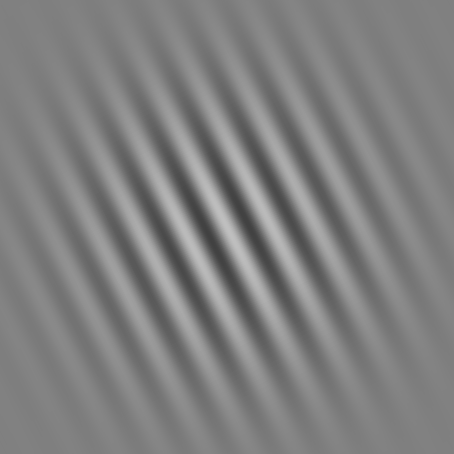

Supplement: S2 Data — (ZIP) [file pcbi.1014553.s010.zip › Curriculum-VPL-main/model_code/StimulusImages/SG_train_double_sf/sep_153.0/TARGET_ref_0_sep_153.0_contr_1_ph_0.0_sf_0.05_CW.png]

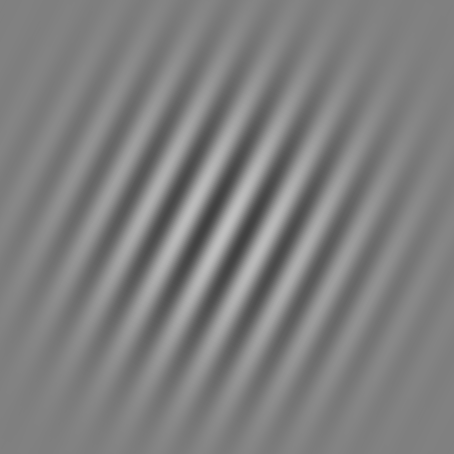

Supplement: S2 Data — (ZIP) [file pcbi.1014553.s010.zip › Curriculum-VPL-main/model_code/StimulusImages/SG_train_double_sf/sep_153.0/TARGET_ref_0_sep_153.0_contr_1_ph_0.0_sf_0.05_CCW.png]

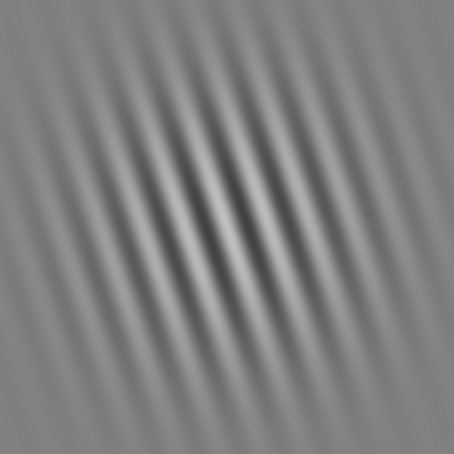

Supplement: S2 Data — (ZIP) [file pcbi.1014553.s010.zip › Curriculum-VPL-main/model_code/StimulusImages/SG_train_double_sf/sep_19.0/TARGET_ref_0_sep_19.0_contr_1_ph_0.0_sf_0.05_CCW.png]

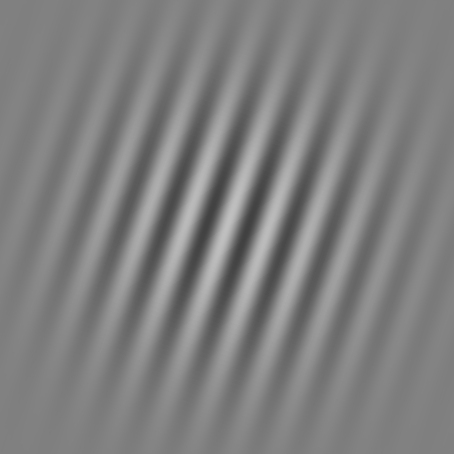

Supplement: S2 Data — (ZIP) [file pcbi.1014553.s010.zip › Curriculum-VPL-main/model_code/StimulusImages/SG_train_double_sf/sep_19.0/TARGET_ref_0_sep_19.0_contr_1_ph_0.0_sf_0.05_CW.png]

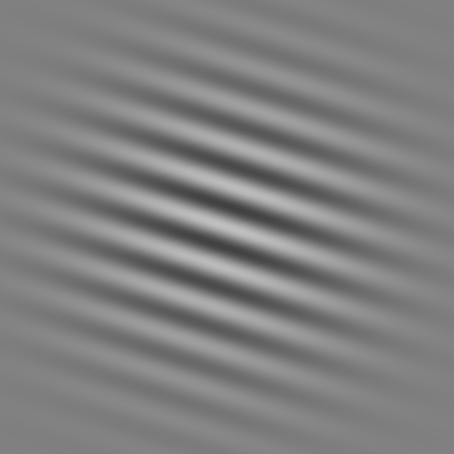

Supplement: S2 Data — (ZIP) [file pcbi.1014553.s010.zip › Curriculum-VPL-main/model_code/StimulusImages/SG_train_double_sf/sep_72.0/TARGET_ref_0_sep_72.0_contr_1_ph_0.0_sf_0.05_CCW.png]

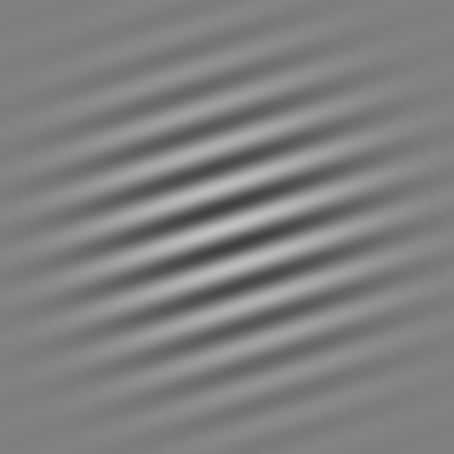

Supplement: S2 Data — (ZIP) [file pcbi.1014553.s010.zip › Curriculum-VPL-main/model_code/StimulusImages/SG_train_double_sf/sep_72.0/TARGET_ref_0_sep_72.0_contr_1_ph_0.0_sf_0.05_CW.png]

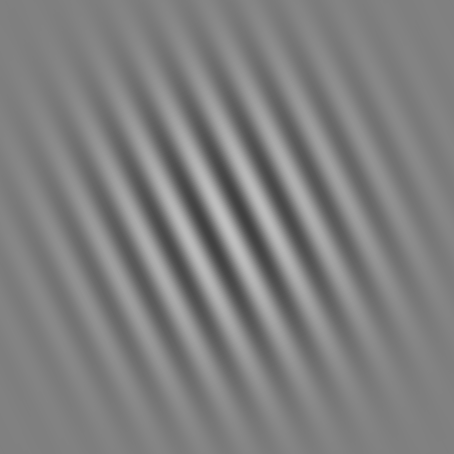

Supplement: S2 Data — (ZIP) [file pcbi.1014553.s010.zip › Curriculum-VPL-main/model_code/StimulusImages/SG_train_double_sf/sep_26.0/TARGET_ref_0_sep_26.0_contr_1_ph_0.0_sf_0.05_CCW.png]

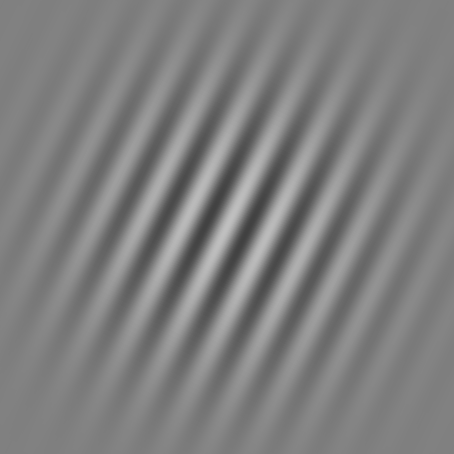

Supplement: S2 Data — (ZIP) [file pcbi.1014553.s010.zip › Curriculum-VPL-main/model_code/StimulusImages/SG_train_double_sf/sep_26.0/TARGET_ref_0_sep_26.0_contr_1_ph_0.0_sf_0.05_CW.png]

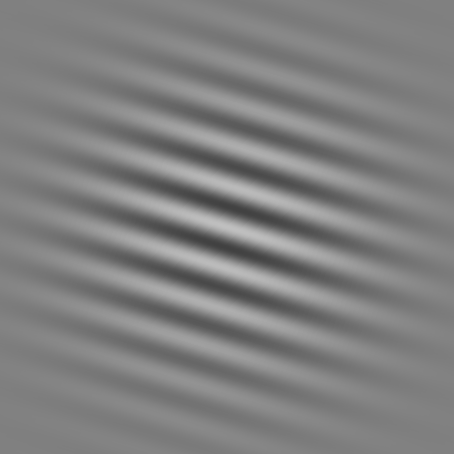

Supplement: S2 Data — (ZIP) [file pcbi.1014553.s010.zip › Curriculum-VPL-main/model_code/StimulusImages/SG_train_double_sf/sep_107.0/TARGET_ref_0_sep_107.0_contr_1_ph_0.0_sf_0.05_CW.png]

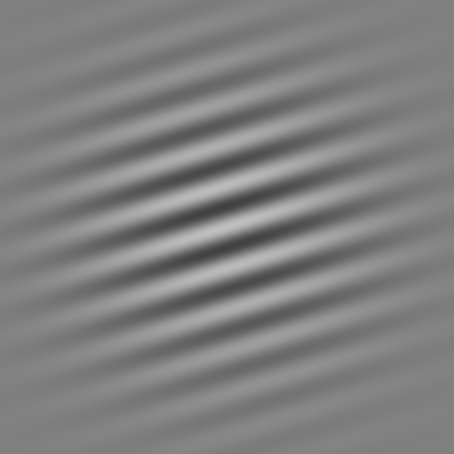

Supplement: S2 Data — (ZIP) [file pcbi.1014553.s010.zip › Curriculum-VPL-main/model_code/StimulusImages/SG_train_double_sf/sep_107.0/TARGET_ref_0_sep_107.0_contr_1_ph_0.0_sf_0.05_CCW.png]

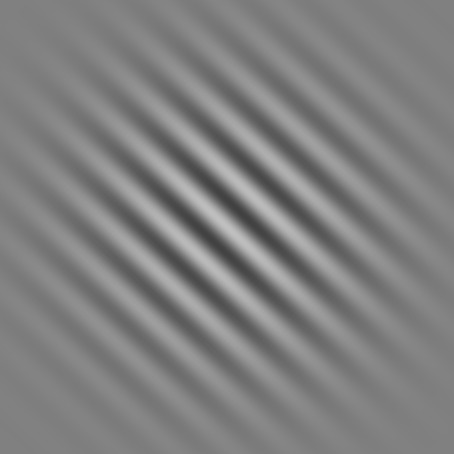

Supplement: S2 Data — (ZIP) [file pcbi.1014553.s010.zip › Curriculum-VPL-main/model_code/StimulusImages/SG_train_double_sf/sep_47.0/TARGET_ref_0_sep_47.0_contr_1_ph_0.0_sf_0.05_CCW.png]

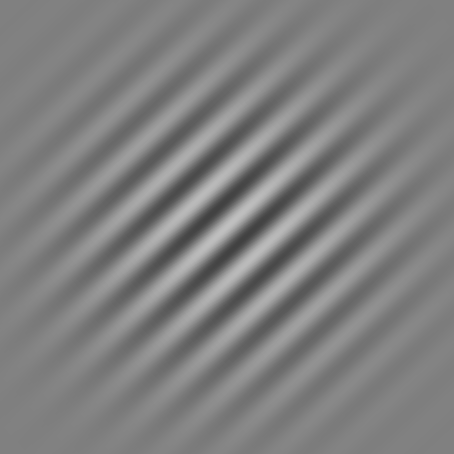

Supplement: S2 Data — (ZIP) [file pcbi.1014553.s010.zip › Curriculum-VPL-main/model_code/StimulusImages/SG_train_double_sf/sep_47.0/TARGET_ref_0_sep_47.0_contr_1_ph_0.0_sf_0.05_CW.png]

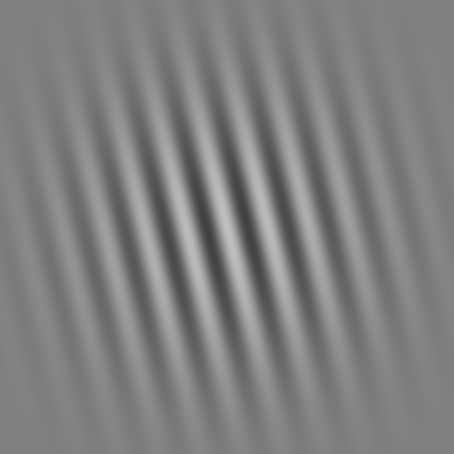

Supplement: S2 Data — (ZIP) [file pcbi.1014553.s010.zip › Curriculum-VPL-main/model_code/StimulusImages/SG_train_double_sf/sep_166.0/TARGET_ref_0_sep_166.0_contr_1_ph_0.0_sf_0.05_CW.png]

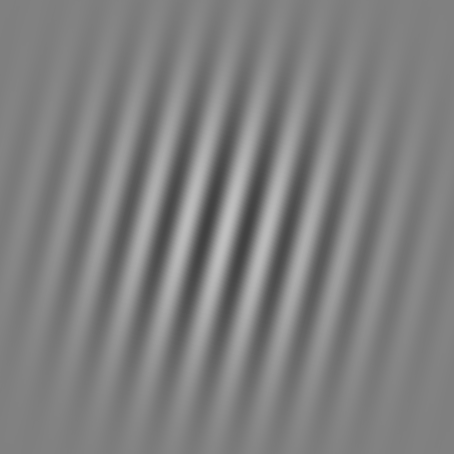

Supplement: S2 Data — (ZIP) [file pcbi.1014553.s010.zip › Curriculum-VPL-main/model_code/StimulusImages/SG_train_double_sf/sep_166.0/TARGET_ref_0_sep_166.0_contr_1_ph_0.0_sf_0.05_CCW.png]

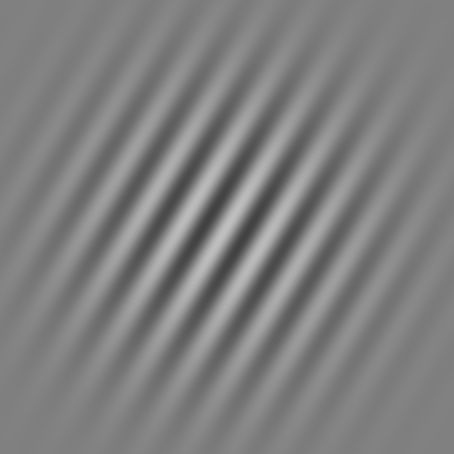

Supplement: S2 Data — (ZIP) [file pcbi.1014553.s010.zip › Curriculum-VPL-main/model_code/StimulusImages/SG_train_double_sf/sep_149.0/TARGET_ref_0_sep_149.0_contr_1_ph_0.0_sf_0.05_CCW.png]

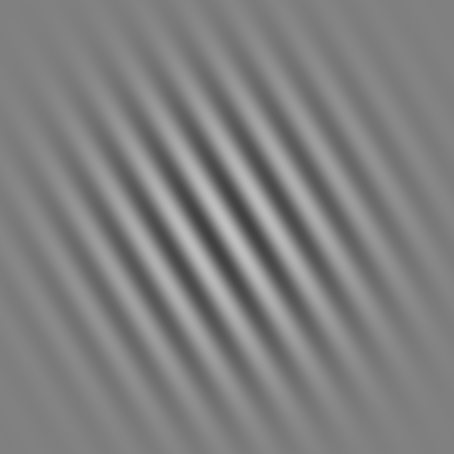

Supplement: S2 Data — (ZIP) [file pcbi.1014553.s010.zip › Curriculum-VPL-main/model_code/StimulusImages/SG_train_double_sf/sep_149.0/TARGET_ref_0_sep_149.0_contr_1_ph_0.0_sf_0.05_CW.png]

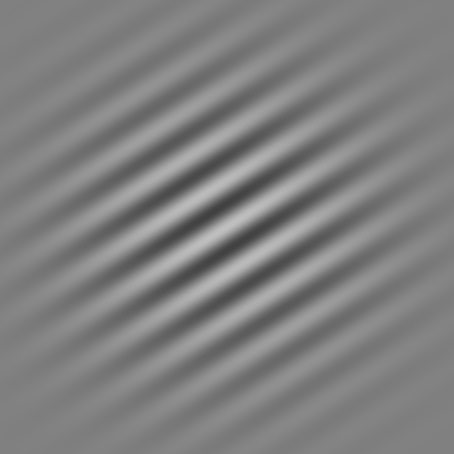

Supplement: S2 Data — (ZIP) [file pcbi.1014553.s010.zip › Curriculum-VPL-main/model_code/StimulusImages/SG_train_double_sf/sep_122.0/TARGET_ref_0_sep_122.0_contr_1_ph_0.0_sf_0.05_CCW.png]

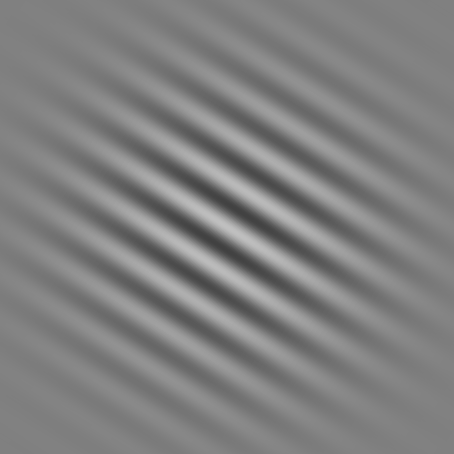

Supplement: S2 Data — (ZIP) [file pcbi.1014553.s010.zip › Curriculum-VPL-main/model_code/StimulusImages/SG_train_double_sf/sep_122.0/TARGET_ref_0_sep_122.0_contr_1_ph_0.0_sf_0.05_CW.png]

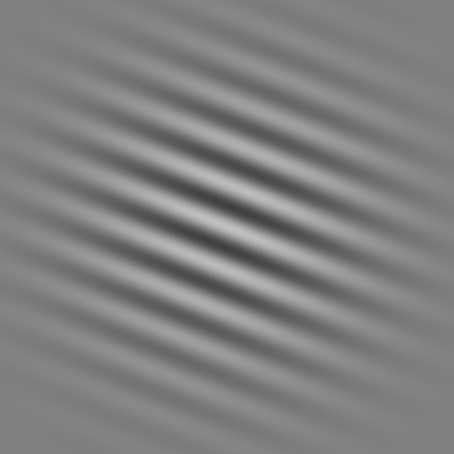

Supplement: S2 Data — (ZIP) [file pcbi.1014553.s010.zip › Curriculum-VPL-main/model_code/StimulusImages/SG_train_double_sf/sep_68.0/TARGET_ref_0_sep_68.0_contr_1_ph_0.0_sf_0.05_CCW.png]

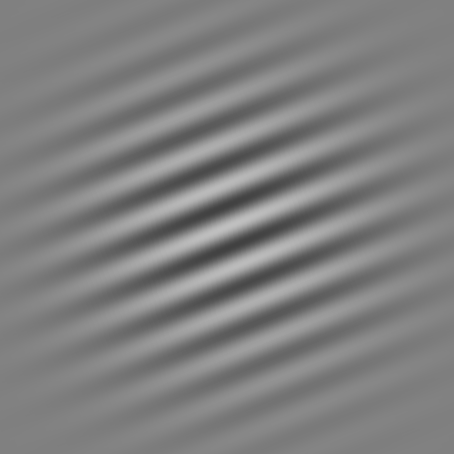

Supplement: S2 Data — (ZIP) [file pcbi.1014553.s010.zip › Curriculum-VPL-main/model_code/StimulusImages/SG_train_double_sf/sep_68.0/TARGET_ref_0_sep_68.0_contr_1_ph_0.0_sf_0.05_CW.png]

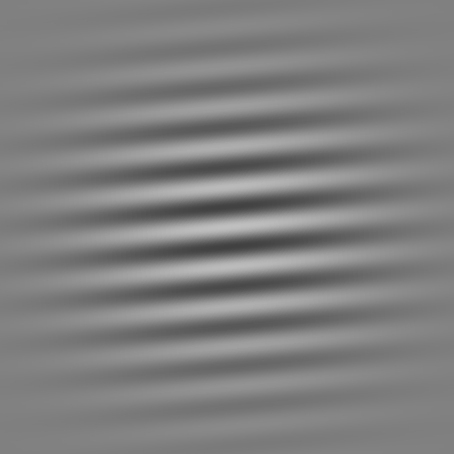

Supplement: S2 Data — (ZIP) [file pcbi.1014553.s010.zip › Curriculum-VPL-main/model_code/StimulusImages/SG_train_double_sf/sep_85.0/TARGET_ref_0_sep_85.0_contr_1_ph_0.0_sf_0.05_CW.png]

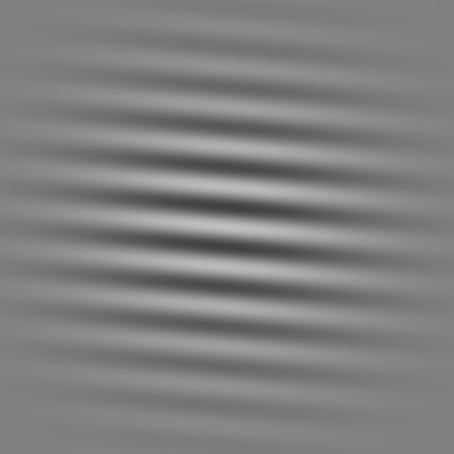

Supplement: S2 Data — (ZIP) [file pcbi.1014553.s010.zip › Curriculum-VPL-main/model_code/StimulusImages/SG_train_double_sf/sep_85.0/TARGET_ref_0_sep_85.0_contr_1_ph_0.0_sf_0.05_CCW.png]

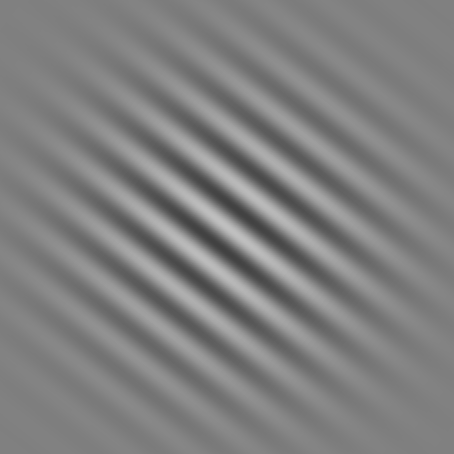

Supplement: S2 Data — (ZIP) [file pcbi.1014553.s010.zip › Curriculum-VPL-main/model_code/StimulusImages/SG_train_double_sf/sep_128.0/TARGET_ref_0_sep_128.0_contr_1_ph_0.0_sf_0.05_CW.png]

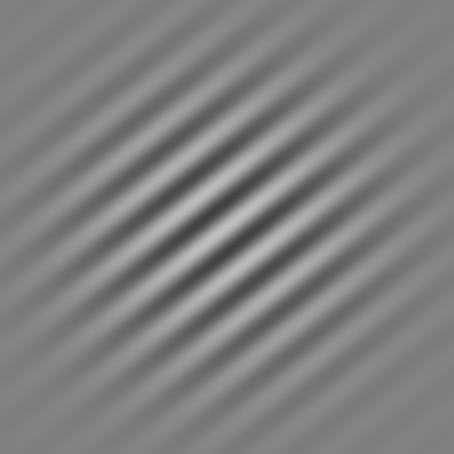

Supplement: S2 Data — (ZIP) [file pcbi.1014553.s010.zip › Curriculum-VPL-main/model_code/StimulusImages/SG_train_double_sf/sep_128.0/TARGET_ref_0_sep_128.0_contr_1_ph_0.0_sf_0.05_CCW.png]

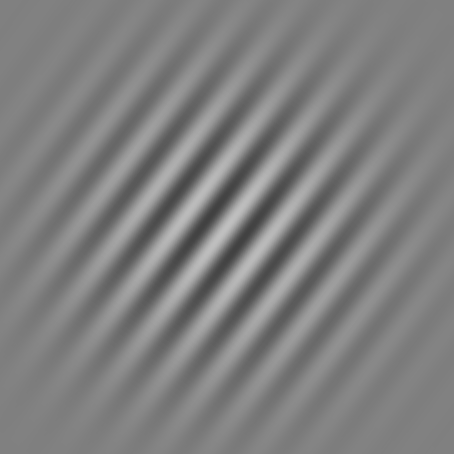

Supplement: S2 Data — (ZIP) [file pcbi.1014553.s010.zip › Curriculum-VPL-main/model_code/StimulusImages/SG_train_double_sf/sep_143.0/TARGET_ref_0_sep_143.0_contr_1_ph_0.0_sf_0.05_CCW.png]

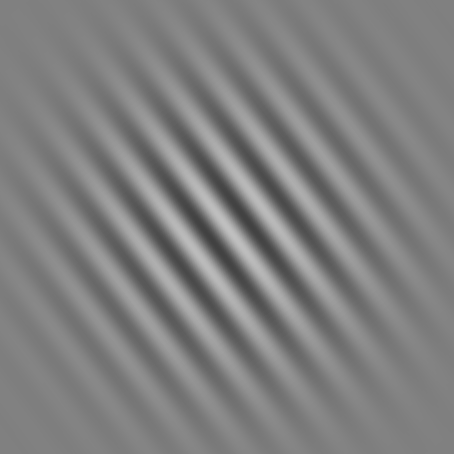

Supplement: S2 Data — (ZIP) [file pcbi.1014553.s010.zip › Curriculum-VPL-main/model_code/StimulusImages/SG_train_double_sf/sep_143.0/TARGET_ref_0_sep_143.0_contr_1_ph_0.0_sf_0.05_CW.png]

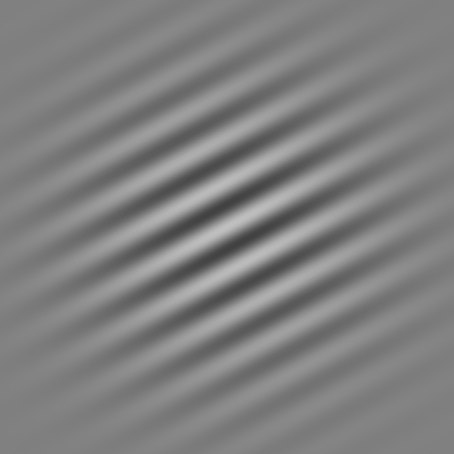

Supplement: S2 Data — (ZIP) [file pcbi.1014553.s010.zip › Curriculum-VPL-main/model_code/StimulusImages/SG_train_double_sf/sep_62.0/TARGET_ref_0_sep_62.0_contr_1_ph_0.0_sf_0.05_CW.png]

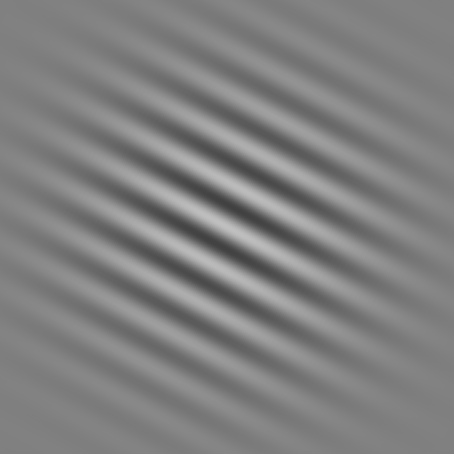

Supplement: S2 Data — (ZIP) [file pcbi.1014553.s010.zip › Curriculum-VPL-main/model_code/StimulusImages/SG_train_double_sf/sep_62.0/TARGET_ref_0_sep_62.0_contr_1_ph_0.0_sf_0.05_CCW.png]

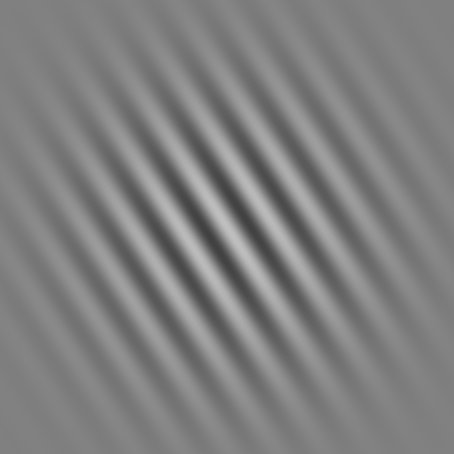

Supplement: S2 Data — (ZIP) [file pcbi.1014553.s010.zip › Curriculum-VPL-main/model_code/StimulusImages/SG_train_double_sf/sep_147.0/TARGET_ref_0_sep_147.0_contr_1_ph_0.0_sf_0.05_CW.png]

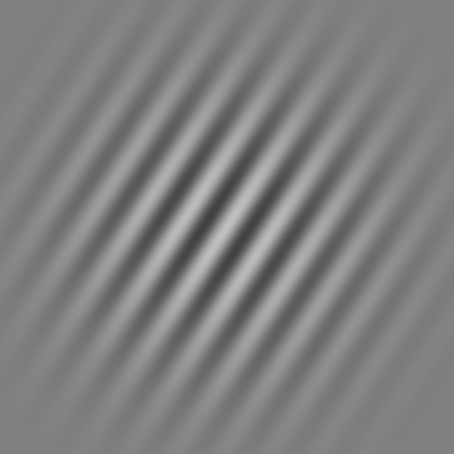

Supplement: S2 Data — (ZIP) [file pcbi.1014553.s010.zip › Curriculum-VPL-main/model_code/StimulusImages/SG_train_double_sf/sep_147.0/TARGET_ref_0_sep_147.0_contr_1_ph_0.0_sf_0.05_CCW.png]
