# Supplementary material for: The curriculum effect in visual learning: The role of readout dimensionality
Source: PLoS Comput Biol. 2026 Jul 24;22(7):e1014553. doi: 10.1371/journal.pcbi.1014553 (PMC13432104; doi:10.1371/journal.pcbi.1014553)
Supplement: S1 Appendix — (PDF) [file pcbi.1014553.s008.pdf]

Appendix S1. Results for different model backbones

To investigate the robustness of our results across different model backbone choices, we replaced our AlexNet backbone with two other ANN model architectures: GoogLeNet [1] and EfficientNet [2] with added skip connections from each layer to the readout. These models were selected because they could solve the task without extensive hyperparameter engineering and optimization. Additionally, like AlexNet, these models are small convolutional backbones without complex information pathways or spatial averaging. We found that these models show similar patterns of transfer accuracy versus task difficulty, and that the transfer accuracy is negatively correlated with the dimensionality of the readout subspace (Fig. S7; r=-0.89, p=0.046). However, our results did not extend to other larger and more complex model backbones (see Methods for a complete list of architectures), which are less accurate approximations of single-neuron tuning properties in the visual system [3]. As we showed in Fig. 5, single-neuron tuning properties are key to the observed relationship between dimensionality and generalization, so we conjecture that the models with more similar single-neuron tunings to the brain should better reflect learning generalization in humans. Another potential reason for this discrepancy relates to the preserved fine orientation information required at the output of the model due to the nature of the task. In most of the pretrained convolutional architectures, particularly the deeper models, the fine orientation information diminishes at the output due to spatial averaging through the model. Adding skip connections from every layer to the output could partially solve this problem, but for larger models this makes training intractable without extensive engineering and hyperparameter optimization.

References

1. Szegedy C, Liu W, Jia Y, Sermanet P, Reed S, Anguelov D, et al. Going Deeper with Convolutions. Computer Vision and Pattern Recognition (CVPR). 2015. Available: http://arxiv.org/abs/1409.4842

2. Tan M, Le Q. EfficientNet: Rethinking Model Scaling for Convolutional Neural Networks. In: Chaudhuri K, Salakhutdinov R, editors. Proceedings of the 36th International Conference on Machine Learning. PMLR; 2019. pp. 6105–6114. Available: https://proceedings.mlr.press/v97/tan19a.html

3. Khosla M, Williams AH, McDermott J, Kanwisher N. Privileged representational axes in biological and artificial neural networks. bioRxiv. 2024. doi:10.1101/2024.06.20.599957
