# Supplementary figures and images for: The curriculum effect in visual learning: The role of readout dimensionality (part 2 of 2)
Source: PLoS Comput Biol. 2026 Jul 24;22(7):e1014553. doi: 10.1371/journal.pcbi.1014553 (PMC13432104; doi:10.1371/journal.pcbi.1014553)

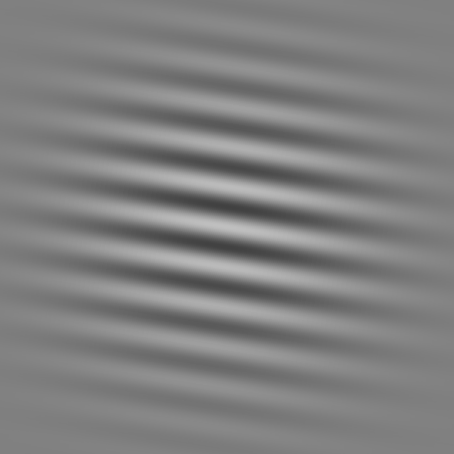

Supplement: S2 Data — (ZIP) [file pcbi.1014553.s010.zip › Curriculum-VPL-main/model_code/StimulusImages/SG_train_double_sf/sep_81.0/TARGET_ref_0_sep_81.0_contr_1_ph_0.0_sf_0.05_CCW.png]

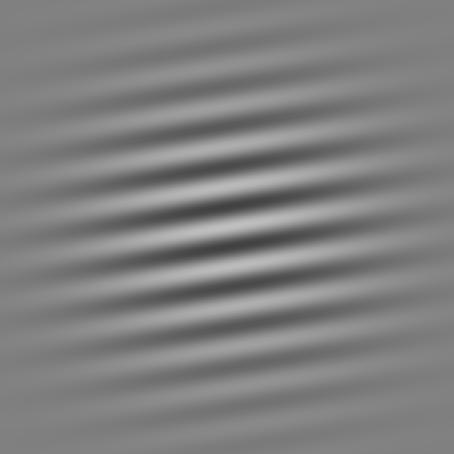

Supplement: S2 Data — (ZIP) [file pcbi.1014553.s010.zip › Curriculum-VPL-main/model_code/StimulusImages/SG_train_double_sf/sep_81.0/TARGET_ref_0_sep_81.0_contr_1_ph_0.0_sf_0.05_CW.png]

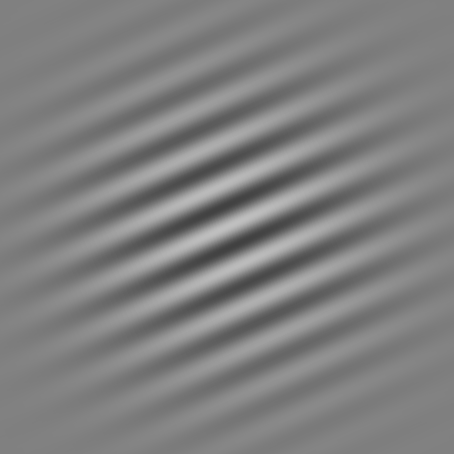

Supplement: S2 Data — (ZIP) [file pcbi.1014553.s010.zip › Curriculum-VPL-main/model_code/StimulusImages/SG_train_double_sf/sep_66.0/TARGET_ref_0_sep_66.0_contr_1_ph_0.0_sf_0.05_CW.png]

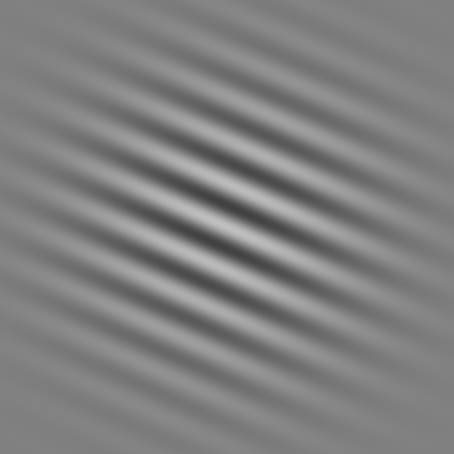

Supplement: S2 Data — (ZIP) [file pcbi.1014553.s010.zip › Curriculum-VPL-main/model_code/StimulusImages/SG_train_double_sf/sep_66.0/TARGET_ref_0_sep_66.0_contr_1_ph_0.0_sf_0.05_CCW.png]

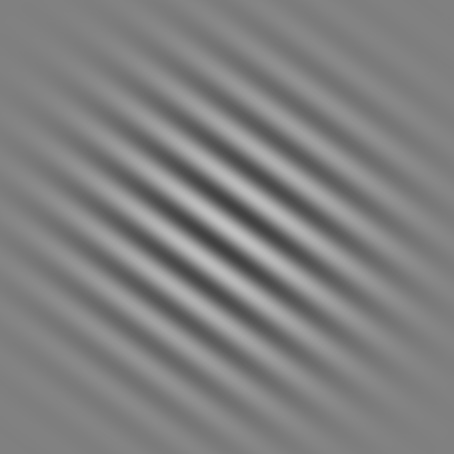

Supplement: S2 Data — (ZIP) [file pcbi.1014553.s010.zip › Curriculum-VPL-main/model_code/StimulusImages/SG_train_double_sf/sep_126.0/TARGET_ref_0_sep_126.0_contr_1_ph_0.0_sf_0.05_CW.png]

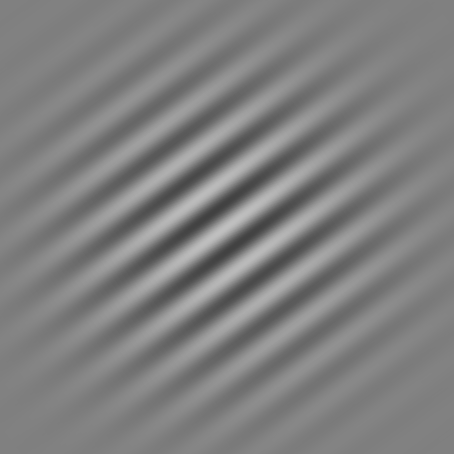

Supplement: S2 Data — (ZIP) [file pcbi.1014553.s010.zip › Curriculum-VPL-main/model_code/StimulusImages/SG_train_double_sf/sep_126.0/TARGET_ref_0_sep_126.0_contr_1_ph_0.0_sf_0.05_CCW.png]

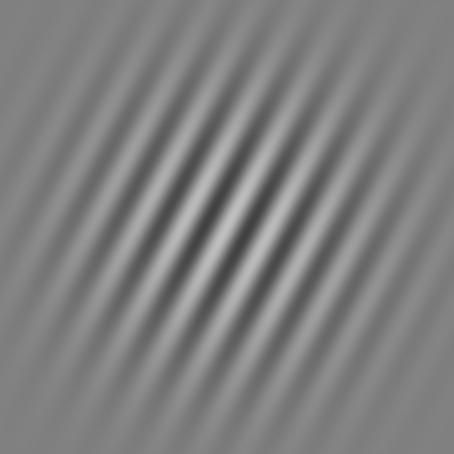

Supplement: S2 Data — (ZIP) [file pcbi.1014553.s010.zip › Curriculum-VPL-main/model_code/StimulusImages/SG_train_double_sf/sep_28.0/TARGET_ref_0_sep_28.0_contr_1_ph_0.0_sf_0.05_CW.png]

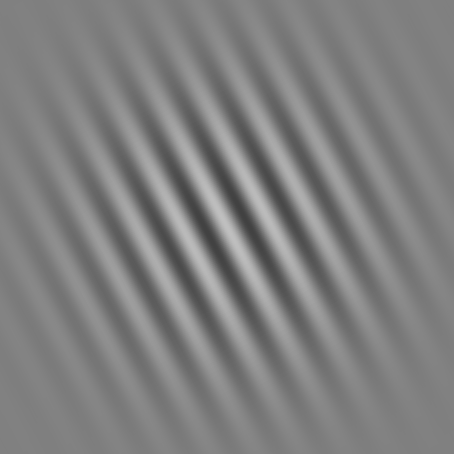

Supplement: S2 Data — (ZIP) [file pcbi.1014553.s010.zip › Curriculum-VPL-main/model_code/StimulusImages/SG_train_double_sf/sep_28.0/TARGET_ref_0_sep_28.0_contr_1_ph_0.0_sf_0.05_CCW.png]

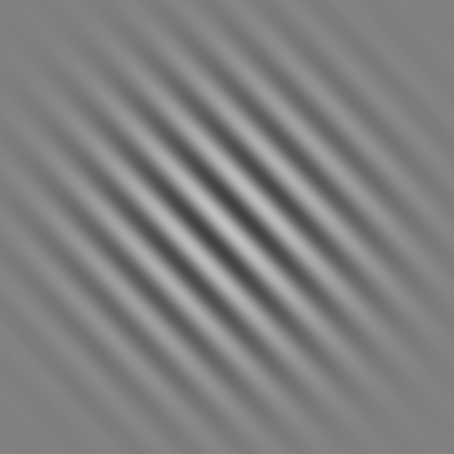

Supplement: S2 Data — (ZIP) [file pcbi.1014553.s010.zip › Curriculum-VPL-main/model_code/StimulusImages/SG_train_double_sf/sep_43.0/TARGET_ref_0_sep_43.0_contr_1_ph_0.0_sf_0.05_CCW.png]

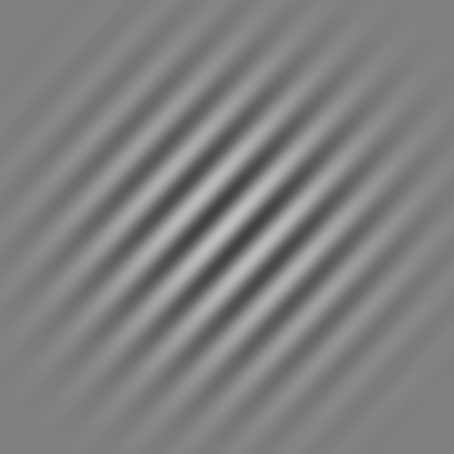

Supplement: S2 Data — (ZIP) [file pcbi.1014553.s010.zip › Curriculum-VPL-main/model_code/StimulusImages/SG_train_double_sf/sep_43.0/TARGET_ref_0_sep_43.0_contr_1_ph_0.0_sf_0.05_CW.png]

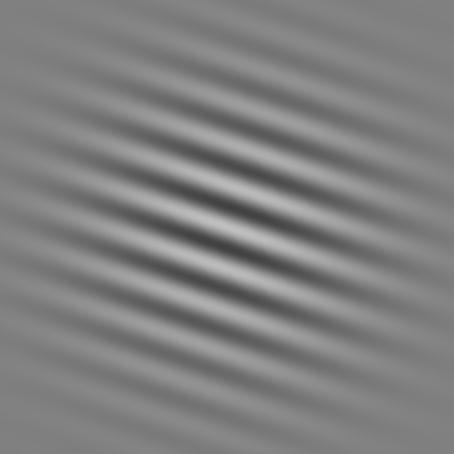

Supplement: S2 Data — (ZIP) [file pcbi.1014553.s010.zip › Curriculum-VPL-main/model_code/StimulusImages/SG_train_double_sf/sep_109.0/TARGET_ref_0_sep_109.0_contr_1_ph_0.0_sf_0.05_CW.png]

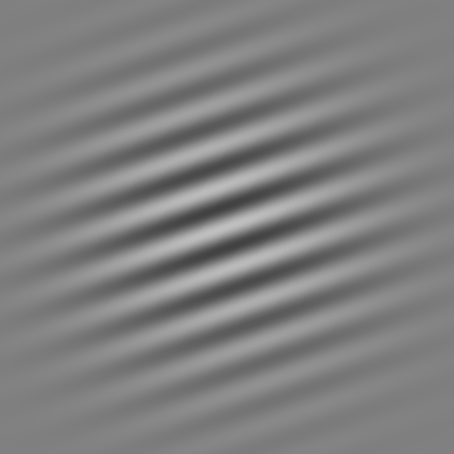

Supplement: S2 Data — (ZIP) [file pcbi.1014553.s010.zip › Curriculum-VPL-main/model_code/StimulusImages/SG_train_double_sf/sep_109.0/TARGET_ref_0_sep_109.0_contr_1_ph_0.0_sf_0.05_CCW.png]

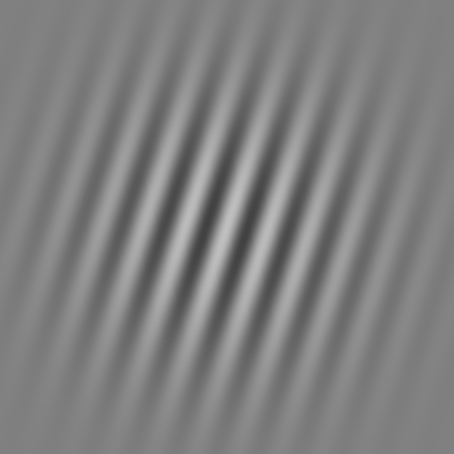

Supplement: S2 Data — (ZIP) [file pcbi.1014553.s010.zip › Curriculum-VPL-main/model_code/StimulusImages/SG_train_double_sf/sep_162.0/TARGET_ref_0_sep_162.0_contr_1_ph_0.0_sf_0.05_CCW.png]

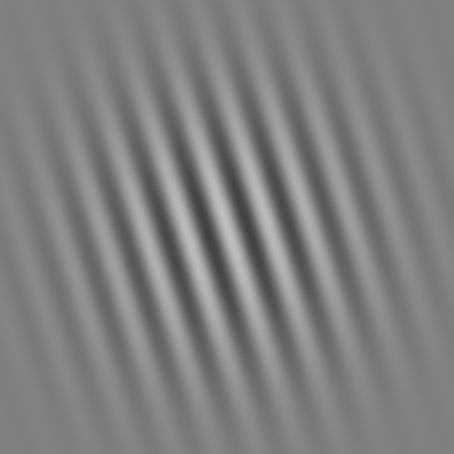

Supplement: S2 Data — (ZIP) [file pcbi.1014553.s010.zip › Curriculum-VPL-main/model_code/StimulusImages/SG_train_double_sf/sep_162.0/TARGET_ref_0_sep_162.0_contr_1_ph_0.0_sf_0.05_CW.png]

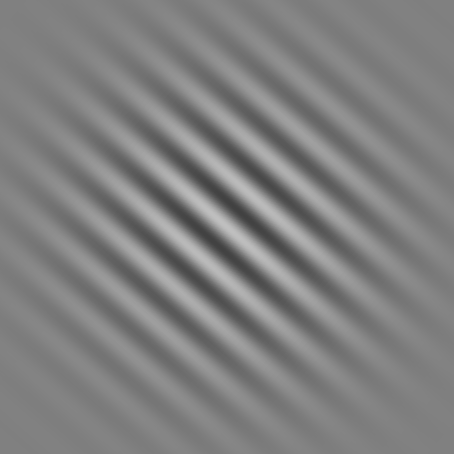

Supplement: S2 Data — (ZIP) [file pcbi.1014553.s010.zip › Curriculum-VPL-main/model_code/StimulusImages/SG_train_double_sf/sep_49.0/TARGET_ref_0_sep_49.0_contr_1_ph_0.0_sf_0.05_CCW.png]

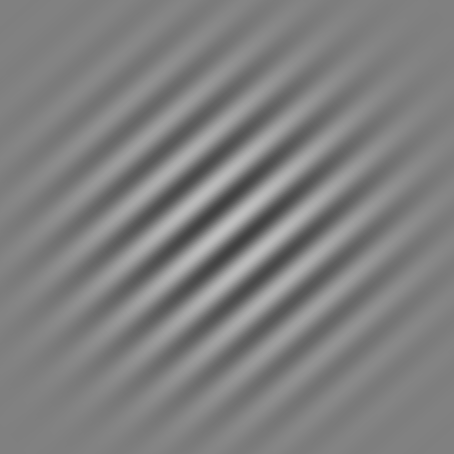

Supplement: S2 Data — (ZIP) [file pcbi.1014553.s010.zip › Curriculum-VPL-main/model_code/StimulusImages/SG_train_double_sf/sep_49.0/TARGET_ref_0_sep_49.0_contr_1_ph_0.0_sf_0.05_CW.png]

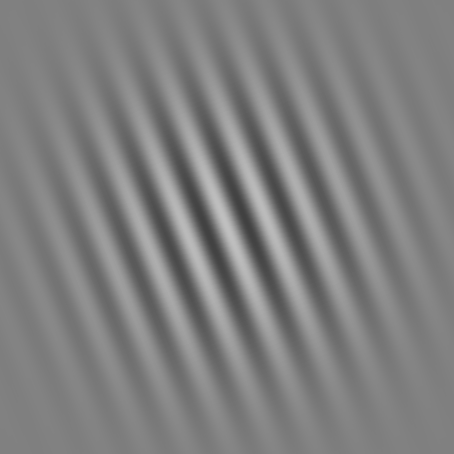

Supplement: S2 Data — (ZIP) [file pcbi.1014553.s010.zip › Curriculum-VPL-main/model_code/StimulusImages/SG_train_double_sf/sep_22.0/TARGET_ref_0_sep_22.0_contr_1_ph_0.0_sf_0.05_CCW.png]

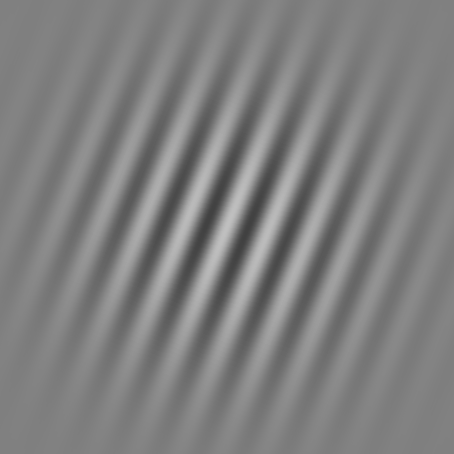

Supplement: S2 Data — (ZIP) [file pcbi.1014553.s010.zip › Curriculum-VPL-main/model_code/StimulusImages/SG_train_double_sf/sep_22.0/TARGET_ref_0_sep_22.0_contr_1_ph_0.0_sf_0.05_CW.png]

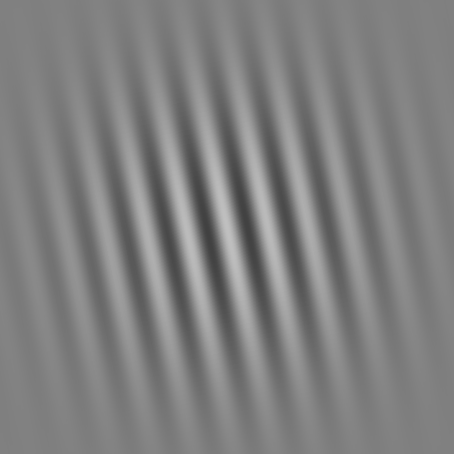

Supplement: S2 Data — (ZIP) [file pcbi.1014553.s010.zip › Curriculum-VPL-main/model_code/StimulusImages/SG_train_double_sf/sep_168.0/TARGET_ref_0_sep_168.0_contr_1_ph_0.0_sf_0.05_CW.png]

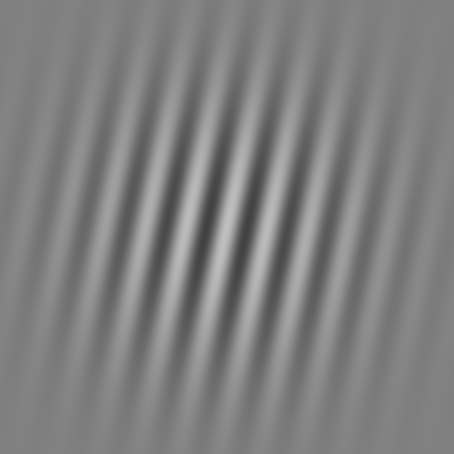

Supplement: S2 Data — (ZIP) [file pcbi.1014553.s010.zip › Curriculum-VPL-main/model_code/StimulusImages/SG_train_double_sf/sep_168.0/TARGET_ref_0_sep_168.0_contr_1_ph_0.0_sf_0.05_CCW.png]

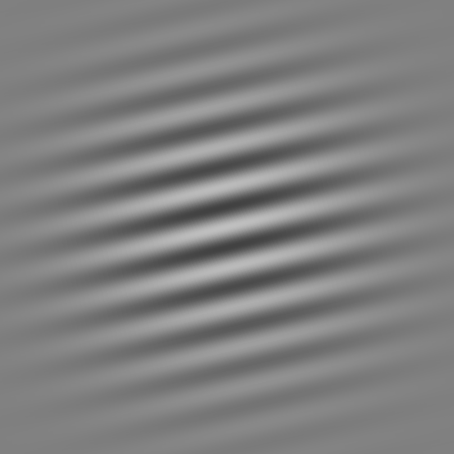

Supplement: S2 Data — (ZIP) [file pcbi.1014553.s010.zip › Curriculum-VPL-main/model_code/StimulusImages/SG_train_double_sf/sep_103.0/TARGET_ref_0_sep_103.0_contr_1_ph_0.0_sf_0.05_CCW.png]

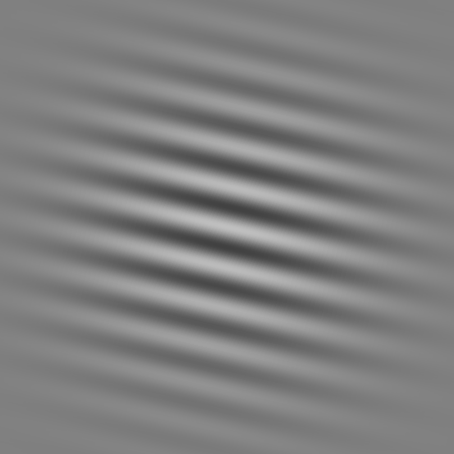

Supplement: S2 Data — (ZIP) [file pcbi.1014553.s010.zip › Curriculum-VPL-main/model_code/StimulusImages/SG_train_double_sf/sep_103.0/TARGET_ref_0_sep_103.0_contr_1_ph_0.0_sf_0.05_CW.png]

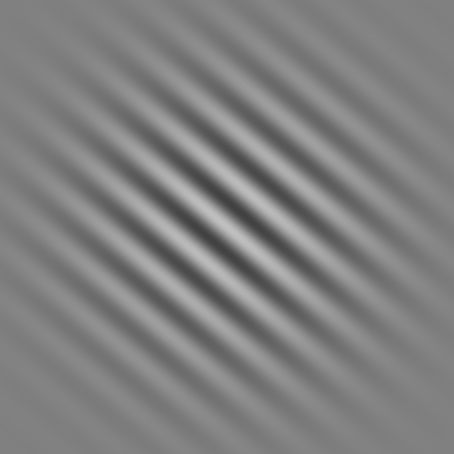

Supplement: S2 Data — (ZIP) [file pcbi.1014553.s010.zip › Curriculum-VPL-main/model_code/StimulusImages/SG_train_double_sf/sep_50.0/TARGET_ref_0_sep_50.0_contr_1_ph_0.0_sf_0.05_CCW.png]

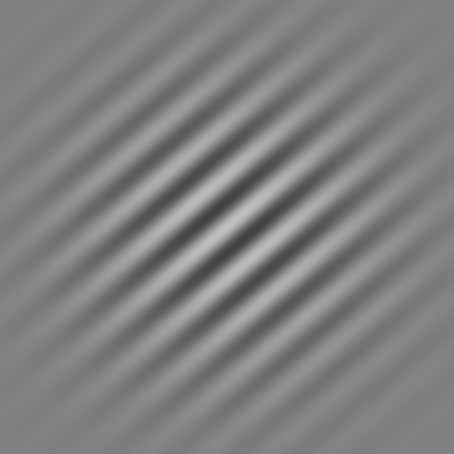

Supplement: S2 Data — (ZIP) [file pcbi.1014553.s010.zip › Curriculum-VPL-main/model_code/StimulusImages/SG_train_double_sf/sep_50.0/TARGET_ref_0_sep_50.0_contr_1_ph_0.0_sf_0.05_CW.png]

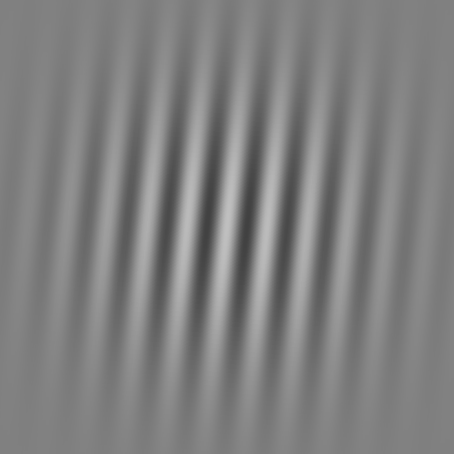

Supplement: S2 Data — (ZIP) [file pcbi.1014553.s010.zip › Curriculum-VPL-main/model_code/StimulusImages/SG_train_double_sf/sep_6.0/TARGET_ref_0_sep_6.0_contr_1_ph_0.0_sf_0.05_CW.png]

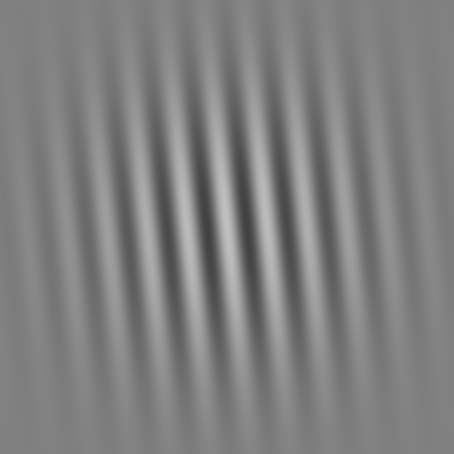

Supplement: S2 Data — (ZIP) [file pcbi.1014553.s010.zip › Curriculum-VPL-main/model_code/StimulusImages/SG_train_double_sf/sep_6.0/TARGET_ref_0_sep_6.0_contr_1_ph_0.0_sf_0.05_CCW.png]

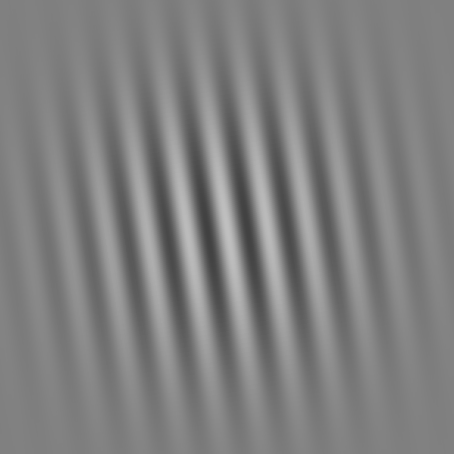

Supplement: S2 Data — (ZIP) [file pcbi.1014553.s010.zip › Curriculum-VPL-main/model_code/StimulusImages/SG_train_double_sf/sep_171.0/TARGET_ref_0_sep_171.0_contr_1_ph_0.0_sf_0.05_CW.png]

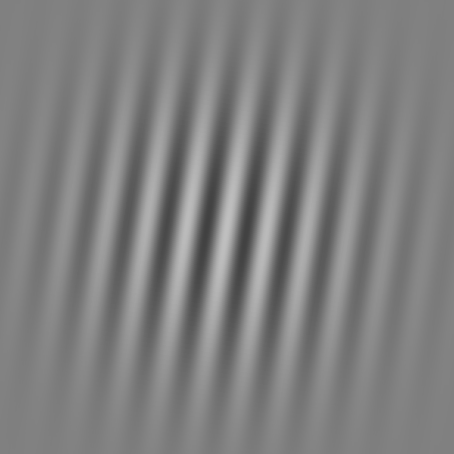

Supplement: S2 Data — (ZIP) [file pcbi.1014553.s010.zip › Curriculum-VPL-main/model_code/StimulusImages/SG_train_double_sf/sep_171.0/TARGET_ref_0_sep_171.0_contr_1_ph_0.0_sf_0.05_CCW.png]

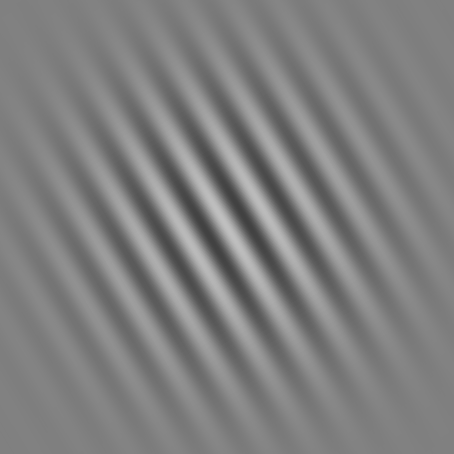

Supplement: S2 Data — (ZIP) [file pcbi.1014553.s010.zip › Curriculum-VPL-main/model_code/StimulusImages/SG_train_double_sf/sep_31.0/TARGET_ref_0_sep_31.0_contr_1_ph_0.0_sf_0.05_CCW.png]

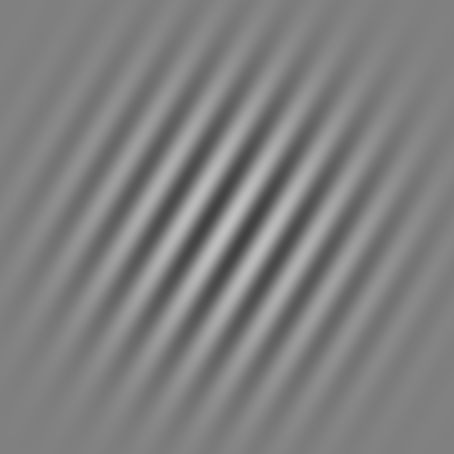

Supplement: S2 Data — (ZIP) [file pcbi.1014553.s010.zip › Curriculum-VPL-main/model_code/StimulusImages/SG_train_double_sf/sep_31.0/TARGET_ref_0_sep_31.0_contr_1_ph_0.0_sf_0.05_CW.png]

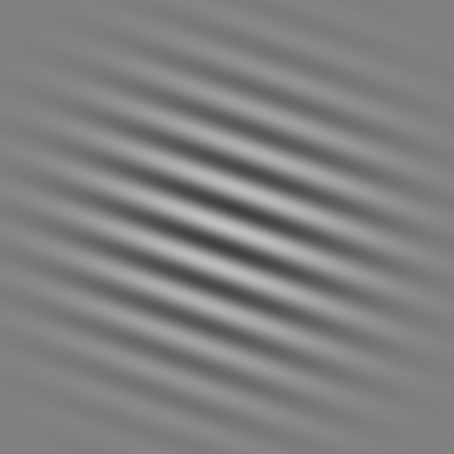

Supplement: S2 Data — (ZIP) [file pcbi.1014553.s010.zip › Curriculum-VPL-main/model_code/StimulusImages/SG_train_double_sf/sep_110.0/TARGET_ref_0_sep_110.0_contr_1_ph_0.0_sf_0.05_CW.png]

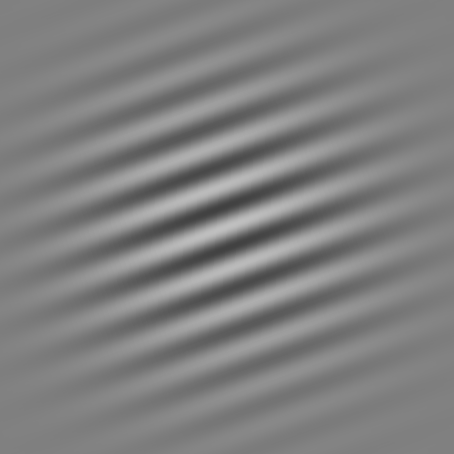

Supplement: S2 Data — (ZIP) [file pcbi.1014553.s010.zip › Curriculum-VPL-main/model_code/StimulusImages/SG_train_double_sf/sep_110.0/TARGET_ref_0_sep_110.0_contr_1_ph_0.0_sf_0.05_CCW.png]

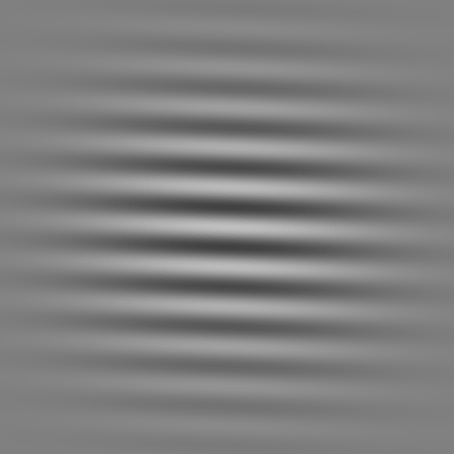

Supplement: S2 Data — (ZIP) [file pcbi.1014553.s010.zip › Curriculum-VPL-main/model_code/StimulusImages/SG_train_double_sf/sep_92.0/TARGET_ref_0_sep_92.0_contr_1_ph_0.0_sf_0.05_CW.png]

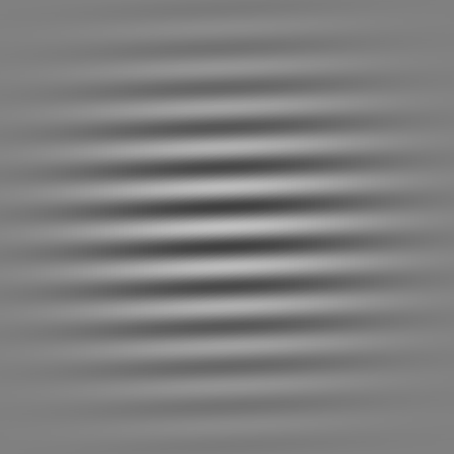

Supplement: S2 Data — (ZIP) [file pcbi.1014553.s010.zip › Curriculum-VPL-main/model_code/StimulusImages/SG_train_double_sf/sep_92.0/TARGET_ref_0_sep_92.0_contr_1_ph_0.0_sf_0.05_CCW.png]

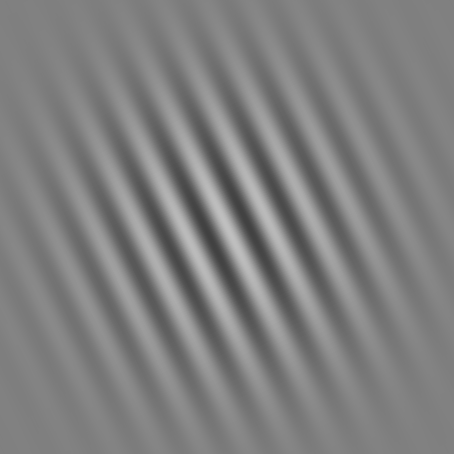

Supplement: S2 Data — (ZIP) [file pcbi.1014553.s010.zip › Curriculum-VPL-main/model_code/StimulusImages/SG_train_double_sf/sep_154.0/TARGET_ref_0_sep_154.0_contr_1_ph_0.0_sf_0.05_CW.png]

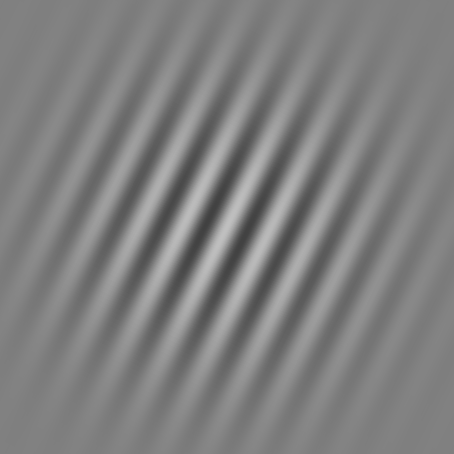

Supplement: S2 Data — (ZIP) [file pcbi.1014553.s010.zip › Curriculum-VPL-main/model_code/StimulusImages/SG_train_double_sf/sep_154.0/TARGET_ref_0_sep_154.0_contr_1_ph_0.0_sf_0.05_CCW.png]

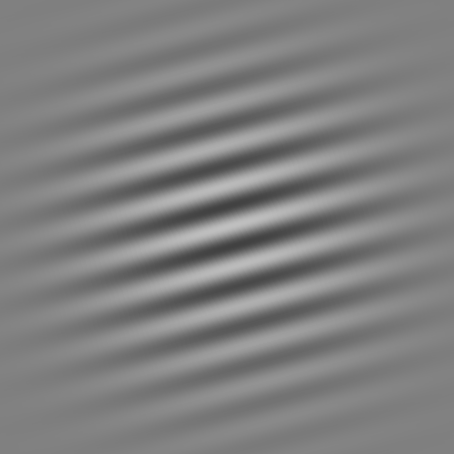

Supplement: S2 Data — (ZIP) [file pcbi.1014553.s010.zip › Curriculum-VPL-main/model_code/StimulusImages/SG_train_double_sf/sep_75.0/TARGET_ref_0_sep_75.0_contr_1_ph_0.0_sf_0.05_CW.png]

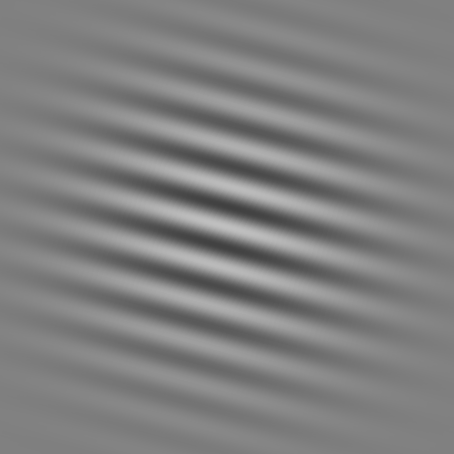

Supplement: S2 Data — (ZIP) [file pcbi.1014553.s010.zip › Curriculum-VPL-main/model_code/StimulusImages/SG_train_double_sf/sep_75.0/TARGET_ref_0_sep_75.0_contr_1_ph_0.0_sf_0.05_CCW.png]
