# Supplementary material for: The curriculum effect in visual learning: The role of readout dimensionality
Source: PLoS Comput Biol. 2026 Jul 24;22(7):e1014553. doi: 10.1371/journal.pcbi.1014553 (PMC13432104; doi:10.1371/journal.pcbi.1014553)
Supplement: Attachment — Submitted filename: Response to Reviewers.pdf [file pcbi.1014553.s011.pdf]

Responses to Reviewers

The curriculum effect in visual learning: the role of readout dimensionality

General response:

We sincerely thank the reviewers for their insightful comments and their overall support of our work. The manuscript has been significantly strengthened by incorporating the constructive feedback provided. In our responses to individual reviewer comments, the reviewer comments are shown in italicized black text. Our responses follow each comment in standard blue text. New additions/modifications to the manuscript

Below is a summary of the key revisions made to address the reviewers' comments:

- To better align the models with human behavioral data, we added results from a transfer test involving changes to the reference angle. This protocol more closely mirrors the tasks used in human experiments; we confirm that our original findings remain consistent under this new condition.

- We performed additional analyses on the functional properties of the most influential neurons selected during training in each training protocol. Using subspace similarity and orientation tuning analyses, we demonstrate that the sequential training paradigm selectively recruits neurons with the highest orientation selectivity, which are the most task-relevant.

- We included other model architectures to show that our results are not dependent on a specific pre-trained architecture (AlexNet).

- We reproduced our main findings using random initialization for the readout weights, demonstrating that our results are robust to the choice of initialization.

- We added more clear explanations on our choice of curriculum metric, and highlighted the existing technical trade-offs in measuring implicit curriculum.

- We validated our dimensionality analysis using an ablation-based technique to measure neuronal influence on model output, rather than relying solely on weight magnitude. This confirms that our subspace dimensionality results hold regardless of the metric used to quantify neuronal impact.

- We computed the gradient of the weight changes in addition to the L2 norms to ensure that different scales/number of parameters did not affect the comparison between readout/convolutional weight changes.

Reviewer #1: *In this paper the authors show significant evidence that readout dimensionality relates to transfer learning performance and that this may explain the impacts of curriculum order on perceptual learning. While I generally find the argument compelling, I have some questions about methods and results.*

We thank the reviewer for their support of our work and for the insightful comments they provided. We have addressed their specific points and questions in detail below

Methods Questions:
Regarding the transfer task for the model: why do the authors test transfer across spatial frequency in the model when this is not what is done in the humans? I understand the point about not testing spatial transfer in a CNN but why not also use a different reference orientation for the transfer test rather than different spatial frequency? Having the transfer test be of the same dimension as the learning itself (i.e. changing the reference orientation in an orientation judgement task) seems perhaps significantly different than performing an unrelated transfer task (i.e. changing spatial frequency in an orientation judgement task).

This is a very important point and also a great suggestion. We now provide analysis results on transfer testing on reference angle orientation for more comparable results to those of human experiments. **Fig. S2** shows the results in the revised manuscript. The results confirm our previous findings that transfer accuracy is negatively correlated with effective rank.

Added text:

- Lines 131-132 - “**Separately, we also evaluated generalization for a different reference angle orientation to confirm the robustness of our results (Fig. S2).**”
- Lines 717-719 - **“For the additional experiments in Fig. S2, we used a reference stimulus with 0° orientation for the train condition and a reference stimulus with 15° orientation for the transfer condition, with a constant SF of 0.05.”**

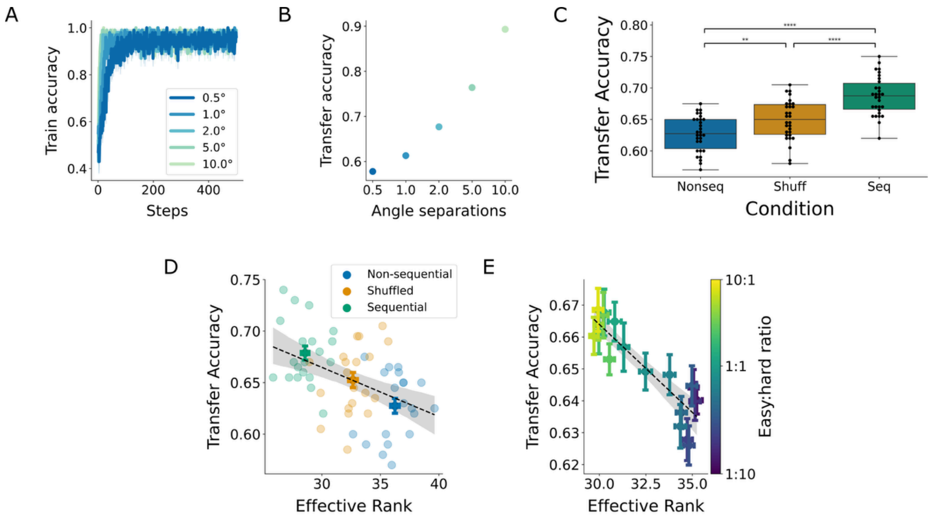

Fig. S2. Results for a changed reference orientation in the transfer condition. (A) Train accuracy. **(B)** Transfer accuracy. **(C)** Transfer accuracy for models across curricula. **(D)** Transfer accuracy versus effective rank for models across curricula (r=-0.45, p=0.00034). **(E)** Negative correlation of the effective rank for shuffled curricula with different easy:hard sample ratios with transfer accuracy (r=-0.89, p<<0.0001).

When training readouts, does every individual unit in a readout layer get its own weight or is there some kind of global pooling over feature maps such that weights are one per channel?

Yes, every individual unit gets its own weight. This allows the model to choose neurons with different receptive field locations, in addition to different feature selectivity.

These descriptions are inconsistent (I assume the first is correct):
228: "We used the mean of the absolute gradient values as a measure of the learning signal that drives the changes in a model's performance."
744: "the absolute value of the mean was taken over all the gradients of the model's training loss with respect to the model weights for the full model."

Thank you for catching this error. The first description is correct, the second description has been updated to match.

Modified text:

- Line 848-849: "To calculate the learning signal in the model, the **mean was taken over all the absolute gradient values** of the model's training loss with respect to the model weights for the full model."

The authors use "mean accuracy on the first n easy trials minus the mean accuracy on the first n hard trials [as] a proxy measure of each observer's implicit curriculum". But as n goes to zero this clearly is just a measure of the definition of hard versus easy trials (i.e. before training there will be a large difference between hard and easy trials). It seems like a measure of implicit curriculum would need to include some measure of the rate of change of learning for easy versus hard trials. The authors go on to note that their measure indeed does not predict learning for small n, and only does so for medium n (by including later trials the difference in learning rate is more detectable). I'd like more clarity on why the authors chose this metric to begin with.

Of course, we agree with the reviewer that our metric isn't a direct measure of curriculum. We chose the current metric to deal with certain technical limitations:

- Ideally, we shouldn't average trials, but noise in each trial is working against our ideal scenario, therefore we have to average n trials.
- Rate of change could be calculated, but once again, calculating difference between consecutive trials adds to high frequency noise.
- The fact that our metric doesn't show predictability for small n values is exactly because of the noise in individual trials that need to be reduced via looking at multiple trials at the same time.
- Because of the noisy nature of the data, looking at individual trials, or rate of changes between individual trials, is a serious technical challenge. A model based approach could potentially solve this problem (e.g., fitting a parametric or non-parameteric model to trial-based data) but that would also have some smoothing effect, perhaps similar to our averaging. More advanced techniques could be effective that we will develop for future works.
- We clarify that this is not a direct measure of curriculum in the following places, when we define the term:
    - Line 243-245: "We defined a "curriculum metric" (see Eq. 1 in Methods) as the mean accuracy on the first *n* easy trials minus the mean accuracy on the first *n* hard trials, providing a proxy measure of each observer's implicit curriculum."
    - Line 285-287: "Our curriculum metric provided a proxy measure for the strength of the easy-to-hard implicit curriculum that the observer followed in the first *n* easy trials and the first *n* hard trials."

- We have added some explanation in the methods section in the revised manuscript to further explain our choice of metric. We have also included a discussion of our choice of metric in the limitations section.

Added text:

- Lines 685-691: "**One other limitation of our analysis is our choice of curriculum metric. In this work, we used the difference between the mean accuracy on the first *n* easy trials and the mean accuracy on the first *n* hard trials as a proxy for the observers' implicit curricula. While this metric isn't a direct measure of curriculum, we chose it as a proxy in order to deal with the inherent noise present in the observer accuracy data. A rate of change metric would better approximate the actual implicit curriculum, as would a trial-by-trial metric instead of averaging over *n* trials, but due to the noisiness of the data these are technically infeasible. In future work, more advanced techniques for measuring the strength of the implicit curriculum could be effective.**"
- Lines 843-846: "**We chose to use this metric instead of, e.g., a rate of change metric, because calculating the difference in rate of change between consecutive trials considerably increased the amount of noise in the data. Due to the high amount of noise between individual trials, we computed the metric over the first *n* easy/hard trials instead of computing the metric trial-by-trial.**"

The authors say "For Fig. 4C, neurons were lesioned incrementally 10 at a time based on the absolute weight value, i.e. first the largest 10 weights were lesioned, then the largest 20 weights, etc". Are the neurons actually lesioned or just their weights to the readout? I.e. can the neural activity still propagate to the next layer in the network or not?

The weights in the readout itself are lesioned. In our model, we added skip connections from every layer of the model directly to the readout layer. We lesioned only these readout weights, i.e. the connection from each neuron directly to the output. So yes, the neural activity from each intermediate layer can still propagate to the next, all the way to the model output. To improve clarity, we have modified our explanation.

Modified text:

- Lines 317-319: "To identify these "influential" neurons, we lesioned the neurons with the largest absolute **readout** weights in increments **by removing their weights to the readout** and quantified the effect of **this** lesioning on transfer accuracy."
- Lines 873-876: "For Fig. 4C, neurons were lesioned incrementally 10 at a time **by setting their readout weights to zero. This removes the connection from the neuron to the readout. Lesioning was** based on the absolute **readout** weight value, i.e. first the largest 10 **readout** weights were lesioned, then the largest 20 **readout** weights, etc. Transfer accuracy was measured as usual after each set of neurons were lesioned."

How was figure 5B made? The methods say "PCA was calculated on the final activations of the 150-neuron readout subspace for a representative model". But aren't these 150 neuron subspaces different for each style of learning? Does this mean that the PCs are actually different for each different color of dot and therefore the axes are not actually shared?

Good point. PCA was calculated on all of the activations for each model together, so the axes are shared. Each set of activations was then coloured by the model they came from. This has been clarified in the methods section.

Added text:

- Lines 866-871: "For Fig. 6B, PCA was calculated on the final activations of the 150-neuron readout subspace for representative models **from each curriculum. The weights or activations from each model were concatenated before PCA and then coloured by the model they originated from after PCA. For both PCA figures, we used the StandardScaler from SciKit-Learn to standardize the data to zero mean and unit variance before computing the top two principal components.**"

Results Questions:
The authors largely focus on transfer accuracy when reporting results, but it would be helpful, to contextualize those results, to know about any differences in accuracy on the trained task for these same models. For example, do the models that have worse transfer performance have higher performance on the trained task?

Great questions. All our models reach near-perfect performance (~100% accuracy) during training, with no difference between angle separations (refer to Fig. 1D). Therefore, even though transfer accuracies are significantly different across angle separations, there is no significant difference between training accuracies across conditions.

The authors should plot the same behavioral analysis as done for humans in 3A for the models, in addition to the weight analysis as it is the more direct comparison.

Thanks for the suggestions. We agree with the reviewer that showing the same analysis on the human data and the models is informative. We have now added the suggested analysis to the paper. See **Fig. S4B** and the added explanation in the text.

Added text:

- Lines 233-235: "In the shuffled curriculum, parameter gradients peaked earlier for easy samples, and later for hard samples (Fig. 3B, **S4A**). **We observed a similar pattern in the accuracy of each condition (Fig. S4B)"**.

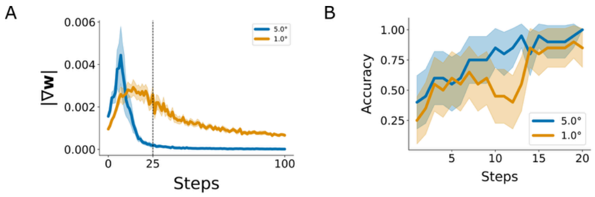

Fig. S4. Additional data supporting an implicit curriculum in the models. (A) Parameter gradients for models trained on the shuffled task condition in the first 100 steps of training. This plot includes gradients for the non-active condition at each timestep. **(B)** Accuracy throughout training for models on the shuffled condition shown separately for easy (5°) and hard (1°) samples.

The authors focus on the finding of how much readout dimensionality can predict transfer learning, but

they say less about what features are actually being represented or used. Of course low dimensionality alone is not sufficient to induce good performance if the wrong dimensions are chosen. When the authors test if enforcing low-dimensional readout during training on hard tasks leads to better transfer performance, they restrict learning to the neurons already known to contain the needed information, so this still conflates low-D representations with also using the correct features. I would, in particular, be interested to know if the models trained on hard-only tasks are using a different set of features than the low-D models or are instead using the same low-D set of features plus many more. In the first case, poor transfer is explained by having mismatched features but in the second it would be explained more by having extra features that contribute noise. These differences could also help predict the extent to which one can recover from poor initial training (e.g. do you need to completely relearn or just prune the extra, noisy dimensions). As far as I can tell the current analyses don't speak to this, but given that all models are based on the same AlexNet it would be potentially straightforward to determine if the same neurons are in the top 150 for different learning approaches. I wouldn't say such an analysis is technically required to support the claims of the paper, but I do feel like a part of the story is missing here.

We thank the reviewer for this important suggestion, which prompted an interesting new analysis. Motivated with this suggestion, we investigated the top 150 neurons used by each model. We ran two types of analysis on these most influential neurons: 1) subspace similarity analysis between the models (**Fig. 5A-B**); and 2) orientation tuning analysis on the neurons in the readout subspace (**Fig 5C-E**). Both analyses showed that there was a difference between the tuning properties of the influential neurons depending on the training protocol (sequential, shuffled, non-sequential). The subspace similarity analysis showed that the readout subspaces contain some similar features, with the shuffled and non-sequential curricula leading to the most similar readout subspaces. The orientation tuning analysis showed that neurons that have the largest influence in the sequential model were highly orientation selective (measured using Orientation Selectivity Index (OSI)) compared to the two other models. The shuffled model neurons were also more orientation selective than the non-sequential model. Also, the 150 readout subspace neurons in the sequential and shuffled models had more consistent selectivity than the 150 neurons in the nonsequential readout subspace.

Added text:

- Lines 371-421: **added results section called "Lower-dimensional readout subspaces contain more orientation selective neurons"**
- Lines 890-902: **added methods section on Jaccard index**
- Lines 910-922: **added methods section on orientation tuning analysis**

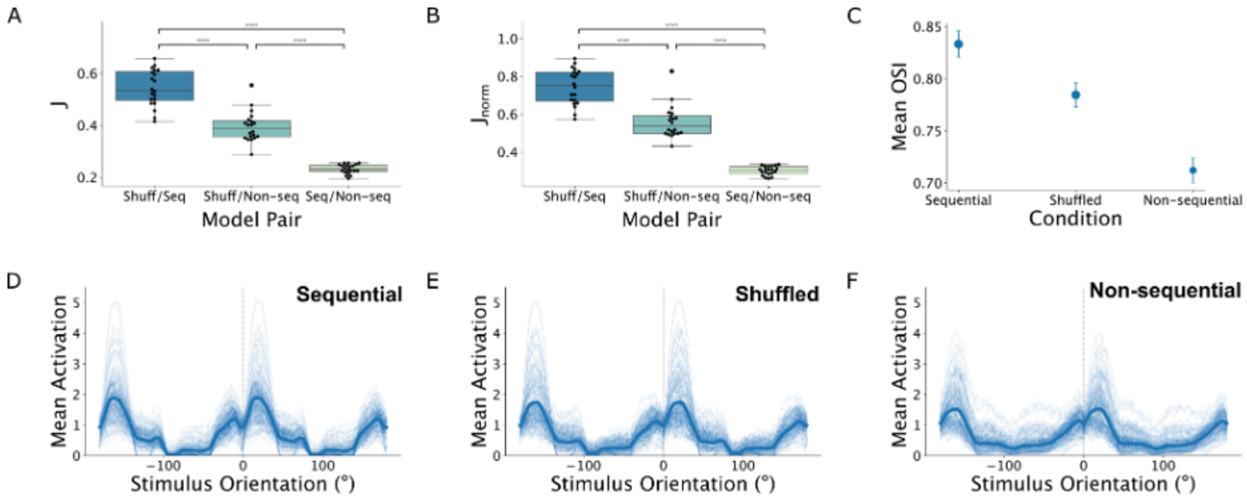

Fig. 5. Lower-dimensional readout subspaces contain highly orientation-selective neurons. (A) Jaccard index for the set of indices of neurons in the readout subspace compared across curricula. Models trained on shuffled or sequential curricula have the most neurons in common. (B) Jaccard index for the set of indices of neurons in the readout subspace compared across curricula, normalized by the Jaccard index between models trained on the same curriculum. (C) Mean orientation selectivity of the 150 neurons in the readout subspace, measured by the orientation selectivity index, for sequential, shuffled, and non-sequential curricula. Curricula with a lower-dimensional readout subspace (Fig. 4D) have a higher selectivity index. (D) Mean activations of each readout subspace neuron across stimulus orientations in the sequential curriculum. The mean across all neurons is shown as a thick line, individual neurons are shown as thin lines. (E) Same as (D) but in the shuffled curriculum. (F) Same as (D) but in the non-sequential curriculum. Both sequential and shuffled curricula have more consistent orientation selectivity across neurons than the non-sequential curriculum.

Reviewer #2: *The manuscript "The curriculum effect in visual learning: the role of readout dimensionality" by Volk et al. presents a compelling theoretical study of how readout dimensionality influences visual learning and transfer. The authors propose that easy-to-hard curricula promote the learning of low-dimensional solutions, which can then support improved transfer to harder tasks. The study offers an elegant explanation for curriculum learning effects in both artificial neural networks and human learners. Overall, I find the central claim convincing and the work conceptually valuable. That said, I have several methodological questions and points for clarification - If addressed, this would further strengthen the paper.*

We thank the reviewer for their overall support of our work and for their valuable comments. We have provided a point-by-point response to their feedback below.

Major comments

1. Interpretation of curriculum order and interleaving (line 58)
The manuscript states that "interleaving different task conditions can also enhance generalization," but the subsequent discussion emphasizes that task order matters. The cited literature does not clearly establish that order effects differ between interleaved and sequential training. It would be helpful if the authors could clarify whether they are suggesting that both interleaved and sequential curricula enhance

generalization, but that the effect is stronger for sequential curricula. If so, this claim may require additional justification or references.

This is an important point that warrants further clarification. Existing literature suggests that, on average, no significant difference exists between interleaved and sequential curricula across human observers; a finding consistent with our own data. However, a more nuanced picture emerges when examining inter-individual differences. Specifically, we observed substantially higher variability in the interleaved (shuffled) condition compared to the sequential condition. We showed that this difference could be explained by the adoption of an "implicit curriculum" by some participants. Those who showed a stronger implicit curriculum achieved superior generalization compared to the observers of the sequential condition, and those who did not show or showed weaker implicit curriculum generalized worse than the observers of the sequential condition. The average of the two groups of participants in the shuffled condition would end up close to the average of the sequential condition as shown in our data in Fig. 1B, consistent with previous literature (refs 75-83 in the revised manuscript). We also discussed this point in the discussion sections "Learning curriculum" and "Inter-individual variability in learning". To address the reviewer's valid concern, we have also added clarifying text in the introduction and in the discussion sections of the revised manuscript.

Added text:

- Lines 60-61 - "These findings suggest that the order and structure of training **can** substantially impact learning outcomes**, but it is unclear in which scenarios a sequential curriculum would be advantageous over an interleaved curriculum.**"
- Lines 577-580: "**This interpretation could help to reconcile the differing conclusions of previous literature on the benefits of a sequential curriculum (6–9). Averaging across participants using an interleaved curriculum removes the inter-individual variability, obscuring the conclusion that some participants may benefit from a shuffled curriculum while some benefit from an explicit sequential curriculum.**"

2. Human generalization across sequential and shuffled conditions (line 99)
The observation that humans show similar average generalization improvements in both sequential and shuffled conditions is described as "interesting." This result does seem unexpected given the authors' interpretation that order matters, but it is also consistent with the literature cited earlier (line 58) regarding interleaving benefits. A brief discussion reconciling these two interpretations would improve clarity.

We agree with the reviewer, as we also explained in response to the previous comment. Therefore, we have added some clarifying text to explain the differing conclusions from previous literature, and to explain how our results may help to explain the difference. We added this to the discussion section.

Added text:

- Lines 577-850: "**This interpretation could help to reconcile the differing conclusions of previous literature on the benefits of a sequential curriculum (6–9). Averaging across participants using an interleaved curriculum removes the inter-individual variability, obscuring the conclusion that some participants may benefit from a shuffled curriculum while some benefit from an explicit sequential curriculum.**"

3. Differences between human and model test sets (lines 125, 129)
Humans are tested on different angles at different spatial locations, whereas ANNs are tested on different

frequencies. While the authors provide reasonable arguments for these choices, it seems that testing both humans and models on a shared or more closely matched test dimension (e.g., different angles) would have been feasible. Using more comparable test sets could strengthen cross-system comparisons and help isolate whether observed differences arise from distinct learning strategies rather than task design. Perhaps the authors could elaborate a bit more why this was not done or perhaps it could still be done with a reasonable amount of effort.

We thank the reviewer for raising this crucial point. To ensure our computational results are more directly comparable to human behavioral data, we have performed an additional transfer test involving changes to the reference angle orientation and added some explanatory text.

As shown in **Fig. S2** of the revised manuscript, the results of this analysis are consistent with our original findings: transfer accuracy negatively correlates with effective rank. This confirms that the observed effects are robust even when using a transfer protocol that more closely mirrors the tasks employed in human experiments.

Added text:

- Lines 131-132 - "**Separately, we also evaluated generalization for a different reference angle orientation to confirm the robustness of our results.**"
- Lines 717-719 - **"For the additional experiments in Fig. S2, we used a reference stimulus with 0° orientation for the train condition and a reference stimulus with 15° orientation for the transfer condition, with a constant SF of 0.05."**

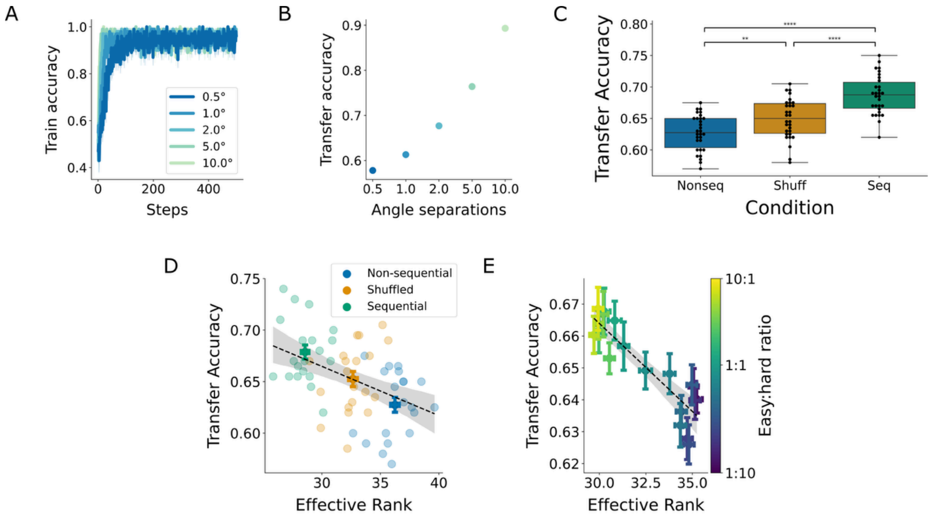

Fig. S2. Results for a changed reference orientation in the transfer condition. (A) Train accuracy. **(B)** Transfer accuracy. **(C)** Transfer accuracy for models across curricula. **(D)** Transfer accuracy versus effective rank for models across curricula (r=-0.45, p=0.00034). **(E)** Negative correlation of the effective rank for shuffled curricula with different easy:hard sample ratios with transfer accuracy (r=-0.89, p<<0.0001).

4. Initialization of readout weights and learning dynamics (lines 156, 646)
The authors argue that learning in the models occurs predominantly through changes in readout weights. However, since the readout weights are initialized to zero and the models are trained via

backpropagation, earlier layers cannot receive gradient updates until the readout weights become non-zero. This setup may introduce a bias toward readout-driven learning early in training. To rule out this possibility, it would be informative to repeat key analyses with randomly initialized readout weights matched in scale to other network weights.

We repeated our main results with randomly initialized readout weights to confirm that our findings hold with non-zero readout weight initialization. See **Fig. S3**, particularly plots C-D. Although the difference between the scales of learning in the readout vs. convolutional weights is slightly smaller in this case, the learning still occurs predominantly in the readout weights. The rest of our results hold as well.

Added text:

- Lines 728-730: "**To confirm that zero initialization does not affect the main conclusions, we repeated the main experiments with a randomly initialized readout weight vector (Fig. S3).**"

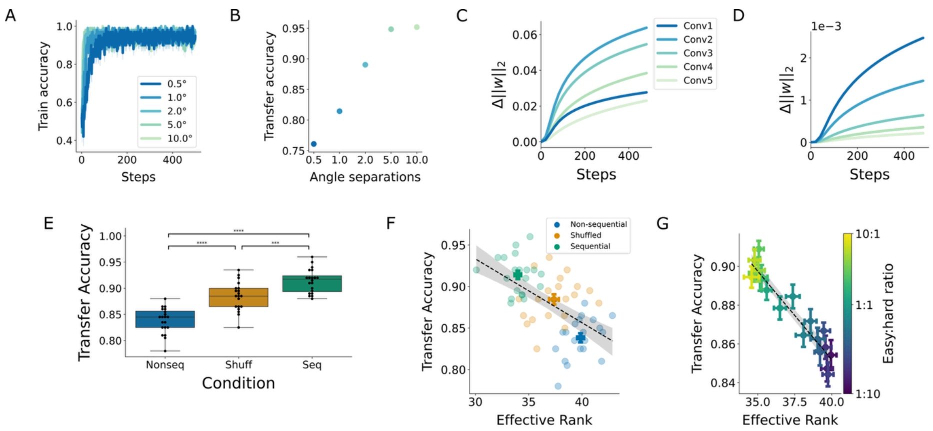

Fig. S3. Results for models with randomly initialized readout weights. (A) Train accuracy. **(B)** Transfer accuracy. **(C)** Change in the L2-norm of skip connection weights throughout training on a 0.5° angle separation. **(D)** Change in the L2-norm of convolutional weights throughout training on a 0.5° angle separation. **(E)** Transfer accuracy for models across curricula. **(F)** Transfer accuracy versus effective rank for models across curricula (r=-0.57, p<<0.0001). **(G)** Negative correlation of the effective rank for shuffled curricula with different easy:hard sample ratios with transfer accuracy (r=-0.95, p<<0.0001).

5. Identifying influential neurons via readout weights vs. functional contribution
To support the claim that readout subspace dimensionality determines generalization, the authors identify influential neurons by lesioning units with the largest absolute readout weights and measuring the resulting drop in transfer accuracy (ll 310). While this approach is intuitive, large readout weights do not necessarily imply large functional importance for task performance or generalization. A more principled measure of neuron importance would be to assess each neuron's contribution to training or validation loss, for example, by quantifying the increase in loss when a neuron is dropped or masked as done in standard gradient-based attribution techniques. Such an analysis more directly captures functional relevance rather than reliance on weight magnitude alone. Incorporating (or at least discussing) this alternative lesioning criterion would strengthen the link between readout dimensionality and generalization.

We repeated our neuron selection using an ablation technique, measuring functional contribution of individual neurons. To determine the individual contribution, neurons were lesioned one at a time by setting them to zero, and then transfer accuracy was measured as usual. As shown in **Fig. S6**, the results

confirm our previous findings of a negative correlation between effective rank and transfer accuracy. We also repeated our analysis with 150 randomly chosen neurons to confirm that the dimensionality of the overall readout subspace was not responsible for the differences between curricula (see **Fig. 4E**).

Added text:

- Lines 322-324: “**We also repeated this analysis by choosing 150 neurons with the largest individual functional contribution to the transfer accuracy to confirm that our results are unaffected (Fig. S6)**.”
- Lines 877-880: “**To determine the functional contribution of individual neurons, every neuron in the readout was lesioned one at a time and the transfer accuracy was measured as usual. After determining the 150 neurons that contributed the most to the readout, Fig. S6A was created as described for Fig. 4C.**”
- Lines 885-887: “We selected 150 neurons from the readout of each model instance with the highest absolute weight values (**or highest functional contribution as described above**) and selected the activations from the models based on the indices of these neurons.”
- Lines 339-342: “**When we repeated this analysis for 150 randomly chosen neurons, we did not see a correlation between transfer accuracy and effective rank between curricula (*r*=0.25, *p*=0.058) (Fig. 4E). This confirms that the dimensionality of the readout subspace is what differs here, not the overall dimensionality of the readout.**”

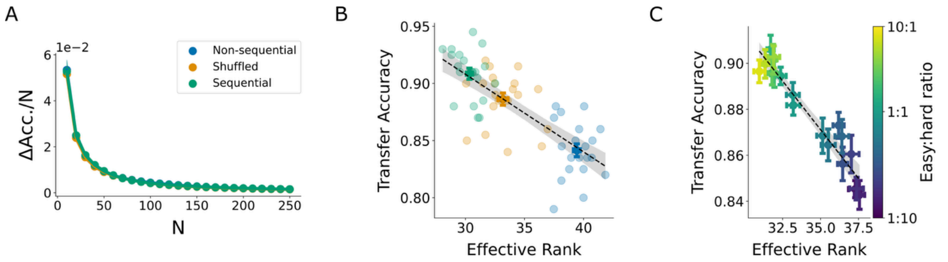

Fig. S6. Readout subspace dimensionality resulting from choosing 150 neurons with the largest contribution to the transfer accuracy. (A) Accuracy drop per neuron lesioned, plotted against the number of neurons lesioned. The neurons were chosen by their functional contribution to the transfer accuracy. The accuracy drop per neuron plateaued at about N=150, meaning that the readout subspace was mostly affected by about 150 neurons. **(B)** Transfer accuracy versus effective rank for models across curricula (*r*=-0.76, *p*<<0.0001). **(C)** Negative correlation of the effective rank for shuffled curricula with different easy:hard sample ratios with transfer accuracy (*r*=-0.96, *p*<<0.0001).

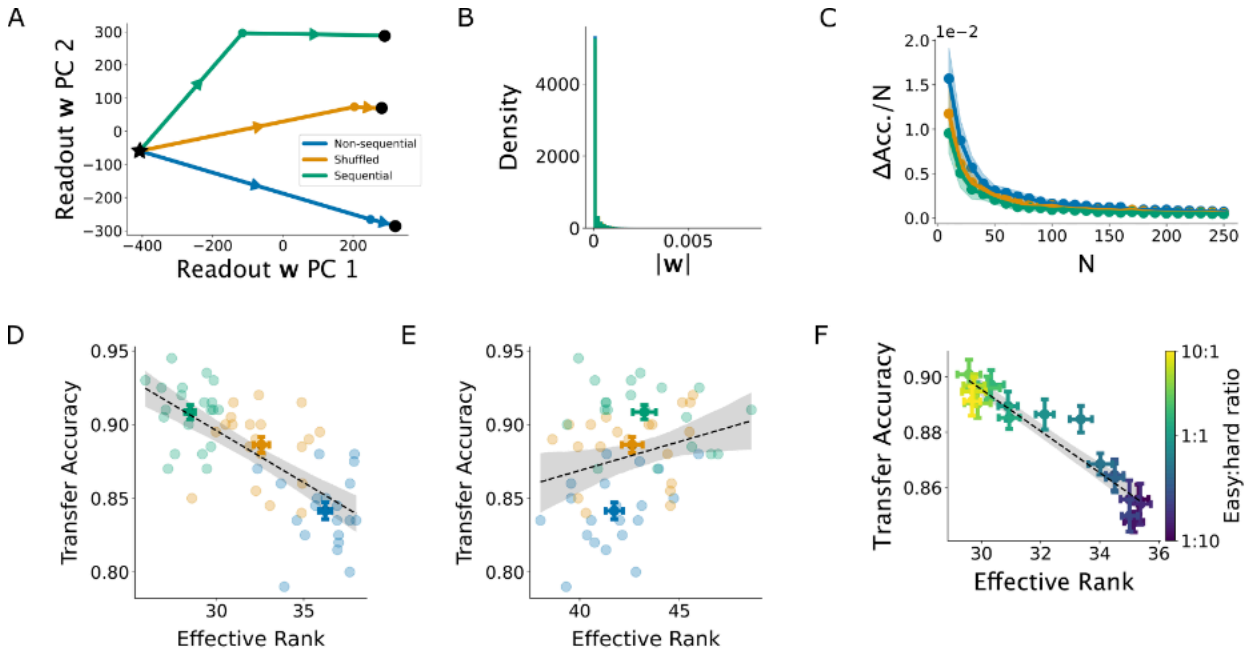

Figure 4. Readout subspace dimensionality determines generalization. (**A**) Projection of model readout weights onto their first two principal components for all three curricula at the beginning (marked by a black star), middle, and end (marked by black circles) of training. Arrows show the direction of training progression in weight space; different curricula follow different trajectories. (**B**) Histogram of all non-zero readout weights at the end of training. The majority of weights were zero, with only a small portion of non-zero weights. Distributions for the three curricula visually appear to overlap almost completely, and standard error is not visible. (**C**) Accuracy drop per neuron lesioned, plotted against the number of neurons lesioned. The accuracy drop per neuron plateaued at about N=150, meaning that the readout subspace was mostly affected by about 150 neurons. (**D**) Dimensionality of the 150-neuron readout subspace, measured by the effective rank, for non-sequential, sequential, and shuffled curricula (r=-0.69, p<<0.0001). Curricula leading to a low-dimensional readout subspace had a higher transfer accuracy. Results for other subspace sizes are shown in Fig. S2. **(E)** Dimensionality of the subspace created by sampling 150 random neurons from the readout. There is no apparent correlation in transfer accuracy and effective rank (r=0.25, p=0.058). (**F**) Negative correlation of the dimensionality of the readout subspace for shuffled curricula with different easy:hard sample ratios with transfer accuracy (r=-0.95, p<<0.0001). Models which were forced to learn from easy samples early in training had lower-dimensional readout subspaces and exhibited better generalization.

6. Correlation between effective rank and transfer accuracy (line 336, Fig. 4E)
The manuscript reports a strong positive correlation (r = 0.95) between effective rank and transfer accuracy. However, according to the stated hypothesis, this relationship should be negative, as is reported elsewhere (e.g., line 332) and as appears visually in Fig. 4E. It is possible that the reported correlation refers instead to the relationship between the easy:hard ratio and transfer accuracy, as described in the figure caption. Clarifying this point and, if necessary, correcting the text would avoid confusion.

Thanks for catching this error. This has been corrected in the revised manuscript.

Modified text:

- Line 109-111: "Learning generalization **had a strong negative correlation** with how many neurons with distinct visual feature preferences contributed more strongly to the readout…"
- Line 344-346: "We also measured the effective rank…finding a strong **negative** correlation between effective rank and transfer accuracy (*r*=**-**0.95, *p*<<0.0001)."
- Line 366-368 (Figure caption): "(**F**) **Negative** correlation of the dimensionality of the readout subspace…with transfer accuracy (*r*=**-**0.95, *p*<<0.0001)"

7. Performance when training only on the easy task (Fig. 5C)
In Fig. 5C, training exclusively on the easy task appears to yield higher mean transfer accuracy (~0.95) than either the sequential or shuffled curricula. This result is somewhat surprising given the emphasis on curriculum learning. A discussion of why training only on the easy task outperforms the proposed curricula, or whether this difference is statistically reliable, would be valuable.

The difference here is that the transfer condition for training only on 5° is also 5° with a doubled spatial frequency (like Fig. 1), unlike the sequential and shuffled curricula where we are testing the transfer on 1° with a doubled spatial frequency. For training only on 1°, the transfer condition is 1°. This explains the higher mean transfer accuracy when training only on the 5° angle separation. We have clarified this in the text in the methods section.

Added text:

- Lines 744-749: "For the results in Fig. 1, S1, S2A-B, and S3A-D, we trained 100 models each on a single angle separation (0.5°, 1°, 2°, 5°, or 10°) for 500 iterations each **and tested the transfer to the same angle separation. For the results in Fig. 6C and Fig. S7, we trained 20 models each on a single angle separation for 1000 iterations and tested the transfer to the same angle separation. For all three curricula (non-sequential, shuffled, and sequential) we tested the transfer on a 1° angle separation (i.e., the hard condition).**"

Minor comments and clarifications

- Terminology: implicit vs. explicit curricula (line 271)
The distinction between a "forced implicit curriculum" and an "explicit curriculum" is unclear, and the terminology appears somewhat inconsistent across the paper. A brief clarification or standardization of terms would improve readability.

Thank you for pointing this out. We have removed the term "forced implicit curriculum" and now just refer to it as a "forced curriculum" in the text to improve clarity.

Modified text:

- Line 275-277: "This suggests that forcing a "more sequential" **curriculum** in the shuffled condition improves generalization, and that the implicit curriculum that the model discovers through gradient descent-based learning (Fig. 3B) is perhaps sub-optimal for the task."
- Line 635-637: "This reinforces the benefits of our "forced" **quasi-sequential** curriculum in aiding the model to learn and reuse a low-dimensional readout subspace which it would not otherwise discover on its own."

- Reporting correlation strength and significance (line 332)
The phrase "negatively correlated" would benefit from a quantitative report of the correlation coefficient and its statistical significance.

Good point. We have added the correlation coefficient and statistical significance to the revised text.

Modified text:

- Lines 337-339: “We found that the effective ranks were negatively correlated with the transfer accuracies of the models…**(*r*=-0.69, *p*<<0.0001)**”

- Section references (line 377)
The manuscript refers to "Section 3.5," but the sections are not numbered. Please revise the reference to avoid confusion.

Thanks for catching this. We have revised this reference.

Modified text:

- Line 436-438: “To this end, we measured the activations…(see **Methods for further details**)...”

- Figure labeling (Fig. S1B)
The x-axis in Fig. S1B appears to be labeled "Angle separations," but, based on the description, it may be more accurately labeled "Steps."

Thanks for pointing this out. We have fixed the label, it now reads “**Steps**”.

Reviewer #3: *Overall the premise of the paper is interesting. The human data on learning curriculum is a strong part of the paper and the analysis on dimensionality changes with learning curriculum is novel. However, the major weakness lies in the modeling details. The experiments are conducted with a single convolutional ANN architecture, and it is not obvious that the results would generalize to other models. The discussion mentions that testing other model architectures is beyond the scope of this work, however, if the observed changes in representation due to learning are a common motif that will happen in any learning system, then it *should* replicate across other architectures. For this type of study, showing that the results generalize across models is critical – small changes such as learning rate choice, optimizer choice, initialization choice, etc. can have a large effect on the model representation. Given the VPL task and the small number of training steps required, training additional models to verify generalization seems feasible and would significantly strengthen the work. If the authors decide that additional models are infeasible for the study, then the language throughout the paper, including abstract, introduction, etc should be changed to avoid talking about “ANNs” generally and instead talk about the specific model (and architecture) that was used.*

We thank the reviewer for raising this crucial point. To address this limitation, we’ve added other ANN architectures in our analysis.

However, we should clarify that there is an important constraint in the model architectures that could be included in this study. The nature of the task requires fine orientation information preserved at the output of the model. In most of the pretrained convolutional architectures, especially the deeper models, due to spatial averaging that happens through the model, the fine orientation information required for the task diminishes at the output. Adding skip connections from every layer to the output could partially solve the

problem, but for larger models, this makes the training quite intractable without extensive hyper-parameter optimization.

Due to this crucial point, we selected a few other architectures that could still solve the task without changing the architecture and extensive hyper-parameter engineering and optimization. The results of the analysis including these extra models support our main claim that transfer accuracy negatively correlates with the dimensionality of the readout subspace, measured by effective rank (see **Appendix S1, Fig. S7**). See also the expanded limitations section in the discussion, where we address the use of other model backbones.

Added text:

- Lines 693-706: "A final limitation of our work is the pre-trained ANN architecture that we used in our modeling experiments. AlexNet (21) has often been used to model the ventral visual pathway in previous work on VPL (17,90,91) and has been shown to perform similarly to newer and more complex architectures in terms of model-brain alignment (24). Further justification of our choice of model was presented in the results section. However, AlexNet and similar architectures remain rough approximations of visual system architecture and connectivity. For example, feedforward networks like AlexNet lack the lateral or feedback recurrent connections present in biological systems (91). Some have argued that models combining feedforward and recurrent connections are more plausible models of VPL than AlexNet or other strictly feedforward ANNs (91, 92). **In an effort to address this limitation, we investigated several other model backbones (see Appendix S1, Fig. S7). Although the correlation between readout subspace dimensionality and generalization holds for some models, it is not universal across all backbone architectures. This variability indicates that the relationship between readout dimensionality and generalization may interact with specific architectural properties. Further research is required to characterize how different architectural inductive biases influence the emergence of this dimensionality-generalization relationship.**"
- Lines 730-735: "**For the results in Fig. S7, we substituted other model backbones for the AlexNet architecture and added skip connections from every layer of the model directly to a single readout layer. We initialized the readout layers randomly for these experiments and froze the model backbones, allowing only the readout layer to update. The results shown in Fig. S7 include the AlexNet, EfficientNet, and GoogLeNet backbones. We also tried more complex architectures: ConvNeXt, DenseNet121, MNASNet, MobileNetV1/V2, SqueezeNet, ResNet18, and VGG11/16.**"

Appendix S1. Results for different model backbones

To investigate the robustness of our results across different model backbone choices, we replaced our AlexNet backbone with two other ANN model architectures: GoogLeNet (1) and EfficientNet (2) with added skip connections from each layer to the readout. These models were selected because they could solve the task without extensive hyperparameter engineering and optimization. Additionally, like AlexNet, these models are small convolutional backbones without complex information pathways or spatial averaging. We found that these models show similar patterns of transfer accuracy versus task difficulty, and that the transfer accuracy is negatively correlated with the dimensionality of the readout subspace (Fig. S7; r=-0.89, p=0.046). However, our results did not extend to other larger and more complex model backbones (see Methods for a complete list of architectures), which are less accurate approximations of visual system architecture and connectivity (24).

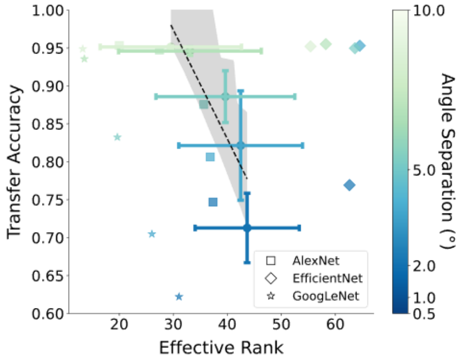

Fig. S7. Comparing readout subspace dimensionality in different model backbones. Transfer accuracy versus effective rank for selected model backbones (AlexNet, EfficientNet, and GoogLeNet) trained and tested on single angle separations. Correlation is calculated based on the means across all models for single angle separation (*r*=-0.89, *p*=0.046).

128-131: The difference between the types of generalization that are tested in humans and models seems like a confound if the goal of the paper is to directly compare generalization in humans and machines. This is especially concerning given that the reason given is variability across observers, but later on observer variability is highlighted as an interesting feature of the learning curriculum.

Very good point. We now provide analysis results on transfer testing on reference angle orientation for more comparable results to those of human observers. **Fig. S2** shows the results in the revised manuscript. The results confirm our previous findings, namely, that transfer accuracy negatively correlates with effective rank.

Added text:

- Lines 131-132 - "**Separately, we also evaluated generalization for a different reference angle orientation to confirm the robustness of our results.**"
- Lines 717-719 - "**For the additional experiments in Fig. S2, we used a reference stimulus with 0° orientation for the train condition and a reference stimulus with 15° orientation for the transfer condition, with a constant SF of 0.05.**"

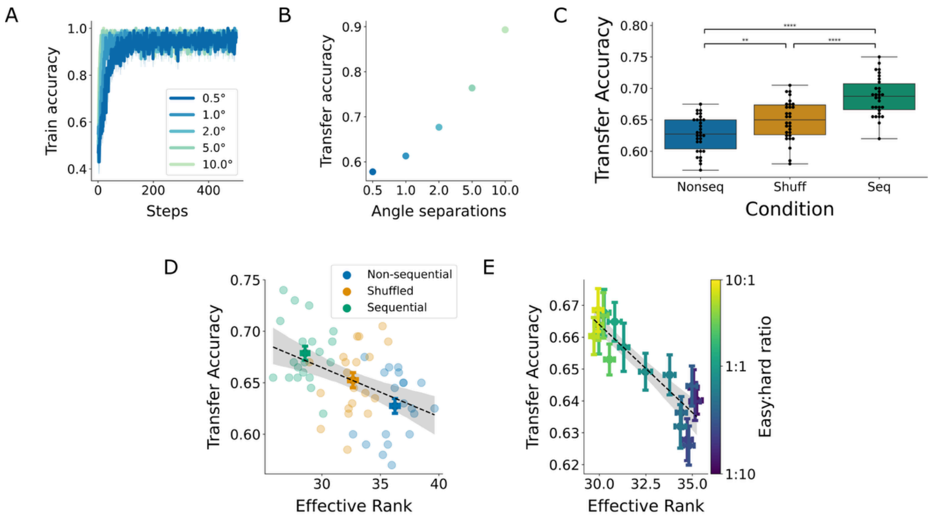

Fig. S2. Results for a changed reference orientation in the transfer condition. (A) Train accuracy. **(B)** Transfer accuracy. **(C)** Transfer accuracy for models across curricula. **(D)** Transfer accuracy versus effective rank for models across curricula (*r*=-0.45, *p*=0.00034). **(E)** Negative correlation of the effective rank for shuffled curricula with different easy:hard sample ratios with transfer accuracy (*r*=-0.89, *p*<<0.0001).

127: "convolutional ANNs are equivariant to spatial location" – as far as I can tell from the methods, this is incorrect for the model architecture used for the following reasons: (1) with the described AlexNet-style architecture, the final stage before the fully connected readout layer maintains some spatial information, so the actual readout will not be equivariant to location (2) typical boundary handling operations, like padding, break perfect shift-equivariance (3) operations such as strided convolutions and max pooling result in non-equivariance to small transformations (see https://arxiv.org/pdf/1904.11486 & https://arxiv.org/abs/1805.12177). I understand that this is not the focus of the paper and the text describes what seems like a somewhat minor modeling choice, however given that this is a study primarily about modeling, it is critical that the description of the ANN properties and training setups is technically precise.

Thank you for the comment. To reflect any uncertainty about equivariance/invariance in CNNs for different operations, we changed the phrasing of this line.

Modified text:

- Lines 128-130: "However, testing on a different location in the visual field **may** not cause specificity in convolutional ANNs, as they **may be** equivariant to spatial location."

154-156: I am concerned that the change in the L2 norms of the weights may be confounded by underlying different scales for the feed-forward convolutional layer weights and the skip connection weights, or potentially the number of parameters that go into the L2-norm. It might be better to normalize the change in weight magnitude by something like the final weight norm. Additionally, how does the learning change if the skip connection weights are initialized at different values other than exactly zero? It is a bit non-standard in deep learning to initialize to exactly zero (small non-zero values can help break symmetries and prevent the network from getting "stuck") so I was a bit surprised by this choice.

Great points raised by the reviewer. To address the questions:

- Since normalizing by the final weight norm would result in all of the changes in weight magnitude ranging from 0 to 1, we computed the gradient of the weight changes to address the confound of underlying different scales/number of parameters (see **Fig. S1D-E**).
- The choice of zero initialization was used previously in Wenliang and Seitz (2018) and we followed the same methodology. We repeated our main results with randomly initialized readout weights to confirm that our findings hold with non-zero readout weight initialization. We confirm that the negative correlation of transfer accuracy with effective rank holds. See **Fig. S3**.

Modified text:

- Lines 157-159: "Our analysis showed that the changes in the L2-norm of the weights in the skip connections (Fig. 1F, **S3C**) **or in the gradients of the weight norms (Fig. S1D)** were approximately 200x larger than those in the between-layer convolutional weights (Fig. 1G, **S3D, S1E**)."
- Lines 728-730: "**To confirm that zero initialization does not affect the main conclusions, we repeated the main experiments with a randomly initialized readout weight vector (Fig. S3).**"

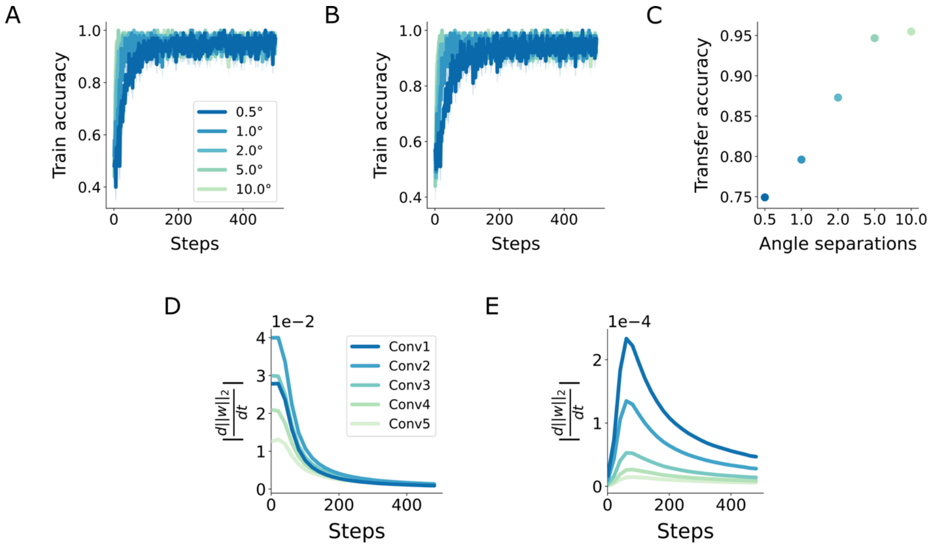

Fig. S1. Additional training, transfer accuracy, and weight change data. (**A**) Train accuracy with standard error visible. (**B**) Train accuracy with standard error visible for a model with convolutional and pooling layer weights frozen and readout weights unfrozen. (**C**) Transfer accuracy for the model from B. **(D)** Gradient of the weight norm of skip connection weights throughout training on a 0.5° angle separation. **(E)** Gradient of the weight norm of skip connection weights throughout training on a 0.5° angle separation.

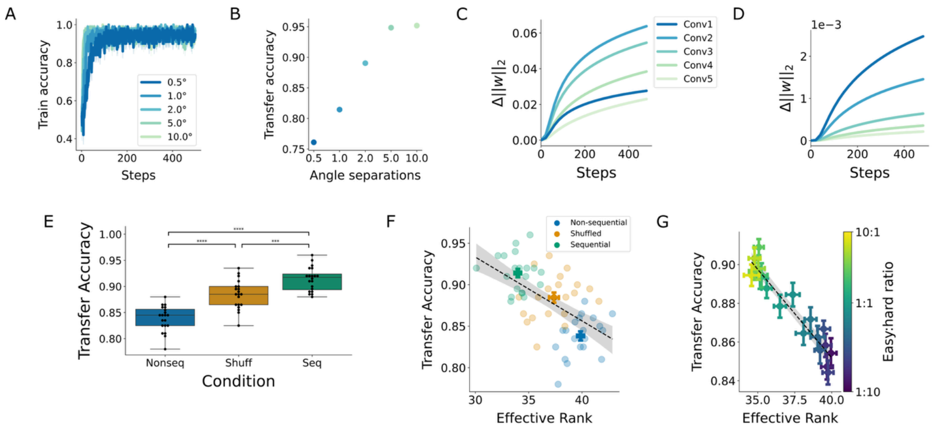

Fig. S3. Results for models with randomly initialized readout weights. (A) Train accuracy. **(B)** Transfer accuracy. **(C)** Change in the L2-norm of skip connection weights throughout training on a 0.5° angle separation. **(D)** Change in the L2-norm of convolutional weights throughout training on a 0.5° angle separation. **(E)** Transfer accuracy for models across curricula. **(F)** Transfer accuracy versus effective rank for models across curricula (r=-0.57, p<<0.0001). **(G)** Negative correlation of the effective rank for shuffled curricula with different easy:hard sample ratios with transfer accuracy (r=-0.95, p<<0.0001).

207: Please describe how multiple points for the model results are obtained (I believe that each point corresponds to a different random trial order and different random noise applied during training, but I could not find this detail). Going back to my request at the beginning of the paper regarding different architectures, the results would be much stronger if there were multiple architectures included in the results, for instance if each point were a different pre-trained neural network backbone (including transformers, different CNNs, etc).

Thank you for the comment. Yes, multiple points for the model results are obtained by varying the random noise added to each stimulus and confidence estimate across trials, and by randomizing the order of the 5° and 1° conditions in the shuffled curriculum for each participant and model instance. The procedure for obtaining multiple model points has been clarified in the revised text. To address the point about different model architectures, see **Fig. S7** for a similar plot with AlexNet, EfficientNet, and GoogLeNet backbones. We discussed this in more detail above in response to a previous point by the reviewer.

Modified text:

- Line 740: "**We applied this noise independently for each trial.**"
- Lines 811-812: "**For the shuffled condition, the order of the 5° and 1° trials was randomized for each participant/model instance.**"
- Lines 885-887: "We selected 150 neurons from the readout of each model **instance** with the highest absolute weight values (or highest functional contribution as described above) and selected the activations from the models based on the indices of these neurons.

300: Additional details on how PCA was performed should be given in the methods. Was any whitening performed on the weights? Were the weights flattened? How were the weights from different readout layers combined?

Thank you for pointing this out. Yes, the PCA used the StandardScaler from SciKit-Learn, which standardizes the data to zero mean and unit variance. The weights were not flattened because they originate from a linear readout layer. The weights from different readout layers were combined by concatenating before PCA. These details have been added to the methods

Added text:

- Lines 865-871: “For Fig. 4A, PCA was calculated on the full readout weight vectors taken at the beginning of training, halfway through training, and at the end of training. For Fig. 6B, PCA was calculated on the final activations of the 150-neuron readout subspace for **representative models from each curriculum. The weights or activations from each model’s readout subspace were concatenated before PCA and then coloured by the model they originated from after PCA. For both PCA figures, we used the StandardScaler from SciKit-Learn to standardize the data to zero mean and unit variance before computing the top two principal components.**”

315, Question: Are the ~150 neurons the same for each of the model instances that are trained, or is a different subset of 150 neurons selected?

The subsets of 150 neurons may be slightly different across model instances, as they were selected based on the weight/contribution of the readout neurons for that specific model instance. We have clarified this in the methods.

Modified text:

- Lines 885-887: “We selected 150 neurons from the readout of each model **instance** with the highest absolute weight values (or highest functional contribution as described above) and selected the activations from the models based on the indices of these neurons.”

*746-749: Minor: Even though only one type of trial is used for training, the gradient magnitudes for the other conditions could still be *measured*, but not used for updates.*

Yes, good point. We have added this to the revised manuscript, see **Fig. S4A**.

Added text:

- Lines 850-854: “**For Fig. 3B**, because only one difficulty condition occurs on each step of training, gradient magnitudes of the other condition were set to the previous value of that condition, or to zero if no previous value existed. For example, on a 1° trial, the gradient magnitude for plotting the 5° condition was set to the value calculated in the last 5° trial. **For Fig. S4A, we plotted both the active and non-active gradients at each timestep.”**

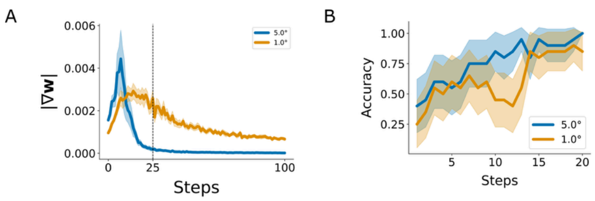

Fig. S4. Additional data supporting an implicit curriculum in the models. (A) Parameter gradients for models trained on the shuffled task condition in the first 100 steps of training. This plot includes gradients for the non-active condition at each timestep. **(B)** Accuracy throughout training for models on the shuffled condition shown separately for easy (5°) and hard (1°) samples.

Line 71 & 109 -- Question: How is a "distinct visual feature" (line 71) or a "dissimilar visual feature" (line 109) defined for the paper? The definition of a "feature" vs. "feature combination" seems important, and from the paper I'm not quite sure what the authors mean.

Thank you for the comment. We have not investigated exactly which visual features may be included in the solutions of these models, but some examples. are listed in lines 74-78: "For example, in learning to discriminate between rotated images, easy training (large rotation differences) might lead the visual system to discover that edge orientation alone is sufficient for discrimination. However, hard training (small rotation differences) might recruit more additional features (e.g., texture patterns, local contrast, multiple orientation channels, etc.) to achieve higher accuracy.". By "distinct visual feature" or "dissimilar visual feature", we mean that each distinct visual feature would have some unique characteristic. For example, the visual feature of a bar orientation would be distinct/dissimilar from the visual feature of local contrast, or spatial frequency. A feature combination might be a group of several neurons that respond to a set of these types of visual features. We have changed "dissimilar visual feature" to "distinct visual feature" throughout the text to maintain consistency, and added some clarifying text.

Modified text:

- Line 109-112: "Learning generalization had a strong negative correlation with how many neurons with **distinct** visual feature preferences contributed more strongly to the readout, with networks using fewer visual features, **or lower-dimensional readouts,** showing better transfer to new conditions."
- Lines 493-495: "We showed that the dimensionality of readout subspaces (neurons with **distinct** visual feature preferences that contributed strongly to model behaviour) correlated with generalization capacity."

The authors may want to include a brief discussion on this recent work:
https://www.nature.com/articles/s41562-025-02359-3

Thank you for the reference, we have included it in the discussion.

Added text:

- Lines 668-670: "For example, recent work in ANNs has suggested that in both humans and ANNs, interleaving is advantageous during incremental learning and blocking is advantageous during rapid inference (84). **On the other hand, a recent study comparing transformers and ANNs on a rule-learning query response task found that humans benefit from observing many diverse examples at the beginning of training to promote in-context learning first, while ANNs do not benefit (86).**"

Have the authors made all data and (if applicable) computational code underlying the findings in their manuscript fully available?
The PLOS Data policy requires authors to make all data and code underlying the findings described in

their manuscript fully available without restriction, with rare exception (please refer to the Data Availability Statement in the manuscript PDF file). The data and code should be provided as part of the manuscript or its supporting information, or deposited to a public repository. For example, in addition to summary statistics, the data points behind means, medians and variance measures should be available. If there are restrictions on publicly sharing data or code —e.g. participant privacy or use of data from a third party—those must be specified.

Reviewer #1: None

Reviewer #2: ***No:*** *The authors wrote "All code necessary to reproduce the results are available at the following link for the initial submission: Link." but the "Link" is not clickable (although underlined). Perhaps this is a problem due to the journal's PDF conversion? It would be great if this worked in the final version or if the actual link was included in plain text. Same problem with the raw data.*

Thank you for pointing this out. We have included the full plain text links in the submission. We have also uploaded the data and code as supplementary material along with the submission. Here is our updated Data and materials availability statement with the included links (Lines 1224-1231):

Data and materials availability: All data are available in the main text, supplementary materials, and/or the locations specified in this section.
Raw data from human observers are available at the following link for the initial submission: https://drive.google.com/file/d/1ZrHiH7aOwNL4kyeyFmYVIfM5ZZTuwW6B/view?usp=sharing. Data will be made publicly available upon publication.
All code necessary to reproduce the results are available at the following link for the initial submission: https://drive.google.com/file/d/115FcLjiCbaP5nyXWNHwEIWmSLJ21K4aR/view?usp=sharing. Code will be made publicly available on Github upon publication and a permanent archive will be created.

Reviewer #3: None
