# Supplementary material for: The curriculum effect in visual learning: The role of readout dimensionality
Source: PLoS Comput Biol. 2026 Jul 24;22(7):e1014553. doi: 10.1371/journal.pcbi.1014553 (PMC13432104; doi:10.1371/journal.pcbi.1014553)
Supplement: Attachment — Submitted filename: Response to Reviewers - 2.pdf [file pcbi.1014553.s012.pdf]

Responses to Reviewers

The curriculum effect in visual learning: the role of readout dimensionality

General response:

We thank the reviewers for their comments. In our responses to individual reviewer comments, the reviewer comments are shown in italicized black text. Our responses follow each comment in standard blue text. New additions/modifications to the manuscript are bolded.

Below is a summary of the key revisions made to address the reviewers' comments:

- We have changed the wording in Appendix S1 to better reflect the relevance of one of the references
- We have added additional description of the model architectures we tried and criteria for their inclusion in the supplementary results in Appendix S1

Reviewer #1*: I appreciate the substantial work the authors put in to update the manuscript.*

Reviewer #2*: Thanks for addressing all points.*

Reviewer #3: *The revisions have significantly strengthened the work. I particularly appreciate the addition of the new model architectures, and the results with the reference angle seem much closer to the human behavioral work. It is nice to see that the results generalize in both cases. Overall, the revision has substantially improved the paper. I have two fairly minor suggestions about the architecture results:*

Appendix S1: > "However, our results did not extend to other larger and more complex model backbones (see Methods for a complete list of architectures), which are less accurate approximations of visual system architecture and connectivity (24)."

I'm not sure reference 24 is appropriate for this claim, as the paper is about privileged representational axes and not connectivity (and also has a wide range of tests for ResNet50 and ResNets are one of the architecture types that the authors reported as not generalizing).

Good point. We agree that our point didn't come across as clearly as it should, therefore we revised the text to better reflect the relevance of the reference in this context. We think that the similarity of single-neuron tuning properties between models and the brain is one of the key factors in the observed relationship between dimensionality and learning generalization (Fig. 5) and this reference explicitly shows more single-neuron alignment between AlexNet and the brain compared to models like ResNet. We have explained this hypothesis in the appendix text, but we would need to explicitly provide evidence for this in future work.

Added text:

- Appendix S1: However, our results did not extend to other larger and more complex model backbones (see Methods for a complete list of architectures), **which are less accurate approximations of single-neuron tuning properties in the visual system [3]. As we showed in Fig. 5, single-neuron tuning properties are key to the observed relationship between dimensionality and generalization, so we conjecture that the models with more similar single-neuron tunings to the brain should better reflect learning generalization in humans.**

Lines 739-740: > "We also tried more complex architectures: ConvNeXt, DenseNet121, MNASNet, MobileNetV1/V2, SqueezeNet, ResNet18, and VGG11/16."
It seems like an additional description of why these results weren't included would be appropriate, for instance including some of the wording that was given in the rebuttal response. Documenting what didn't work (and potential reasons why) seems important for reproducibility purposes and future work.

Agreed, thanks for the suggestion. We have added some of the wording from the previous response to reviewers in the Appendix S1, and refer readers to Appendix S1 in the main text.

Added text:

- Lines 735-736: The results shown in Fig. S7 include the AlexNet, EfficientNet, and GoogLeNet backbones. We also tried more complex architectures: ConvNeXt, DenseNet121, MNASNet, MobileNetV1/V2, SqueezeNet, ResNet18, and VGG11/16. **See Appendix S1 for details.**

- Appendix S1: However, our results did not extend to other larger and more complex model backbones (see Methods for a complete list of architectures), **which are less accurate approximations of single-neuron tuning properties in the visual system [3]. As we showed in Fig. 5, single-neuron tuning properties are key to the observed relationship between dimensionality and generalization, so we conjecture that the models with more similar single-neuron tunings to the brain should better reflect learning generalization in humans. Another potential reason for this discrepancy relates to the preserved fine orientation information required at the output of the model due to the nature of the task. In most of the pretrained convolutional architectures, particularly the deeper models, the fine orientation information diminishes at the output due to spatial averaging through the model. Adding skip connections from every layer to the output could partially solve this problem, but for larger models this makes training intractable without extensive engineering and hyperparameter optimization.**
