# Supplementary material for: The curriculum effect in visual learning: The role of readout dimensionality
Source: PLoS Comput Biol. 2026 Jul 24;22(7):e1014553. doi: 10.1371/journal.pcbi.1014553 (PMC13432104; doi:10.1371/journal.pcbi.1014553)
Supplement: Peer review history

# Peer review: Decision Letter 0

**DOI:** 10.1371/journal.pcbi.1014553.r001

**Authors:** Marieke Karlijn van Vugt, Tim Christian Kietzmann

8 Jan 2026

PCOMPBIOL-D-25-02227

The curriculum effect in visual learning: the role of readout dimensionality

PLOS Computational Biology

Dear Dr. Bakhtiari,

Thank you for submitting your manuscript to PLOS Computational Biology. After careful consideration, we feel that it has merit but does not fully meet PLOS Computational Biology's publication criteria as it currently stands. Therefore, we invite you to submit a revised version of the manuscript that addresses the points raised during the review process.

Please submit your revised manuscript by Mar 09 2026 11:59PM. If you will need more time than this to complete your revisions, please reply to this message or contact the journal office at ploscompbiol@plos.org. When you're ready to submit your revision, log on to https://www.editorialmanager.com/pcompbiol/ and select the 'Submissions Needing Revision' folder to locate your manuscript file.

Please include the following items when submitting your revised manuscript:

* A letter that responds to each point raised by the editor and reviewer(s). You should upload this letter as a separate file labeled 'Response to Reviewers'. This file does not need to include responses to formatting updates and technical items listed in the 'Journal Requirements' section below.

* A marked-up copy of your manuscript that highlights changes made to the original version. You should upload this as a separate file labeled 'Revised Manuscript with Track Changes'.

* An unmarked version of your revised paper without tracked changes. You should upload this as a separate file labeled 'Manuscript'.

If you would like to make changes to your financial disclosure, competing interests statement, or data availability statement, please make these updates within the submission form at the time of resubmission. Guidelines for resubmitting your figure files are available below the reviewer comments at the end of this letter

We look forward to receiving your revised manuscript.

Kind regards,

Tim Christian Kietzmann, Dr. rer. nat.

Academic Editor

PLOS Computational Biology

Marieke van Vugt

Section Editor

PLOS Computational Biology

**Journal Requirements:**

If the reviewer comments include a recommendation to cite specific previously published works, please review and evaluate these publications to determine whether they are relevant and should be cited. There is no requirement to cite these works unless the editor has indicated otherwise.

1) We ask that a manuscript source file is provided at Revision. Please upload your manuscript file as a .doc, .docx, .rtf or .tex. If you are providing a .tex file, please upload it under the item type u2018LaTeX Source Fileu2019 and leave your .pdf version as the item type u2018Manuscriptu2019.

2) Please upload all main figures as separate Figure files in .tif or .eps format. For more information about how to convert and format your figure files please see our guidelines:

https://journals.plos.org/ploscompbiol/s/figures

3) We notice that your supplementary Figures are included in the manuscript file. Please remove them and upload them with the file type 'Supporting Information'. Please ensure that each Supporting Information file has a legend listed in the manuscript after the references list.

4) Please amend your detailed Financial Disclosure statement. This is published with the article. It must therefore be completed in full sentences and contain the exact wording you wish to be published.

1) State what role the funders took in the study. If the funders had no role in your study, please state: "The funders had no role in study design, data collection and analysis, decision to publish, or preparation of the manuscript."

2) If any authors received a salary from any of your funders, please state which authors and which funders..

If you did not receive any funding for this study, please simply state: u201cThe authors received no specific funding for this work.u201d

**Reviewers' comments:**

Reviewer's Responses to Questions

**Comments to the Authors:**

**Please note here if the review is uploaded as an attachment.**

Reviewer #1: In this paper the authors show significant evidence that readout dimensionality relates to transfer learning performance and that this may explain the impacts of curriculum order on perceptual learning. While I generally find the argument compelling, I have some questions about methods and results.

Methods Questions:

Regarding the transfer task for the model: why do the authors test transfer across spatial frequency in the model when this is not what is done in the humans? I understand the point about not testing spatial transfer in a CNN but why not also use a different reference orientation for the transfer test rather than different spatial frequency? Having the transfer test be of the same dimension as the learning itself (i.e. changing the reference orientation in an orientation judgement task) seems perhaps significantly different than performing an unrelated transfer task (i.e. changing spatial frequency in an orientation judgement task).

When training readouts, does every individual unit in a readout layer get its own weight or is there some kind of global pooling over feature maps such that weights are one per channel?

These descriptions are inconsistent (I assume the first is correct):

228: "We used the mean of the absolute gradient values as a measure of the learning signal

that drives the changes in a model’s performance."

744: "the absolute value of the mean was taken over all the gradients

of the model’s training loss with respect to the model weights for the full model."

The authors use "mean accuracy on the first n easy trials minus the mean accuracy on the first n hard trials [as] a proxy measure of each observer’s implicit curriculum". But as n goes to zero this clearly is just a measure of the definition of hard versus easy trials (i.e. before training there will be a large difference between hard and easy trials). It seems like a measure of implicit curriculum would need to include some measure of the rate of change of learning for easy versus hard trials. The authors go on to note that their measure indeed does not predict learning for small n, and only does so for medium n (by including later trials the difference in learning rate is more detectable). I'd like more clarity on why the authors chose this metric to begin with.

The authors say "For Fig. 4C, neurons were lesioned incrementally 10 at a time based on the absolute weight value, i.e. first the largest 10 weights were lesioned, then the largest 20 weights, etc". Are the neurons actually lesioned or just their weights to the readout? I.e. can the neural activity still propagate to the next layer in the network or not?

How was figure 5B made? The methods say "PCA was calculated on the final activations

of the 150-neuron readout subspace for a representative model". But aren't these 150 neuron subspaces different for each style of learning? Does this mean that the PCs are actually different for each different color of dot and therefore the axes are not actually shared?

Results Questions:

The authors largely focus on transfer accuracy when reporting results, but it would be helpful, to contextualize those results, to know about any differences in accuracy on the trained task for these same models. For example, do the models that have worse transfer performance have higher performance on the trained task?

The authors should plot the same behavioral analysis as done for humans in 3A for the models, in addition to the weight analysis as it is the more direct comparison.

The authors focus on the finding of how much readout dimensionality can predict transfer learning, but they say less about what features are actually being represented or used. Of course low dimensionality alone is not sufficient to induce good performance if the wrong dimensions are chosen. When the authors test if enforcing low-dimensional readout during training on hard tasks leads to better transfer performance, they restrict learning to the neurons already known to contain the needed information, so this still conflates low-D representations with also using the correct features. I would, in particular, be interested to know if the models trained on hard-only tasks are using a different set of features than the low-D models or are instead using the same low-D set of features plus many more. In the first case, poor transfer is explained by having mismatched features but in the second it would be explained more by having extra features that contribute noise. These differences could also help predict the extent to which one can recover from poor initial training (e.g. do you need to completely relearn or just prune the extra, noisy dimensions). As far as I can tell the current analyses don't speak to this, but given that all models are based on the same AlexNet it would be potentially straightforward to determine if the same neurons are in the top 150 for different learning approaches. I wouldn't say such an analysis is technically required to support the claims of the paper, but I do feel like a part of the story is missing here.

Reviewer #2: The manuscript "The curriculum effect in visual learning: the role of readout dimensionality" by Volk et al. presents a compelling theoretical study of how readout dimensionality influences visual learning and transfer. The authors propose that easy-to-hard curricula promote the learning of low-dimensional solutions, which can then support improved transfer to harder tasks. The study offers an elegant explanation for curriculum learning effects in both artificial neural networks and human learners. Overall, I find the central claim convincing and the work conceptually valuable. That said, I have several methodological questions and points for clarification - If addressed, this would further strengthen the paper.

Major comments

1. Interpretation of curriculum order and interleaving (line 58)

The manuscript states that "interleaving different task conditions can also enhance generalization," but the subsequent discussion emphasizes that task order matters. The cited literature does not clearly establish that order effects differ between interleaved and sequential training. It would be helpful if the authors could clarify whether they are suggesting that both interleaved and sequential curricula enhance generalization, but that the effect is stronger for sequential curricula. If so, this claim may require additional justification or references.

2. Human generalization across sequential and shuffled conditions (line 99)

The observation that humans show similar average generalization improvements in both sequential and shuffled conditions is described as "interesting." This result does seem unexpected given the authors' interpretation that order matters, but it is also consistent with the literature cited earlier (line 58) regarding interleaving benefits. A brief discussion reconciling these two interpretations would improve clarity.

3. Differences between human and model test sets (lines 125, 129)

Humans are tested on different angles at different spatial locations, whereas ANNs are tested on different frequencies. While the authors provide reasonable arguments for these choices, it seems that testing both humans and models on a shared or more closely matched test dimension (e.g., different angles) would have been feasible. Using more comparable test sets could strengthen cross-system comparisons and help isolate whether observed differences arise from distinct learning strategies rather than task design. Perhaps the authors could elaborate a bit more why this was not done or perhaps it could still be done with a reasonable amount of effort.

4. Initialization of readout weights and learning dynamics (lines 156, 646)

The authors argue that learning in the models occurs predominantly through changes in readout weights. However, since the readout weights are initialized to zero and the models are trained via backpropagation, earlier layers cannot receive gradient updates until the readout weights become non-zero. This setup may introduce a bias toward readout-driven learning early in training. To rule out this possibility, it would be informative to repeat key analyses with randomly initialized readout weights matched in scale to other network weights.

5. Identifying influential neurons via readout weights vs. functional contribution

To support the claim that readout subspace dimensionality determines generalization, the authors identify influential neurons by lesioning units with the largest absolute readout weights and measuring the resulting drop in transfer accuracy (ll 310). While this approach is intuitive, large readout weights do not necessarily imply large functional importance for task performance or generalization. A more principled measure of neuron importance would be to assess each neuron's contribution to training or validation loss, for example, by quantifying the increase in loss when a neuron is dropped or masked as done in standard gradient-based attribution techniques. Such an analysis more directly captures functional relevance rather than reliance on weight magnitude alone. Incorporating (or at least discussing) this alternative lesioning criterion would strengthen the link between readout dimensionality and generalization.

6. Correlation between effective rank and transfer accuracy (line 336, Fig. 4E)

The manuscript reports a strong positive correlation (r = 0.95) between effective rank and transfer accuracy. However, according to the stated hypothesis, this relationship should be negative, as is reported elsewhere (e.g., line 332) and as appears visually in Fig. 4E. It is possible that the reported correlation refers instead to the relationship between the easy:hard ratio and transfer accuracy, as described in the figure caption. Clarifying this point and, if necessary, correcting the text would avoid confusion.

7. Performance when training only on the easy task (Fig. 5C)

In Fig. 5C, training exclusively on the easy task appears to yield higher mean transfer accuracy (~0.95) than either the sequential or shuffled curricula. This result is somewhat surprising given the emphasis on curriculum learning. A discussion of why training only on the easy task outperforms the proposed curricula, or whether this difference is statistically reliable, would be valuable.

Minor comments and clarifications

- Terminology: implicit vs. explicit curricula (line 271)

The distinction between a "forced implicit curriculum" and an "explicit curriculum" is unclear, and the terminology appears somewhat inconsistent across the paper. A brief clarification or standardization of terms would improve readability.

- Reporting correlation strength and significance (line 332)

The phrase "negatively correlated" would benefit from a quantitative report of the correlation coefficient and its statistical significance.

- Section references (line 377)

The manuscript refers to "Section 3.5," but the sections are not numbered. Please revise the reference to avoid confusion.

- Figure labeling (Fig. S1B)

The x-axis in Fig. S1B appears to be labeled "Angle separations," but, based on the description, it may be more accurately labeled "Steps."

Reviewer #3: Overall the premise of the paper is interesting. The human data on learning curriculum is a strong part of the paper and the analysis on dimensionality changes with learning curriculum is novel. However, the major weakness lies in the modeling details. The experiments are conducted with a single convolutional ANN architecture, and it is not obvious that the results would generalize to other models. The discussion mentions that testing other model architectures is beyond the scope of this work, however, if the observed changes in representation due to learning are a common motif that will happen in any learning system, then it *should* replicate across other architectures. For this type of study, showing that the results generalize across models is critical – small changes such as learning rate choice, optimizer choice, initialization choice, etc. can have a large effect on the model representation. Given the VPL task and the small number of training steps required, training additional models to verify generalization seems feasible and would significantly strengthen the work. If the authors decide that additional models are infeasible for the study, then the language throughout the paper, including abstract, introduction, etc should be changed to avoid talking about “ANNs” generally and instead talk about the specific model (and architecture) that was used.

128-131: The difference between the types of generalization that are tested in humans and models seems like a confound if the goal of the paper is to directly compare generalization in humans and machines. This is especially concerning given that the reason given is variability across observers, but later on observer variability is highlighted as an interesting feature of the learning curriculum.

127: “convolutional ANNs are equivariant to spatial location” – as far as I can tell from the methods, this is incorrect for the model architecture used for the following reasons: (1) with the described AlexNet-style architecture, the final stage before the fully connected readout layer maintains some spatial information, so the actual readout will not be equivariant to location (2) typical boundary handling operations, like padding, break perfect shift-equivariance (3) operations such as strided convolutions and max pooling result in non-equivariance to small transformations (see https://arxiv.org/pdf/1904.11486 & https://arxiv.org/abs/1805.12177). I understand that this is not the focus of the paper and the text describes what seems like a somewhat minor modeling choice, however given that this is a study primarily about modeling, it is critical that the description of the ANN properties and training setups is technically precise.

154-156: I am concerned that the change in the L2 norms of the weights may be confounded by underlying different scales for the feed-forward convolutional layer weights and the skip connection weights, or potentially the number of parameters that go into the L2-norm. It might be better to normalize the change in weight magnitude by something like the final weight norm. Additionally, how does the learning change if the skip connection weights are initialized at different values other than exactly zero? It is a bit non-standard in deep learning to initialize to exactly zero (small non-zero values can help break symmetries and prevent the network from getting “stuck”) so I was a bit surprised by this choice.

207: Please describe how multiple points for the model results are obtained (I believe that each point corresponds to a different random trial order and different random noise applied during training, but I could not find this detail). Going back to my request at the beginning of the paper regarding different architectures, the results would be much stronger if there were multiple architectures included in the results, for instance if each point were a different pre-trained neural network backbone (including transformers, different CNNs, etc).

300: Additional details on how PCA was performed should be given in the methods. Was any whitening performed on the weights? Were the weights flattened? How were the weights from different readout layers combined?

315, Question: Are the ~150 neurons the same for each of the model instances that are trained, or is a different subset of 150 neurons selected?

746-749: Minor: Even though only one type of trial is used for training, the gradient magnitudes for the other conditions could still be *measured*, but not used for updates.

Line 71 & 109 -- Question: How is a “distinct visual feature” (line 71) or a “dissimilar visual feature” (line 109) defined for the paper? The definition of a “feature” vs. “feature combination” seems important, and from the paper I’m not quite sure what the authors mean.

The authors may want to include a brief discussion on this recent work: https://www.nature.com/articles/s41562-025-02359-3

**********

**Have the authors made all data and (if applicable) computational code underlying the findings in their manuscript fully available?**

The PLOS Data policy requires authors to make all data and code underlying the findings described in their manuscript fully available without restriction, with rare exception (please refer to the Data Availability Statement in the manuscript PDF file). The data and code should be provided as part of the manuscript or its supporting information, or deposited to a public repository. For example, in addition to summary statistics, the data points behind means, medians and variance measures should be available. If there are restrictions on publicly sharing data or code —e.g. participant privacy or use of data from a third party—those must be specified.

Reviewer #1: None

Reviewer #2: **No:**  The authors wrote "All code necessary to reproduce the results are available at the following link for the initial submission: Link." but the "Link" is not clickable (although underlined). Perhaps this is a problem due to the journal's PDF conversion? It would be great if this worked in the final version or if the actual link was included in plain text. Same problem with the raw data.

Reviewer #3: None

**********

PLOS authors have the option to publish the peer review history of their article (what does this mean?). If published, this will include your full peer review and any attached files.

If you choose “no”, your identity will remain anonymous but your review may still be made public.

**Do you want your identity to be public for this peer review?**  For information about this choice, including consent withdrawal, please see our Privacy Policy.

Reviewer #1: No

Reviewer #2: No

Reviewer #3: No

**Figure resubmission:**

While revising your submission, we strongly recommend that you use PLOS’s NAAS tool (https://ngplosjournals.pagemajik.ai/artanalysis) to test your figure files. NAAS can convert your figure files to the TIFF file type and meet basic requirements (such as print size, resolution), or provide you with a report on issues that do not meet our requirements and that NAAS cannot fix.

After uploading your figures to PLOS’s NAAS tool - https://ngplosjournals.pagemajik.ai/artanalysis, NAAS will process the files provided and display the results in the "Uploaded Files" section of the page as the processing is complete. If the uploaded figures meet our requirements (or NAAS is able to fix the files to meet our requirements), the figure will be marked as "fixed" above. If NAAS is unable to fix the files, a red "failed" label will appear above. When NAAS has confirmed that the figure files meet our requirements, please download the file via the download option, and include these NAAS processed figure files when submitting your revised manuscript

**Reproducibility:**

To enhance the reproducibility of your results, we recommend that authors of applicable studies deposit laboratory protocols in protocols.io, where a protocol can be assigned its own identifier (DOI) such that it can be cited independently in the future. Additionally, PLOS ONE offers an option to publish peer-reviewed clinical study protocols. Read more information on sharing protocols at https://plos.org/protocols?utm_medium=editorial-email&utm_source=authorletters&utm_campaign=protocols

# Author comment: Author response to Decision Letter 1

**DOI:** 10.1371/journal.pcbi.1014553.r002

15 Apr 2026

Attachment Submitted filename: Response to Reviewers.pdf

# Peer review: Decision Letter 1

**DOI:** 10.1371/journal.pcbi.1014553.r003

**Authors:** Marieke Karlijn van Vugt, Tim Christian Kietzmann

15 Jun 2026

PCOMPBIOL-D-25-02227R1

The curriculum effect in visual learning: the role of readout dimensionality

PLOS Computational Biology

Dear Dr. Bakhtiari,

Thank you for submitting your manuscript to PLOS Computational Biology. After careful consideration, we feel that it has merit but does not fully meet PLOS Computational Biology's publication criteria as it currently stands. Therefore, we invite you to submit a revised version of the manuscript that addresses the points raised during the review process.

Please submit your revised manuscript by Aug 15 2026 11:59PM. If you will need more time than this to complete your revisions, please reply to this message or contact the journal office at ploscompbiol@plos.org. When you're ready to submit your revision, log on to https://www.editorialmanager.com/pcompbiol/ and select the 'Submissions Needing Revision' folder to locate your manuscript file.

Please include the following items when submitting your revised manuscript:

* A letter that responds to each point raised by the editor and reviewer(s). You should upload this letter as a separate file labeled 'Response to Reviewers'. This file does not need to include responses to formatting updates and technical items listed in the 'Journal Requirements' section below.

* A marked-up copy of your manuscript that highlights changes made to the original version. You should upload this as a separate file labeled 'Revised Manuscript with Track Changes'.

* An unmarked version of your revised paper without tracked changes. You should upload this as a separate file labeled 'Manuscript'.

If you would like to make changes to your financial disclosure, competing interests statement, or data availability statement, please make these updates within the submission form at the time of resubmission. Guidelines for resubmitting your figure files are available below the reviewer comments at the end of this letter.

As the corresponding author, your ORCID iD is verified in the submission system and will appear in the published article. PLOS supports the use of ORCID, and we encourage all coauthors to register for an ORCID iD and use it as well. Please encourage your coauthors to verify their ORCID iD within the submission system before final acceptance, as unverified ORCID iDs will not appear in the published article. Only the individual author can complete the verification step; PLOS staff cannot verify ORCID iDs on behalf of authors.

We look forward to receiving your revised manuscript.

Kind regards,

Tim Christian Kietzmann, Dr. rer. nat.

Academic Editor

PLOS Computational Biology

Marieke van Vugt

Section Editor

PLOS Computational Biology

**Additional Editor Comments (if provided):**

Dear all,

I am happy to let you know that all three reviewers have accepted the paper. I will therefore also accept the paper. Before I can do so, however, I would like to ask you to take note of two minor comments raised by R3, which I would like for you to address quickly before we move on to official acceptance.

Best wishes

Tim

**Journal Requirements:**

If the reviewer comments include a recommendation to cite specific previously published works, please review and evaluate these publications to determine whether they are relevant and should be cited. There is no requirement to cite these works unless the editor has indicated otherwise.

1) In the online submission form, you indicated that All data are available in the main text, supplementary materials, and/or the locations specified in this section. Raw data from human observers are available at the following link for the initial submission: https://drive.google.com/file/d/1ZrHiH7aOwNL4kyeyFmYVIfM5ZZTuwW6B/view?usp=sharing. Data will be made publicly available upon publication. All code necessary to reproduce the results are available at the following link for the initial submission: https://drive.google.com/file/d/115FcLjiCbaP5nyXWNHwEIWmSLJ21K4aR/view?usp=sharing. Code will be made publicly available on Github upon publication and a permanent archive will be created.. All PLOS journals now require all data underlying the findings described in their manuscript to be freely available to other researchers, either

1. In a public repository

2. Within the manuscript itself

3. Uploaded as supplementary information.

This policy applies to all data except where public deposition would breach compliance with the protocol approved by your research ethics board. If your data cannot be made publicly available for ethical or legal reasons (e.g., public availability would compromise patient privacy), please explain your reasons by return email and your exemption request will be escalated to the editor for approval. Your exemption request will be handled independently and will not hold up the peer review process, but will need to be resolved should your manuscript be accepted for publication. One of the Editorial team will then be in touch if there are any issues.

2) Please amend your detailed Financial Disclosure statement. This is published with the article. It must therefore be completed in full sentences and contain the exact wording you wish to be published.

i) State what role the funders took in the study. If the funders had no role in your study, please state: "The funders had no role in study design, data collection and analysis, decision to publish, or preparation of the manuscript."

ii) If any authors received a salary from any of your funders, please state which authors and which funders..

If you did not receive any funding for this study, please simply state: u201cThe authors received no specific funding for this work.u201d

**Reviewers' comments:**

Reviewer's Responses to Questions

**Comments to the Authors:**

**Please note here if the review is uploaded as an attachment.**

Reviewer #1: I appreciate the substantial work the authors put in to update the manuscript.

Reviewer #2: Thanks for addressing all points.

Reviewer #3: The revisions have significantly strengthened the work. I particularly appreciate the addition of the new model architectures, and the results with the reference angle seem much closer to the human behavioral work. It is nice to see that the results generalize in both cases. Overall, the revision has substantially improved the paper. I have two fairly minor suggestions about the architecture results:

Appendix S1: > “However, our results did not extend to other larger and more complex model backbones (see Methods for a complete list of architectures), which are less accurate approximations of visual system architecture and connectivity (24).”

I’m not sure reference 24 is appropriate for this claim, as the paper is about privileged representational axes and not connectivity (and also has a wide range of tests for ResNet50 and ResNets are one of the architecture types that the authors reported as not generalizing).

Lines 739-740: > “We also tried more complex architectures: ConvNeXt, DenseNet121, MNASNet, MobileNetV1/V2, SqueezeNet, ResNet18, and VGG11/16.”

It seems like an additional description of why these results weren’t included would be appropriate, for instance including some of the wording that was given in the rebuttal response. Documenting what didn’t work (and potential reasons why) seems important for reproducibility purposes and future work.

**********

**Have the authors made all data and (if applicable) computational code underlying the findings in their manuscript fully available?**

The PLOS Data policy requires authors to make all data and code underlying the findings described in their manuscript fully available without restriction, with rare exception (please refer to the Data Availability Statement in the manuscript PDF file). The data and code should be provided as part of the manuscript or its supporting information, or deposited to a public repository. For example, in addition to summary statistics, the data points behind means, medians and variance measures should be available. If there are restrictions on publicly sharing data or code —e.g. participant privacy or use of data from a third party—those must be specified.

Reviewer #1: None

Reviewer #2: **No:**  The authors promise to make the data publicly available upon acceptance for the review they provided Google Drive links.

Reviewer #3: Yes

**********

PLOS authors have the option to publish the peer review history of their article (what does this mean?). If published, this will include your full peer review and any attached files.

If you choose “no”, your identity will remain anonymous but your review may still be made public.

**Do you want your identity to be public for this peer review?**  For information about this choice, including consent withdrawal, please see our Privacy Policy.

Reviewer #1: No

Reviewer #2: No

Reviewer #3: No

[NOTE: If reviewer comments were submitted as an attachment file, they will be attached to this email and accessible via the submission site. Please log into your account, locate the manuscript record, and check for the action link "View Attachments". If this link does not appear, there are no attachment files.]

**Figure resubmission:**

-->While revising your submission, we strongly recommend that you use PLOS’s NAAS tool (https://ngplosjournals.pagemajik.ai/artanalysis) to test your figure files. NAAS can convert your figure files to the TIFF file type and meet basic requirements (such as print size, resolution), or provide you with a report on issues that do not meet our requirements and that NAAS cannot fix.

After uploading your figures to PLOS’s NAAS tool - https://ngplosjournals.pagemajik.ai/artanalysis, NAAS will process the files provided and display the results in the "Uploaded Files" section of the page as the processing is complete. If the uploaded figures meet our requirements (or NAAS is able to fix the files to meet our requirements), the figure will be marked as "fixed" above. If NAAS is unable to fix the files, a red "failed" label will appear above. When NAAS has confirmed that the figure files meet our requirements, please download the file via the download option, and include these NAAS processed figure files when submitting your revised manuscript.

**Reproducibility:**

To enhance the reproducibility of your results, we recommend that authors of applicable studies deposit laboratory protocols in protocols.io, where a protocol can be assigned its own identifier (DOI) such that it can be cited independently in the future. Additionally, PLOS ONE offers an option to publish peer-reviewed clinical study protocols. Read more information on sharing protocols at https://plos.org/protocols?utm_medium=editorial-email&utm_source=authorletters&utm_campaign=protocols

# Author comment: Author response to Decision Letter 2

**DOI:** 10.1371/journal.pcbi.1014553.r004

5 Jul 2026

Attachment Submitted filename: Response to Reviewers - 2.pdf

# Editor report: Decision Letter 2

**DOI:** 10.1371/journal.pcbi.1014553.r005

**Authors:** Marieke Karlijn van Vugt, Tim Christian Kietzmann

9 Jul 2026

Dear Dr. Bakhtiari,

We are pleased to inform you that your manuscript 'The curriculum effect in visual learning: the role of readout dimensionality' has been provisionally accepted for publication in PLOS Computational Biology.

Before your manuscript can be formally accepted you will need to complete some formatting changes, which you will receive in a follow up email. A member of our team will be in touch with a set of requests.

Please note that your manuscript will not be scheduled for publication until you have made the required changes, so a swift response is appreciated.

IMPORTANT: The editorial review process is now complete. PLOS will only permit corrections to spelling, formatting or significant scientific errors from this point onwards. Requests for major changes, or any which affect the scientific understanding of your work, will cause delays to the publication date of your manuscript.

Should you, your institution's press office or the journal office choose to press release your paper, you will automatically be opted out of early publication. We ask that you notify us now if you or your institution is planning to press release the article. All press must be co-ordinated with PLOS.

Thank you again for supporting Open Access publishing; we are looking forward to publishing your work in PLOS Computational Biology.

Best regards,

Tim Christian Kietzmann, Dr. rer. nat.

Academic Editor

PLOS Computational Biology

Marieke van Vugt

Section Editor

PLOS Computational Biology

***********************************************************

# Editor report: Acceptance letter

**DOI:** 10.1371/journal.pcbi.1014553.r006

**Authors:** Marieke Karlijn van Vugt, Tim Christian Kietzmann

PCOMPBIOL-D-25-02227R2

The curriculum effect in visual learning: the role of readout dimensionality

Dear Dr Bakhtiari,

I am pleased to inform you that your manuscript has been formally accepted for publication in PLOS Computational Biology. Your manuscript is now with our production department and you will be notified of the publication date in due course.

The corresponding author will soon be receiving a typeset proof for review, to ensure errors have not been introduced during production. Please review the PDF proof of your manuscript carefully, as this is the last chance to correct any errors. Please note that major changes, or those which affect the scientific understanding of the work, will likely cause delays to the publication date of your manuscript.

Soon after your final files are uploaded, unless you have opted out, the early version of your manuscript will be published online. The date of the early version will be your article's publication date. The final article will be published to the same URL, and all versions of the paper will be accessible to readers.

For Research, Software, and Methods articles, you will receive an invoice from PLOS for your publication fee after your manuscript has reached the completed accept phase. If you receive an email requesting payment before acceptance or for any other service, this may be a phishing scheme. Learn how to identify phishing emails and protect your accounts at https://explore.plos.org/phishing.

Thank you again for supporting PLOS Computational Biology and open-access publishing. We are looking forward to publishing your work!

With kind regards,

Janani Seenivasan

PLOS Computational Biology | Carlyle House, Carlyle Road, Cambridge CB4 3DN | United Kingdom ploscompbiol@plos.org | Phone +44 (0) 1223-442824 | ploscompbiol.org | @PLOSCompBiol
